# Addressing the commercial determinants of mental health: an umbrella review of population-level interventions

**DOI:** 10.1093/heapro/daae147

**Published:** 2024-11-21

**Authors:** Alice Tompson, Muhammed Alkasaby, Tahrima Choudhury, Kate Dun-Campbell, Greg Hartwell, Katherine Körner, Nason Maani, May C I van Schalkwyk, Mark Petticrew

**Affiliations:** Department of Public Health, Environments and Society, London School of Hygiene and Tropical Medicine, 15-17 Tavistock Place, Kings Cross, London WC1H 9SH, UK; UK PRP SPECTRUM Consortium, London School of Hygiene and Tropical Medicine, 15-17 Tavistock Place, Kings Cross, London WC1H 9SH, UK; Centre for Global Mental Health, London School of Hygiene and Tropical Medicine, Keppel Street, London WC1E 7HT, UK; Department of Public Health, Environments and Society, London School of Hygiene and Tropical Medicine, 15-17 Tavistock Place, Kings Cross, London WC1H 9SH, UK; NIHR School of Public Health Research, London School of Hygiene and Tropical Medicine, 15-17 Tavistock Place, Kings Cross, London WC1H 9SH, UK; Department of Health Services Research and Policy, London School of Hygiene and Tropical Medicine, 15-17 Tavistock Place, Kings Cross, London WC1H 9SH, UK; Department of Public Health, Environments and Society, London School of Hygiene and Tropical Medicine, 15-17 Tavistock Place, Kings Cross, London WC1H 9SH, UK; Department of Health Services Research and Policy, London School of Hygiene and Tropical Medicine, 15-17 Tavistock Place, Kings Cross, London WC1H 9SH, UK; Global Health Policy Unit, School of Social and Political Science, University of Edinburgh, 15a George Square, Edinburgh EH8 9LD, UK; UK PRP SPECTRUM Consortium, University of Edinburgh, Usher Institute, Old Medical School, University of Edinburgh, Teviot Place, Edinburgh EH8 9AG, UK; Department of Health Services Research and Policy, London School of Hygiene and Tropical Medicine, 15-17 Tavistock Place, Kings Cross, London WC1H 9SH, UK; Department of Public Health, Environments and Society, London School of Hygiene and Tropical Medicine, 15-17 Tavistock Place, Kings Cross, London WC1H 9SH, UK; UK PRP SPECTRUM Consortium, London School of Hygiene and Tropical Medicine, 15-17 Tavistock Place, Kings Cross, London WC1H 9SH, UK

**Keywords:** determinants of health, mental health, tobacco, alcohol, gambling, evidence synthesis

## Abstract

There is increasing evidence that commercial determinants impact mental health. Addressing the commercial determinants may therefore be a way of improving population-level mental health. This umbrella review aimed to provide an overview of evidence in this field and identify knowledge gaps. Five databases (MEDLINE, Embase, PsycINFO, Scopus and Cochrane Library) were searched on the 18/19 of July 2022. Eligible papers were systematic reviews published after 31 December 2011. No geographical limits were applied. Eligible interventions were those that targeted the behaviours or products of commercial actors. Ineligible interventions included individual behaviour change interventions, such as those seeking to educate consumers. Included mental health outcomes were anxiety, depression, self-harm and suicide, whilst surrogate outcomes included product consumption. Industry involvement and the quality of included reviews (critical components of A MeaSurement Tool to Assess systematic Reviews - AMSTAR 2) were assessed. A narrative synthesis was used to compare the findings by industry, and a typology of interventions was developed. Eight reviews with mental health outcomes were included, each with multiple methodological weaknesses. There is some evidence that reducing the availability of alcohol or pesticides may lower suicide rates. Despite the known links, no evidence on the mental health impacts of population-level interventions tackling the social media, tobacco, gambling and ultra-processed foods industries were located. All gambling reviews were identified as having links to industry. Future high-quality evaluations of commercial determinants interventions developed specifically with the aim of achieving positive mental health outcomes and/or that evaluate mental health outcomes and are free from industry links are needed.

PROSPERO ref. number CRD42022346002.

**Contribution to Health Promotion**

- This project mapped the available evidence regarding the mental health impacts of using population-level interventions to address the commercial determinants of health.
- We used a novel protocol to assess industry involvement in the located reviews and developed a typology of interventions.
- We found some evidence that reducing the availability of alcohol or pesticides reduces suicide rates.
- We located no reviews of interventions addressing the social media, tobacco or gambling industries that measured mental health outcomes, despite the known links.
- Addressing commercial determinants is a currently under-evaluated approach for improving population mental health, and this project highlights current research gaps.

## BACKGROUND

Mental disorders—such as anxiety and depression—are amongst the leading causes of ill health worldwide with nearly 1 billion people affected—an increase of 48% between 1990 and 2019 (GBD 2019 Mental Disorders Collaborators, 2022). People with mental health disorders experience substantial inequalities in life expectancy, which encompass a wide range of diagnoses (Lawrence *et al.*, 2013; Chan *et al.*, 2023). In addition to significant negative mental and physical health impacts, the growing burden of mental disorders has major social, economic and human rights consequences (WHO, 2019). Mental ill health is distributed unequally across and within societies. It also acts as a determinant of health and other social and economic outcomes, further exacerbating inequalities (Friedli, 2009).

### Commercial determinants of health

As part of the efforts to understand and address the wider determinants of ill health, there has been growing attention paid to the role of commercial interests in shaping health and health inequalities (de Lacy-Vawdon and Livingstone, 2020; Mialon, 2020). Initial efforts focussed on industry activities seeking to increase the consumption of unhealthy products—such as tobacco, alcohol and ultra-processed foods (UPFs)—which fuel epidemics of non-communicable diseases (Stuckler *et al*., 2012; Millar, 2013). More recent endeavours have looked beyond the consumption of unhealthy products to consider, for example, the gambling, fossil fuel and social media industries (van Schalkwyk *et al*., 2021b; Maani *et al*., 2022; Zenone *et al*., 2022), as well as the diffuse ways in which powerful corporate actors shape social, physical and cultural environments (de Lacy-Vawdon and Livingstone, 2020; Lee and Crosbie, 2020; Mialon *et al*., 2020). This diversification in interests is reflected in a recent definition of the commercial determinants of health as, ‘*the systems, practices, and pathways through which commercial actors drive health and equity*’ (Gilmore *et al.*, 2023).

### Commercial determinants of mental health

A recently published sister overview of this project synthesized the evidence regarding the mental health impacts of six key industries known to affect health (Dun-Campbell *et al*., 2024). For example, regular alcohol consumption is known to affect the balance of neural transmitters in the brain and increases the risk of developing depression (RCPSYCH, 2019) A meta-analysis of prospective cohort studies found a dose–response effect, with heavy drinkers having a 13% higher risk of subsequently developing depressive symptoms compared to non-drinkers (relative risk 1.13; 95% confidence interval 1.05–1.22) (Li *et al.*, 2020). There is also rising concern regarding social media use and mental health, especially that of young people (Office of the Surgeon General, 2023). A number of pathways linking the two have been proposed including altered sleep quality, cyberbullying and sexting (Dun-Campbell *et al*., 2024).

Addressing the commercial determinants of mental health (CDOMH) is an essential public health endeavour (Freudenberg *et al.*, 2021). However, to date, initiatives seeking to improve public mental health and reduce mental health inequalities have largely overlooked commercial influences (BMA, 2018; PMHIC, 2022; van Schalkwyk *et al*., 2023). Therefore, this overview seeks to collate evidence regarding the mental health impacts of interventions addressing the commercial determinants of health. In doing so, it will also identify gaps in the existing evidence base, avenues for future research and potential leverage points for future interventions.

## METHODS

An umbrella review (review of systematic reviews) was conducted to provide an overview of evidence in this field to inform policy and practice and future research (Papatheodorou, 2019). It followed a similar approach to that of Dun-Campbell et al. (Dun-Campbell *et al.*, 2024) by considering six key industries known to impact health: alcohol, tobacco, gambling, social media, UPF and pesticides. These industries were selected because each involves powerful, multinational commercial actors who deploy a portfolio of strategic activities to generate profits and increase the consumption of widely used, everyday products (Knai *et al.*, 2021; Zenone *et al.*, 2022). Most products have addictive qualities with some (alcohol, tobacco, UPF) used to self-medicate to ease the symptoms of mental ill health, whilst pesticides are a high-lethal means of suicide (van Schalkwyk *et al.*, 2023). Social media is included as it is a rapidly developing field both in terms of the technologies involved and the evidence base regarding its impact on health (Sharma *et al*., 2020).

This report was prepared in accordance with the PRISMA (Preferred Reporting Items for Systematic Reviews and Meta-Analyses) reporting guideline (Page *et al*., 2021). A completed checklist is provided in Supplementary File 1. The protocol was pre-registered on PROSPERO (ref. number CRD42022346002).

### Deviations from protocol

It was initially planned to include evidence from high-income settings only. However, due to the paucity of mental health data identified by initial searches, a *post hoc* decision was made to remove this inclusion criterion.

The review also set out to explore interventions addressing the impact of the fossil fuel industry. This was intended as a boundary case, exploring whether our approach worked for an industry with many externalities and whose products are embedded in modern life. Access to affordable fossil fuels currently acts a social determinant of health—for example by heating homes—and initiatives to reduce energy consumption may unintentionally widen health inequalities (Maani *et al*., 2021). Ultimately, fossil fuels proved too different to fit into the emerging typology of interventions and, due to time constraints, the identified literature was not fully screened for inclusion.

The review also intended to explore the mental health impact of interventions that address more distal ways in which commercial actors and corporations impact our living, working and environmental conditions at meso and macro levels (Freudenberg *et al*., 2021; Lee *et al.*, 2022), following a similar approach as that adopted by Naik (Naik *et al.*, 2019) for physical health. However, space constraints preclude the reporting of this body of research here.

### Eligibility criteria

Eligible studies were systematic reviews—or reviews with a systematic search strategy—that considered populations of any age and in any geographical setting.

Interventions had to address the commercial determinants of health at a population level through changing the actions of any of the six industries listed above. Interventions that targeted population groups defined by their clinical characteristics (e.g. those with cancer), were delivered in clinical settings or focused on changing consumer behaviour rather than corporate behaviour were excluded. Supplementary File 2 provides examples of eligible and ineligible interventions.

Primary outcomes were changes in mental health; specifically, anxiety, depression, self-harm and suicide. Any type of measure (e.g. self-reported or clinician assessed) for these outcomes was eligible. Following the reviewers’ comments, a *post hoc* decision was made to exclude reviews that reported emotional responses, such as feeling anxious, in response to interventions like warning labels on health-harming products.

Surrogate outcomes considered the pathways through which commercial activities may impact mental health, for example, via the consumption of unhealthy commodities and through mediators such as stigma, violence and obesity.

### Search strategy and information sources

Based on the inclusion criteria and informed by the strategies of existing reviews examining these industries, a search strategy for MEDLINE was developed with MeSH and free-text search terms. This was adapted for use in Embase, PsycINFO, Scopus and the Cochrane Library. Filters were applied to include only human studies, review articles and those in English (due to the language proficiencies of the research team). Searches were limited to articles published after 31 December 2011 for pragmatic reasons, with searches run on 18/19 July 2022. The full search strategies were reviewed by a specialist librarian and are provided in Supplementary File 3. The reference lists of relevant overviews identified by the search were screened for eligible systematic reviews.

### Selection process

Initial screening of titles and abstracts against the inclusion and exclusion criteria was undertaken by a single reviewer (A.T.). Full-text screening was undertaken independently by two researchers and any discrepancies resolved by discussion.

### Quality appraisal

Quality appraisal was undertaken using the critical components of A MeaSurement Tool to Assess systematic Reviews 2 (AMSTAR 2) (Shea *et al*., 2017) for included reviews. Ten per cent of included reviews were independently double-appraised (A.T. and M.A.), with any discrepancies resolved. Following this pilot, the remaining reviews were single-appraised.

### Data extraction

Data extraction was undertaken by a single reviewer (A.T.) using an Excel spreadsheet. It was checked by a second reviewer (M.A.), and any discrepancies were discussed and resolved.

For reviews with mental health outcomes, details regarding the review type, analysis method, population, setting, interventions and outcomes, number of included studies and number of relevant studies were extracted. Synthesized results applicable to our research interest were also noted. Within each review, the details of studies relevant to our study (i.e. interventions addressing the commercial determinants of health and with mental health outcomes) were also collated, based on the data available in the review. This included study design and effect measures. Primary study reports were not consulted. Details about how interventions might impact mental health inequalities were sought by extracting data on the PROGRESS-Plus framework characteristics (O’Neill *et al.*, 2014).

For reviews with surrogate outcomes, details of eligible interventions and outcomes were extracted.

### Synthesis

A narrative synthesis was undertaken, comparing the reviews grouped by industry. If reported, any change in mental health outcome associated with the intervention was reported.

For all identified reviews, a typology was developed, informed by the Nuffield ladder of public health interventions (Nuffield Council on Bioethics, 2007). Interventions were grouped depending on whether they adopted a population-level approach or concentrated on altering harmful product characteristics or their private sector consumption environments. These findings were tabulated and frequencies calculated.

### Assessing links to industry

Lauber et al. reported a protocol for assessing the involvement of industry in research was adapted to rate the identified reviews (Lauber *et al.*, 2021). Funding details and declarations of interests were extracted and, if available, published protocols were also checked for these details. If not present, or if the authors declared no conflicts of interest, further searches were undertaken regarding the first, second, last and corresponding authors: institutional and ORCID profiles were searched for curriculum vitae and/or grant details covering the year of publication of the review and 2 years prior; contemporaneous publications related to the same industry as the included review were also searched for, and conflicts of interest statements were screened. The collation of this information and initial rating was undertaken by a single researcher (A.T.), and the data and rating were checked by a second (M.P.).

The UPF reviews were treated differently following discussion amongst the authors: this is because it is contested as to whether working with all food industry partners is inappropriate (Gornall, 2015; Jebb, 2015). For example, the health-harming impacts of sugar-sweetened beverages companies are widely acknowledged; however, the role of supermarkets who may facilitate access to fresh fruit and vegetables—in addition to sugar-sweetened beverages, tobacco and alcohol—is less clear cut. Therefore, for these reviews, only the conflict of interest and funding statements provided were screened. No additional checks on the authors were undertaken. Caution should be made when comparing these findings to the industry ratings carried out for the other industries.

### Ethical approval

Institutional ethical approval for this study was not required, as it analyses previously published data.

## RESULTS

### Study selection

**Fig. 1:**
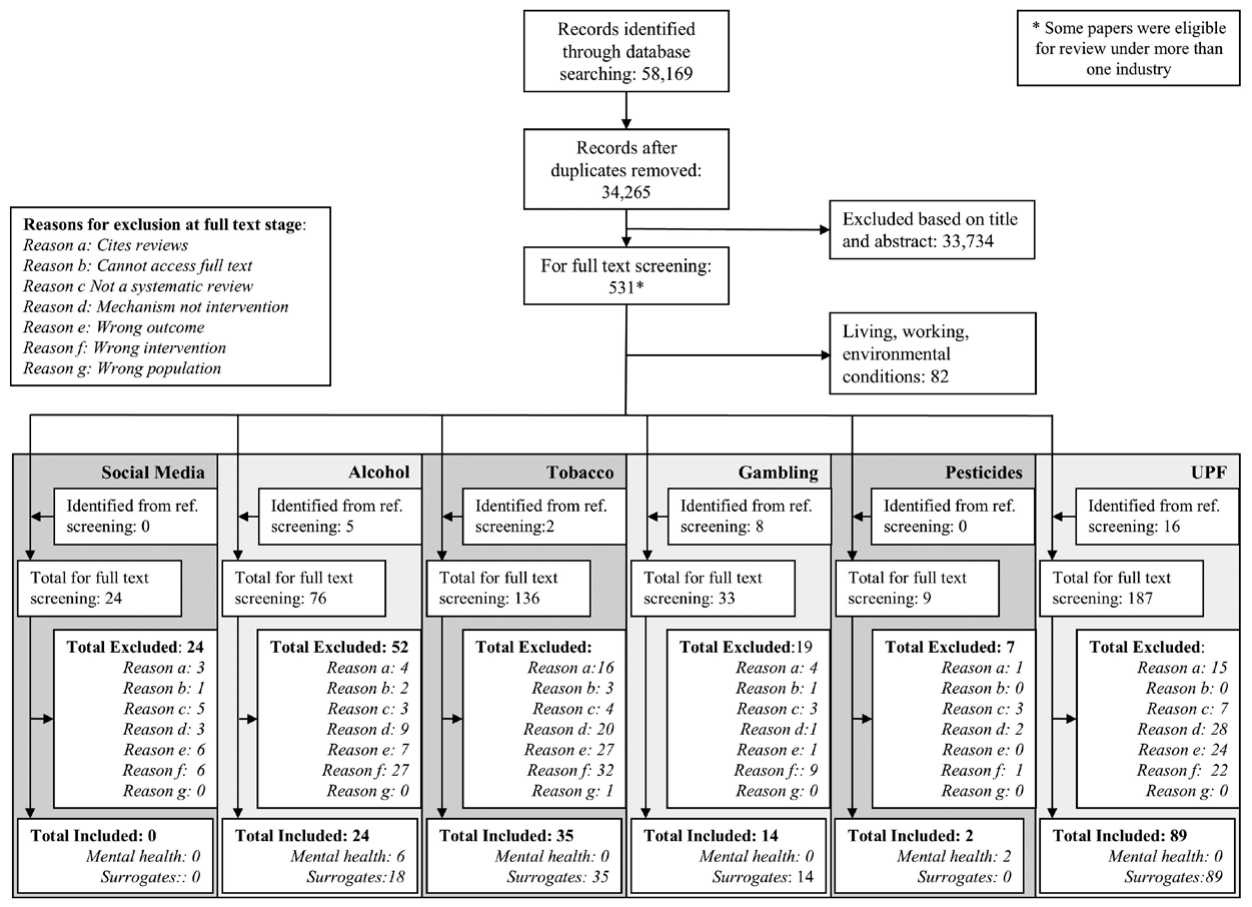
PRISMA flow diagram.

Figure 1 describes the results of the search and selection process: once de-duplicated 34 265 unique records were identified by the searches. Following title and abstract screening, 531 records went forward to full-text screening, with some reviews eligible for consideration for multiple industries. Supplementary File 4 lists the reviews that were excluded at the full-text screening stage and provides the reason for their exclusion.

Overall, 162 reviews were included. No eligible reviews regarding the social media industry with either mental health or surrogate outcomes were identified: twenty-four reviews were full-text screened but, for example, were found to lack a systematic search strategy (*n* = 6) or described a mechanism by which mental health is impacted that could be addressed by future interventions such as cyberbullying, rather than evaluations of interventions themselves (*n* = 3).

### Reviews with mental health outcomes

Of the 162 included reviews, eight reported mental health outcomes; Table 1 presents their characteristics. None of the reviews of interventions aimed at addressing the harms of the gambling or tobacco industry or UPF reported mental health outcomes.

**Table 1:** Characteristics of included reviews with mental health outcomes (*n* = 8)

Lead author (year)	Review objective	Population/setting	Eligible interventions	Eligible study designs	Studies (*n*), total, eligible	MH outcome			
						Anxiety	Depression	Self-harm	Suicide
Alcohol (*n* = 6)									
Roodbeen *et al.* (2021)	‘*to present a broad spectrum of intended as well as unintended units of information derived from the literature relative to the impact of a raise in the minimum legal drinking age on primary & secondary societal harm & violence*’	NS/NS	Raising minimum legal drinking age	NS	91, 4	—	—	—	Yes
Kolves (2020)	‘*to conduct a systematic literature review of the impact of alcohol policies at the population level on suicidal behavior & ideation*’	NS/NS	Availability, pricing policies	Ecological-level studies	19, 11	—	—	—	Yes
Muhunthan (2017)	‘*to investigate how Indigenous communities use public health law mechanisms to control alcohol & prevent its misuse & to what extent controls are effective in achieving improvements in health & social outcomes*’	Indigenous communities/NS	Community-led alcohol control policy models	Quantitative study designs	18, 4	—	—	Yes	Yes
Nepal (2020)	‘*to assess the effects of extensions & restrictions in trading hours of on- & off-license alcohol outlets*’	NS/NS	Alcohol trading hours	Randomized/non-randomized with control site; controlled before-and-after studies, interrupted timeseries	22, 1			Yes*	
						*Alcohol-related hospital admissions either for mental health and behavioural disorders/toxic effect of alcohol			
Nelson (2016)	‘*To summarize before-after effects of important price–tax changes on alcohol-related harms, & to compare these outcomes across countries in order to generalize about effects of important changes in alcohol prices on measures of harmful health outcomes for various subpopulations*’	NS/nine countries	Alcohol taxes, prices	Natural experiments	45, 1	—	—	—	Yes
Hahn *et al.* (2012)	‘*To evaluate the effects of the privatization of alcohol retail sales on excessive alcohol consumption & related harms*’	NS/NS	Monopolization of retail sales	NS	14, 1	—	—	—	Yes
Pesticides (*n* = 2)									
Gunnell (2017)	‘*To review the evidence of the effectiveness of pesticide regulation in reducing the incidence of pesticide suicides & overall suicides*’	NS/NS	Bans, sales restrictions	Natural experiments and controlled intervention (randomized and non-randomized)	27, 27	—	—	—	Yes
Reifels (2019)	‘*To identify effective community based suicide prevention approaches that involve restricting access to pesticides*’	NS/NS	Community-based suicide prevention approaches that involve restricting access to pesticides	Before-and-after, time-series analyses, quasi-experimental studies or RCTs	5, 2	—	—	—	Yes

NS: not specified; RCTs: randomized controlled trials.

All of the reviews listed in Table 1 used narrative methods. None explicitly discussed their findings in terms of mental health inequalities, although some reported their findings stratified by demographic groups (e.g. by gender or age).

Table 2 reports the results of the quality appraisal and links with industry assessment. All were assessed to have multiple methodological limitations with few having pre-published protocols or undertaking risk of bias assessments. Supplementary File 5 reports the full quality appraisal assessment. Most of the reviews—apart from an alcohol-related one (Nelson, 2016) and the two identified for the pesticide industry (Gunnell *et al.*, 2017; Reifels *et al.*, 2019)—were rated as apparently free from links to industry. Of these, two were funded, at least partly, by industry sources (Nelson, 2016; Reifels *et al.*, 2019), and two co-authors of the other declared previous industry funding (Gunnell *et al.*, 2017; Table 2).

**Table 2:** Results of the quality appraisal and industry involvement rating for the included reviews with mental health outcomes (*n* = 8)

Author (year)	AMSTAR-2 critical component							Industry involvement assessment	
	2	4	7	9	11	13	15	Rating	Rationale
Alcohol									
Roodbeen (2021)	No	No	No	No	NA	No	NA	Apparently independent	
Kolves (2020)	No	No	No	Yes	NA	Yes	NA	Apparently independent	
Nepal (2020)	Partial yes	Partial yes	No	Yes	NA	Yes	NA	Apparently independent	
Muhunthan (2017)	No	Partial yes	No	No	NA	No	NA	Apparently independent	
Nelson (2016)	No	No	No	No	NA	No	NA	Study funded by industry sources	Funding by International Center for Alcohol Policies
Hahn (2012)	No	No	No	Yes	NA	Yes	NA	Apparently independent	
Pesticides									
Gunnell (2017)	Yes	No	No	Yes	NA	Yes	NA	Declared researcher links with industry	D.G. was a member/chaired three scientific advisory groups for Syngenta-funded studies, receiving travel expenses (2003–11). M.E. received travel fees from Syngenta prior to 2007
Reifels (2019)	No	No	No	No	NA	Yes	NA	Study partially funded by industry sources	Syngenta provided travel expenses for co-authors to attend a meeting, where the review protocol was developed

AMSTAR-2 Component: 2 = pre-registered protocol; 4 = comprehensive search strategy; 7 = excluded studies listed with justification; 9 = risk of bias assessment; 11 = appropriate meta-analysis methods; 13 = accounted for risk of bias when interpreting/discussing the results; 15 = assessed publication bias.

NA: not applicable.

#### Alcohol

Six partially relevant reviews were identified, the most for any industry considered. However, none reported anxiety or depression as outcomes, nor cited data from the global south. The reviews considered the effects of interventions altering the affordability and availability of alcohol (including raising the minimum legal drinking age, reducing hours of opening and/or density of retail outlets). Data for self-harm or suicide were typically presented alongside that for alcohol-induced crimes such as violent and/or drink driving.

Three of the six reviews included multiple relevant studies. Roodbeen et al. identified four studies, which examined the impact of raising the minimum legal drinking age on suicide: two reported a decrease in rates (Jones *et al*., 1992; Birckmayer and Hemenway, 1999), whilst two reported no effect (Hingson *et al*., 1985; Grucza *et al*., 2012) the latter studied women only (Roodbeen *et al.*, 2021). Kolves et al. examined the impact of a range of alcohol policies on suicide; 11 studies (Skog, 1993; Joubert, 1994; Sloan *et al.*, 1994; Birckmayer and Hemenway, 1999; Berman *et al.*, 2000; Markowitz *et al.*, 2003; Yamasaki *et al.*, 2005; Wood and Gruenewald, 2006; Pridemore and Snowden, 2009; Son and Topyan, 2011; Pridemore *et al.*, 2013) described interventions relevant to this review (Kolves *et al.*, 2020). They concluded that, ‘*although the methods and effect sizes varied substantially in the studies, reducing alcohol often led to reduction in suicidal behaviour… Policies targeting harmful alcohol consumption may contribute towards a reduction in suicidal behavior at the population level*’. Muhunthan et al. reported outcome data for three of the four studies they included that considered suicide (Lee, 1993; Wood and Gruenewald, 2006; Berman, 2014) and for one study that measured self-harm (Wood and Gruenewald, 2006): these findings suggest that total prohibition of alcohol may be an ineffective intervention compared to partial restrictions (Muhunthan *et al.*, 2017).

The remaining three reviews each cited a single primary study relevant to this overview: Nepal et al. reported a study that found a decrease in the incidence of alcohol-related hospitalization, including for mental health and behavioural disorders, following trading hour restrictions for off-premise alcohol sales (Marcus and Siedler, 2015; Nepal *et al.*, 2020). Hahn et al. found that the remonopolization of alcohol sales (and the accompanying fall in accessibility) was followed by a reduction in suicides (Ramstedt, 2002; Hahn *et al.*, 2012). Meanwhile, Nelson and McNall reported that price increases were associated with reduced suicide rates (Cook and Durrance, 2013; Nelson and McNall, 2016).

#### Pesticides

Two reviews (Gunnell *et al*., 2017; Reifels *et al*., 2019) were identified regarding the pesticide industry (one fully relevant, one partially relevant, both with acknowledged industry links). Between them, they cited 28 unique studies (one was included in both) that considered the impact of bans and sales restrictions and were conducted in settings around the globe. The fully relevant review (Gunnell *et al.*, 2017) concluded, ‘*National bans on highly hazardous pesticides…seem to be effective in reducing pesticide-specific and overall suicide rates. Evidence is less consistent for sales restrictions*’.

#### Surrogate outcomes

Supplementary File 6 describes the included reviews with surrogate outcomes: there were 18 reviews for alcohol, 35 for tobacco, 14 for gambling and 89 for UPF with the outcomes typically related to product consumption or sales. No reviews with surrogate outcomes were identified for social media or pesticides.

**Fig. 2:**
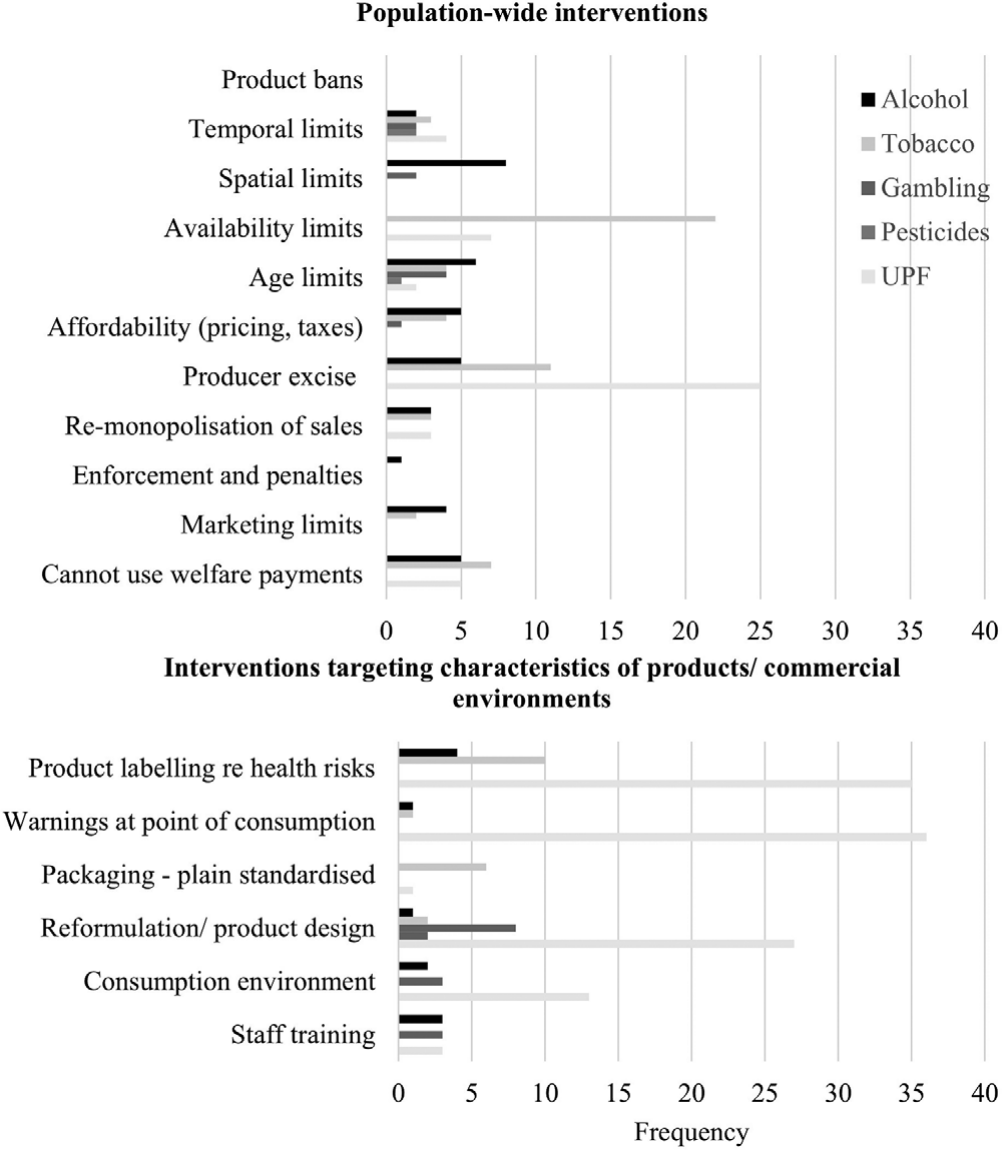
A typology of interventions considered by the identified reviews (*n* = 162, reviews may consider multiple interventions).

Figure 2 maps the interventions considered by these reviews, along with those evaluated for mental health outcomes, and groups them by whether they adopt a population-based or a product-based/private sector approach. It reveals differences between industries; for example, alcohol has the broadest portfolio of interventions evaluated, including a variety of population-wide interventions. For tobacco, the findings reflect the interest in smoke-free places in the last decade or so, along with price rises and health warnings on cigarette packets. Figure 2 also reveals that there have been multiple reviews concerned with potentially altering the harmfulness of gambling products through modifying certain features, for example including pop-up messages. Meanwhile, few population-wide interventions—such as placing marketing limits on the gambling industry or reducing affordability—have been evaluated. The UPF literature also includes a discussion of product re-design as an intervention. However, this has been complemented by evaluating other interventions such as industry levies—notably on sugar-sweetened beverages—and product and menu labelling. The small number of pesticides reviews prevents interpretation.

### Links to industry

All tobacco reviews (*n* = 35) were concluded to be free from links to the tobacco industry; conversely no gambling (*n* = 14) or pesticide (*n* = 2) reviews were felt to be completely independent of industry, with the type of industry links, and reporting of these, diverging across reviews. For alcohol, nearly 80% of reviews (19/24) were assessed as being independent from the alcohol industry. In terms of the additional checks conducted, these located four reviews with industry links that were undeclared [gambling (*n* = 3); alcohol (*n* = 1)].

Across the gambling, pesticides and alcohol reviews (*n* = 40), four (10%) were directly funded by industry actors (three by gambling operators, one partly funded by a pesticide manufacturer); eight (20%) were funded by organizations that receive funding from the proceeds of gambling or alcohol sales (five gambling reviews—e.g. Gambling Research Exchange Ontario, GambleAware; three alcohol reviews—e.g. International Center for Alcohol Policies, International Alliance for Responsible Drinking) and the authors of nine further reviews were assessed as having links to industry (e.g. via other research funding or consultancy work).

For the abridged protocol used for UPF, 15/89 (17%) reviews lacked a conflict of interest and/or funding statement, making it impossible to rate industry involvement. Fifty-six (63%) were assessed as being apparently independent, whilst three (3%) reported being directly funded by the food industry. The research teams of the remaining 15 (17%) reviews described some links to the food or weight loss industry.

Table 1 reports the assessment findings at a review level for those with mental health outcomes, and Supplementary File 6 reports this information for reviews with surrogate outcomes.

## DISCUSSION

### Summary of results

This overview located a few reviews evaluating population-level interventions that measure common mental health outcomes. No reviews were identified that assessed the impact of these industries on mental health inequalities. These represent important gaps in our understanding given (i) the increasing understanding of the impact that commercial determinants have on mental health (Dun-Campbell *et al*., 2024) and (ii) and the role played by commercial actors in driving health inequalities (Collin and Hill, 2015).

Some evidence that reducing the availability of alcohol or pesticides may lower suicide rates was located by this overview. Beyond pesticides, we found very little evidence from low- and middle-income countries. This is despite populations in these settings experiencing similar levels of mental ill health as high-income settings (WHO, 2022) and being targeted by unhealthy commodity industries who are keen to expand into markets with fewer public health restrictions (Abdalla *et al.*, 2022). Therefore, further research into addressing the CDOMH in low- and middle-income countries should also be a priority.

The limited evidence base that measured mental health outcomes was largely concerned with the impact of interventions on suicide rates. Meanwhile, no reviews considered depression, a common mental health condition that affects the lives of many people around the world. For alcohol, tobacco, gambling and UPF, we identified greater numbers of reviews of interventions that measured surrogate outcomes, such as unhealthy product consumption. Future work could extrapolate the findings of this body of literature to model the potential impact on mental health outcomes of these interventions, drawing on our improving aetiological understanding (Dun-Campbell *et al*., 2024).

### Alcohol

The industry for which the greatest body of evidence regarding mental health outcomes was identified was alcohol (*n* = 6), with suicide being the mostly commonly reported outcome. No alcohol reviews were located that measured the impact of controls on anxiety or depression. The potential benefit on population levels of depression remains an unevaluated—and potentially underutilized leverage point—when seeking to address the influence of the alcohol industry on society.

It was striking that the suicide data in the alcohol reviews were sometimes reported alongside criminal outcomes (such as drink driving and assault) and not framed as a mental health concern. These reviews, published since 2010, therefore reinforce outdated and stigmatizing representations of suicide, which was decriminalized in the UK in 1961 (Kelly and Dale, 2011). Such framings may also inadvertently reinforce industry playbook messaging that blames harms to health on troubled individuals irresponsibly consuming their products, rather than their products themselves and their associated corporate activities (van Schalkwyk *et al*., 2021a). To avoid this and other problematic framings in the future, researchers must work with relevant patient groups to ensure the perspectives and voices of marginalized groups are at the heart of their work (Bareham *et al.*, 2023).

### Gambling

The harm to mental health that can be caused by gambling is well known: for example, PHE estimate that 400 suicides (PHE, 2021) each year in the UK are attributable to gambling harms, and there is growing public concern about the issue (Davies, 2022). However, we were unable to find any eligible gambling reviews with mental health outcomes. We did find, however, that all of the located gambling reviews with surrogate outcomes had links to industry. The gambling industry has been the main funder of gambling research for over 40 years, and this has resulted in an evidence base that is narrow in scope and that focuses on problematizing individuals whilst deflecting attention from harmful products and industry practices (van Schalkwyk *et al.*, 2021b; van Schalkwyk and Cassidy, 2022). This review illustrates the repercussions of this—an evidence base aligned with industry priorities and discourses.

It has been argued that the industry-promoted paradigm of ‘responsible gambling’ exacerbates these feelings of shame through its foregrounding of individual failings as the cause of uncontrolled gambling (Rintoul *et al*., 2023). The circulating narrative of personal responsibility also steers research and policy responses towards behavioural interventions with the whole population, public health-type approaches neglected (van Schalkwyk *et al.*, 2021b). Noting the lack of evidence in this area, Regan et al. used a Delphi process to obtain consensus amongst experts—some with industry links—on control measures perceived as effective that could be implemented successfully (Regan *et al.*, 2022). Unlike the evidence base we identified, the proposed interventions spanned domains such as price and taxation, availability, marketing, advertising, promotion, sponsorship and the environment. Evaluations of interventions such as these which measure mental health outcomes are urgently needed to diversify the portfolio of evidence available to policy makers seeking to prevent gambling harms.

### Tobacco

There is high-quality evidence linking smoking to depression and suicide (Dun-Campbell *et al*., 2024), and a Cochrane review concluded that there is very low- to moderate-certainty evidence that smoking cessation is associated with small to moderate improvements in mental health (Taylor *et al.*, 2021). During screening for this project, one review (Drovandi *et al*., 2018) cited one study conducted in Nigeria that reported the percentage of students experiencing the emotional response of anxiety induced by different tobacco warning messages (Adebiyi *et al*., 2016). Following the advice of the reviewers, a *post hoc* decision was made to exclude this outcome, as it would have a very limited impact on mental health. It does, however, act as a reminder of the effects that public health interventions can have on mental health. In turn, this needs to be balanced against the aforementioned wider evidence of the mental health harms from products such as tobacco. Elsewhere, public health initiatives have sought to change behaviour through inducing peer pressure (e.g. social pledges to reduce antibiotic use (Kesten *et al*., 2017)] or shame [e.g. the UK Government’s ‘look them in the eyes’ campaign during the COVID-19 pandemic (DHSC, 2021)] or by de-normalizing/stigmatizing product consumption (Bayer, 2008), for example, through smoke-free spaces and plans for smoke-free generations (Iacobucci, 2023). Ensuring evaluations measure intended and unintended—mental health impacts will enable the quantification of their magnitude, duration and clinical significance. This information will also help counter industry efforts to lobby against strengthened public health controls citing concerns about potential mental health harms.

### Digital spaces: social media, gambling and other industries

We identified no eligible reviews of social media interventions, either with mental health or surrogate outcomes. Screened interventions included educating children about cyberbullying, for example, but this was ineligible as they targeted consumer behaviour rather than addressing the upstream CDOMH. The lack of evaluations of eligible interventions may reflect the limited appetite from social media companies to allow public health evaluators access to their digital environments and/or to alter their products, which have been carefully and deliberately designed to maximize consumption and, therefore, advertising revenue (Zenone *et al*., 2022).

The issue of digital spaces extends beyond social media to include gambling. In the UK, remote gambling generated a gross yield of £6.4 billion in 2021/2 (45% of the industry’s total) (The Gambling Commission, 2022), and yet no identified reviews considered eligible online gambling controls. A recent systematic review—funded by Svenska Spel, a publicly governed gambling operator—examined the evidence for preventative interventions in electronic and online gambling (Fiskaali *et al*., 2023). Whilst five trials were located, their interventions all addressed consumer behaviour and were not eligible for this review.

Health-harming digital spaces extend to online retailers of alcohol, tobacco and UPF. Recent developments include the advent of delivery-only ‘dark kitchens’ and rapid grocery delivery services (Rinaldi *et al*., 2022) with matters further complicated by the advertising and promotion of harmful products via social media (Torrance *et al*., 2021). ‘Traditional’ public health controls such as local authority licensing or planning controls in the UK have operated on a placed-based basis, and a diversification of approaches is needed to protect health and fully address the commercial determinants of health.

### Ultra-processed foods

We did not identify any reviews of interventions addressing the UPF industry that measured mental health. This may reflect that the biological causal pathways between the two are less well elucidated. Proposed mechanisms that may lead to depression—and identified by a recent umbrella review—include systemic inflammation, disturbance to the gut microbiota, disrupted dopamine function, insulin resistance and oxidative stress (Dun-Campbell *et al*., 2024).

We did, however, identify a large body of evidence that measured the impact on UPF consumption, reflecting the public health concern regarding obesity and other non-communicable diseases. The causes of obesity are complex and include a bidirectional relationship with mental health (Steptoe and Frank, 2023). A recent international study using systems mapping approaches with young people to understand the drivers of obesity identified three feedback loops relevant to this project: the commercial drivers of unhealthy diets; mental health and unhealthy diets; and social media use, body image and motivation to exercise (Savona *et al*., 2021).

We deployed an abridged protocol for assessing conflicts of interest of the UPF reviews and did not undertake additional conflicts of interest checks for review authors. In their analysis of Coca-Cola-funded research, Serôdio *et al.* found that several authors failed to declare the receipt of such funding, suggesting a limitation of our UPF approach (Serôdio *et al.*, 2018).

The rationale for our decision was that not all food industry products or actions are viewed as harmful. Similarly, it might be argued that the social media industry is also an ambiguous actor with products reducing social isolation or sharing mental health promotion materials. The tensions in classifying actors as ‘bad’ and/or ‘good’ are being discussed more broadly within the commercial determinants of the health field, with some proposing a more nuanced categorization (Lacy-Nichols *et al*., 2023) or neutral framing (Gilmore *et al.*, 2023). These reflections may help to inform future efforts to assess researchers’ conflicts of interest with respect to links to industry.

### Embracing complexity

Systems-type thinking—as used by Savona et al. (Savona *et al.*, 2021)—has been successfully used to further our understanding of obesity, and it has been argued that the study of mental health would benefit from similar approaches (Wolpert, 2018). For example, Stansfield et al. used systems mapping to develop an organization-wide approach to public mental health (Stansfield *et al.*, 2021). Recognizing the growing awareness of the socio-ecological determinants of public mental health (Lund *et al*., 2018; Kousoulis and Goldie, 2021), they organize their map into individual, social-community, societal-economic, physical-behavioural and environmental areas. Whilst the commercial determinants influence several of these domains, their role is not explicitly described. As has been noted for physical health, the overlooking of the commercial determinants from public health frameworks can mean they are omitted from efforts to improve health (Maani *et al.*, 2020b). Therefore, future systems mapping activities might seek to explicitly explore the CDOMH.

Adopting a complexity perspective may also better reflect how people encounter the commercial determinants of health. We tackled this large project on an industry-by-industry basis, which influenced our subsequent reporting. However, the consumption of harmful products—such as drinking alcohol and gambling, for example—is often linked. Our public and patient involvement (PPI) panel described how gambling premises and unhealthy food, alcohol and tobacco retailers are clustered in less economically advantaged neighbourhoods, resulting in the layering of risk factors for poor mental health on top of each other, further widening disparities in mental health. Similarly, the mental health impacts go beyond direct consumers; for example, the toll of domestic violence or child abuse triggered by alcohol consumption.

‘Traditional’ systematic review methods—with their interest in linear causal pathways—can struggle to describe the complex, interconnected drivers of health (Petticrew *et al*., 2023). Systems-type approaches have been advocated in the field of the commercial determinants of health in general (Knai *et al*., 2018), including when undertaking evidence synthesis, in part due to their ability to describe the multiple and complicated effects commercial actors can have on health at multiple levels. Building on the research identified here, a future project could seek to develop a systems-type map to aid identification of further leverage points to address the CDOMH and their inequitable impacts. A complex systems approach could also be used to explore the interplay between the physical and mental health problems caused by the commercial determinants to facilitate the development of holistic interventions seeking to improve public health.

### Strengths of review

This umbrella review is the first seeking to pull together interventions addressing the CDOMH. Along with its sister review (Dun-Campbell *et al*., 2024), it provides a valuable resource in (i) highlighting the CDOMH and (ii) serving as a base for future endeavours.

By considering multiple industries, the project is able to compare the differing portfolios of interventions evaluated. For example, it reveals how multiple reviews of product re-design have been undertaken for gambling, whilst more structural interventions have been overlooked. This differs from other industries such as alcohol.

This review is also amongst the few that assess the industry involvement in the identified literature.

Our approach supplemented published funding statements and conflict of interest declarations, with additional checks for key authors. Whilst reliant on information available online and typically provided by the authors themselves, it is an important step in understanding not just the quality of the evidence but also in situating it within paradigms of problem framing and proposed solutions.

### Limitations of the review

Due to time and space constraints, it was not possible to include the literature located regarding fossil fuels and the commercial determinants of our wider living and working conditions, as per our initial protocol. Central to these concerns is the growing body of evidence regarding the multiple pathways by which climate change impacts mental health (Berry *et al*., 2018; Lawrance *et al*., 2022). However, a key barrier in preventing action is the current lack of evidence regarding effective interventions addressing climate change and mental health (Alford *et al*., 2023).

The burden of mental ill health caused by the mortality and morbidity (and associated treatment), resulting from the consumption of unhealthy commodities, was not considered. For example, the leading global risk factors for risk-attributable cancer deaths and disability-adjusted life years are smoking, consuming alcohol and having a high body mass index (GBD Collaborators, 2022). With such large numbers of people and their families affected, better characterization of the associated mental health burden would help provide novel leverage points for public health controls. Furthermore, the review included only four (major) mental health outcomes. When presented with the draft findings, PPI representatives queried why eating disorders had not been included, particularly given our interest in social media. We also did not include mental health outcomes associated with specific industries such as alcohol use disorder and gambling disorder.

The range of industries scrutinized by efforts to better understand—and address—the commercial determinants of the health has diversified since the field’s inception. A relatively recent addition is the firearms industry (Maani *et al.*, 2020a) and, arguably, this industry might have been included in our evidence synthesis. A recent review reported a decrease in firearm-related suicide rates following the introduction of legislation in Canada (Bennett *et al.*, 2022).

Methodological limitations include that, for pragmatic reasons, only articles written in English were eligible for inclusion, and a start date was applied to the search. Furthermore, initial title and abstract screening was undertaken by a single researcher only. We did not consult original study reports and therefore were reliant on the—sometimes poor—quality of reporting of the included reviews.

## CONCLUSION

This umbrella review offers an important first step in addressing the CDOMH. There is some evidence that reducing the availability of alcohol or pesticides may lower suicide rates. By drawing together and mapping the currently very limited evidence base, it begins to counter a frequent collective overlooking of the commercial determinants when seeking to improve population-level mental health. By highlighting gaps in current understanding, it offers a spring board towards the urgently needed high-quality evaluations of commercial determinants interventions developed specifically with the aim of achieving positive mental health outcomes and/or that evaluate mental health outcomes.

## Supplementary Material

daae147_suppl_Supplementary_Files_1

daae147_suppl_Supplementary_Files_2

daae147_suppl_Supplementary_Files_3

daae147_suppl_Supplementary_Files_4

daae147_suppl_Supplementary_Files_5

daae147_suppl_Supplementary_Files_6

## Data Availability

This review synthesizes data from only previously published studies. The Supplementary Files provide details of our screening decisions, quality appraisal findings and summaries of the included study characteristics.

## References

[CIT0001] Abdalla, S. M., Ofei, L., Maani, N. and Galea, S. (2022) Commercial determinants of health in low- and middle-income countries. In Maani, N., Petticrew, M. and Galea, S. (eds), The Commercial Determinants of Health, online edition, Chapter 28. Oxford University Press, Oxford, pp. 283–293.

[CIT0002] Adebiyi, A. O., Uchendu, O. C., Bamgboye, E., Ibitoye, O. and Omotola, B. (2016) Perceived effectiveness of graphic health warnings as a deterrent for smoking initiation among adolescents in selected schools in southwest Nigeria. Tobacco Induced Diseases, 14, 7. 26997939 10.1186/s12971-016-0074-y PMC4797136

[CIT0003] Alford, J., Massazza, A., Jennings, N. R. and Lawrance, E. (2023) Developing global recommendations for action on climate change and mental health across sectors: a Delphi-style study. The Journal of Climate Change and Health, 12, 100252.

[CIT0004] Bareham, B., John, D., Hanratty, B., Kaner, E., O’Donnell, A., Liddle, J. et al (2023) OP95 Co-producing qualitative research and intervention development with peer researchers with lived experience of co-occurring alcohol and mental health problems in old age. Journal of Epidemiology and Community Health, 77, A47.

[CIT0005] Bayer, R. (2008) Stigma and the ethics of public health: not can we but should we. Social Science & Medicine, 67, 463–472. 18502551 10.1016/j.socscimed.2008.03.017

[CIT0006] Bennett, N., Karkada, M., Erdogan, M. and Green, R. S.; Heal-NS Research Program. (2022) The effect of legislation on firearm-related deaths in Canada: a systematic review. CMAJ Open, 10, E500–E507. 10.9778/cmajo.20210192 PMC9177199 35672042

[CIT0007] Berman, M. (2014) Suicide among young Alaska Native men: community risk factors and alcohol control. American Journal of Public Health, 104, S329–S335. 24754505 10.2105/AJPH.2013.301503 PMC4035879

[CIT0008] Berman, M., Hull, T. and May, P. (2000) Alcohol control and injury death in Alaska native communities: wet, damp and dry under Alaska’s local option law. Journal of Studies on Alcohol, 61, 311–319. 10757142 10.15288/jsa.2000.61.311

[CIT0009] Berry, H. L., Waite, T. D., Dear, K. B. G., Capon, A. G. and Murray, V. (2018) The case for systems thinking about climate change and mental health. Nature Climate Change, 8, 282–290.

[CIT0010] Birckmayer, J. and Hemenway, D. (1999) Minimum-age drinking laws and youth suicide, 1970-1990. American Journal of Public Health, 89, 1365–1368. 10474554 10.2105/ajph.89.9.1365 PMC1508754

[CIT0011] BMA. (2018) *Tackling the Causes*: *Promoting Public Mental Health and Investing in Prevention*. British Medical Association, London.

[CIT0012] Chan, J. K. N., Correll, C. U., Wong, C. S. M., Chu, R. S. T., Cheng, S. C., Wong, G. H. S. et al (2023) Life expectancy and years of potential life lost in people with mental disorders: a systematic review and meta-analysis. EClinicalMedicine, 65, 102294. 37965432 10.1016/j.eclinm.2023.102294 PMC10641487

[CIT0013] Collin, J. and Hill, S. E. (2015) Industrial epidemics and inequalities: the commercial sector as a structural driver of inequalities in non-communicable diseases. In Smith, K. E., Bambra, C. and Hill, S. E. (eds), *Health Inequalities*: *Critical Perspectives*, Online edition, Chapter 13. Oxford University Press, Oxford, pp. 177–191.

[CIT0014] Cook, P. J. and Durrance, C. P. (2013) The virtuous tax: lifesaving and crime-prevention effects of the 1991 federal alcohol-tax increase. Journal of Health Economics, 32, 261–267. 23220460 10.1016/j.jhealeco.2012.11.003

[CIT0015] Davies, R. (2022) ‘His anger got bigger’: ministers urged to get serious about gamblers’ deaths. The Guardian, 27 June 2022. https://www.theguardian.com/society/2022/jun/26/gambling-addicts-families-call-regulation-white-paper (last accessed 24 October 2024).

[CIT0016] de Lacy-Vawdon, C. and Livingstone, C. (2020) Defining the commercial determinants of health: a systematic review. BMC Public Health, 20, 1022. 32600398 10.1186/s12889-020-09126-1 PMC7325018

[CIT0017] DHSC. (2021) New Hard-Hitting National TV Ad Urges the Nation to Stay at Home. Department of Health and Social Care, London.

[CIT0018] Drovandi, A., Teague, P. A., Glass, B. and Malau-Aduli, B. (2018) A systematic review of smoker and non-smoker perceptions of visually unappealing cigarette sticks. Tobacco Induced Diseases, 16, 1–11. 10.18332/tid/82191 PMC6659478 31516403

[CIT0019] Dun-Campbell, K., Hartwell, G., Maani, N., Tompson, A., van Schalkwyk, M. C. and Petticrew, M. (2024) Commercial determinants of mental ill health: an umbrella review. PLoS Global Public Health, 4, e0003605. 39196874 10.1371/journal.pgph.0003605 PMC11355563

[CIT0020] Fiskaali, A., Stenbro, A. W., Marcussen, T. and Rask, M. T. (2023) Preventive interventions and harm reduction in online and electronic gambling: a systematic review. Journal of Gambling Studies, 39, 883–911. 35999322 10.1007/s10899-022-10126-6

[CIT0021] Freudenberg, N., Lee, K., Buse, K., Collin, J., Crosbie, E., Friel, S. et al (2021) Defining priorities for action and research on the commercial determinants of health: a conceptual review. American Journal of Public Health, 111, 2202–2211. 34878875 10.2105/AJPH.2021.306491 PMC8667845

[CIT0022] Friedli, L. (2009). Mental Health, Resilience and Inequalities. World Health Organization Regional Office for Europe, Copenhagen.

[CIT0023] GBD 2019 Mental Disorders Collaborators. (2022) Global, regional, and national burden of 12 mental disorders in 204 countries and territories, 1990-2019: a systematic analysis for the Global Burden of Disease Study 2019. Lancet Psychiatry, 9, 137–150. 35026139 10.1016/S2215-0366(21)00395-3 PMC8776563

[CIT0024] GBD Collaborators. (2022) The global burden of cancer attributable to risk factors, 2010-19: a systematic analysis for the Global Burden of Disease Study 2019. Lancet, 400, 563–591. 35988567 10.1016/S0140-6736(22)01438-6 PMC9395583

[CIT0025] Gilmore, A. B., Fabbri, A., Baum, F., Bertscher, A., Bondy, K., Chang, H. J. et al (2023) Defining and conceptualising the commercial determinants of health. Lancet, 401, 1194–1213. 36966782 10.1016/S0140-6736(23)00013-2

[CIT0026] Gornall, J. (2015) Sugar: spinning a web of influence. British Medical Journal, 350, h231. 25673325 10.1136/bmj.h231

[CIT0027] Grucza, R. A., Hipp, P. R., Norberg, K. E., Rundell, L., Evanoff, A., Cavazos-Rehg, P. et al (2012) The legacy of minimum legal drinking age law changes: long-term effects on suicide and homicide deaths among women. Alcohol: Clinical and Experimental Research, 36, 377–384. 10.1111/j.1530-0277.2011.01608.x PMC3587149 22085045

[CIT0028] Gunnell, D., Knipe, D., Chang, S. S., Pearson, M., Konradsen, F., Lee, W. J. et al (2017) Prevention of suicide with regulations aimed at restricting access to highly hazardous pesticides: a systematic review of the international evidence. Lancet Global Health, 5, e1026–e1037. 28807587 10.1016/S2214-109X(17)30299-1

[CIT0029] Hahn, R. A., Middleton, J. C., Elder, R., Brewer, R., Fielding, J., Naimi, T. S. et al (2012) Effects of alcohol retail privatization on excessive alcohol consumption and related harms: a community guide systematic review. American Journal of Preventive Medicine, 42, 418–427. 22424256 10.1016/j.amepre.2012.01.002

[CIT0030] Hingson, R., Merrigan, D. and Heeren, T. (1985) Effects of Massachusetts raising its legal drinking age from 18 to 20 on deaths from teenage homicide, suicide, and nontraffic accidents. Pediatric Clinics of North America, 32, 221–232. 3975091 10.1016/s0031-3955(16)34769-1

[CIT0031] Iacobucci, G. (2023) England plans to raise legal age for buying tobacco each year to create ‘smoke-free’ generation. British Medical Journal, 383, 2297. 37793688 10.1136/bmj.p2297

[CIT0032] Jebb, S. (2015) Yes, I work with the food industry, but I doubt they see me as a friend. The Guardian, 15 February 2015. https://www.theguardian.com/commentisfree/2015/feb/13/advise-food-industry-conflict-of-interest-bmj (last accessed 24 October 2024).

[CIT0033] Jones, N. E., Pieper, C. F. and Robertson, L. S. (1992) The effect of legal drinking age on fatal injuries of adolescents and young adults. American Journal of Public Health, 82, 112–115. 1536313 10.2105/ajph.82.1.112 PMC1694407

[CIT0034] Joubert, C. E. (1994) “Wet” or “dry” county status and its correlates with suicide, homicide, and illegitimacy. Psychology Reports, 74, 296. 10.2466/pr0.1994.74.1.296 8153222

[CIT0035] Kelly, C. and Dale, E. (2011) Ethical perspectives on suicide and suicide prevention. Advances in Psychiatric Treatment, 17, 214–219.

[CIT0036] Kesten, J. M., Bhattacharya, A., Ashiru-Oredope, D., Gobin, M. and Audrey, S. (2017) The Antibiotic Guardian campaign: a qualitative evaluation of an online pledge-based system focused on making better use of antibiotics. BMC Public Health, 18, 5. 28693462 10.1186/s12889-017-4552-9 PMC5504645

[CIT0037] Knai, C., Petticrew, M., Capewell, S., Cassidy, R., Collin, J., Cummins, S. et al (2021) The case for developing a cohesive systems approach to research across unhealthy commodity industries. BMJ Global Health, 6, e003543. 33593757 10.1136/bmjgh-2020-003543 PMC7888371

[CIT0038] Knai, C., Petticrew, M., Mays, N., Capewell, S., Cassidy, R., Cummins, S. et al (2018) Systems thinking as a framework for analyzing commercial determinants of health. Milbank Quarterly, 96, 472–498. 30277610 10.1111/1468-0009.12339 PMC6131339

[CIT0039] Kolves, K., Chitty, K. M., Wardhani, R., Varnik, A., de Leo, D. and Witt, K. (2020) Impact of alcohol policies on suicidal behavior: a systematic literature review. International Journal of Environmental Research and Public Health, 17, 1. 10.3390/ijerph17197030 PMC7578997 32992979

[CIT0040] Kousoulis, A. A. and Goldie, I. (2021) A visualization of a socio-ecological model for urban public mental health approaches. Frontiers of Public Health, 9, 654011. 10.3389/fpubh.2021.654011 PMC8158814 34055718

[CIT0041] Lacy-Nichols, J., Nandi, S., Mialon, M., McCambridge, J., Lee, K., Jones, A. et al (2023) Conceptualising commercial entities in public health: beyond unhealthy commodities and transnational corporations. Lancet, 401, 1214–1228. 36966783 10.1016/S0140-6736(23)00012-0

[CIT0042] Lauber, K., Rutter, H. and Gilmore, A. B. (2021) Big food and the World Health Organization: a qualitative study of industry attempts to influence global-level non-communicable disease policy. BMJ Global Health, 6, e005216. 10.1136/bmjgh-2021-005216 PMC8202098 34117011

[CIT0043] Lawrance, E. L., Thompson, R., Newberry Le Vay, J., Page, L. and Jennings, N. (2022) The impact of climate change on mental health and emotional wellbeing: a narrative review of current evidence, and its implications. International Review of Psychiatry, 34, 443–498. 36165756 10.1080/09540261.2022.2128725

[CIT0044] Lawrence, D., Hancock, K. J. and Kisely, S. (2013) The gap in life expectancy from preventable physical illness in psychiatric patients in Western Australia: retrospective analysis of population based registers. BMJ, 346, f2539. 23694688 10.1136/bmj.f2539 PMC3660620

[CIT0045] Lee, K. and Crosbie, E. (2020) Understanding structure and agency as commercial determinants of health comment on “how neoliberalism is shaping the supply of unhealthy commodities and what this means for NCD prevention”. International Journal of Health Policy and Management, 9, 315–318. 32613804 10.15171/ijhpm.2019.127 PMC7444439

[CIT0046] Lee, K., Freudenberg, N., Zenone, M., Smith, J., Mialon, M., Marten, R. et al (2022) Measuring the commercial determinants of health and disease: a proposed framework. International Journal of Health Services, 52, 115–128. 34723675 10.1177/00207314211044992 PMC8592108

[CIT0047] Lee, N. (1993) Differential deviance and social control mechanisms among two groups of Yup’ik Eskimo. American Indian and Alaska Native Mental Health Research (1987), 5, 57–72. 10.5820/aian.0502.1993.57 8130314

[CIT0048] Li, J., Wang, H., Li, M., Shen, Q., Li, X., Zhang, Y. et al (2020) Effect of alcohol use disorders and alcohol intake on the risk of subsequent depressive symptoms: a systematic review and meta-analysis of cohort studies. Addiction, 115, 1224–1243. 31837230 10.1111/add.14935

[CIT0049] Lund, C., Brooke-Sumner, C., Baingana, F., Baron, E. C., Breuer, E., Chandra, P. et al (2018) Social determinants of mental disorders and the Sustainable Development Goals: a systematic review of reviews. Lancet Psychiatry, 5, 357–369. 29580610 10.1016/S2215-0366(18)30060-9

[CIT0050] Maani, N., Abdalla, S. M. and Galea, S. (2020a) The firearm industry as a commercial determinant of health. American Journal of Public Health, 110, 1182–1183. 32639903 10.2105/AJPH.2020.305788

[CIT0051] Maani, N., Collin, J., Friel, S., Gilmore, A. B., McCambridge, J., Robertson, L. et al (2020b) Bringing the commercial determinants of health out of the shadows: a review of how the commercial determinants are represented in conceptual frameworks. European Journal of Public Health, 30, 660–664. 31953933 10.1093/eurpub/ckz197 PMC7445044

[CIT0052] Maani, N., Robbins, G., Koya, S. F., Babajide, O., Abdalla, S. M. and Galea, S. (2021) Energy, data, and decision-making: a scoping review—the 3D commission. Journal of Urban Health, 98, 79–88. 34374032 10.1007/s11524-021-00563-w PMC8440708

[CIT0053] Maani, N., van Schalkwyk, M. C. I., Filippidis, F. T., Knai, C. and Petticrew, M. (2022) Manufacturing doubt: assessing the effects of independent vs industry-sponsored messaging about the harms of fossil fuels, smoking, alcohol, and sugar sweetened beverages. SSM – Population Health, 17, 101009. 35036514 10.1016/j.ssmph.2021.101009 PMC8749266

[CIT0054] Marcus, J. and Siedler, T. (2015) Reducing binge drinking? The effect of a ban on late-night off-premise alcohol sales on alcohol-related hospital stays in Germany. Journal of Public Economics, 123, 55–77.

[CIT0055] Markowitz, S., Chatterji, P. and Kaestner, R. (2003) Estimating the impact of alcohol policies on youth suicides. Journal of Mental Health Policy and Economics, 6, 37–46. 14578546

[CIT0056] Mialon, M. (2020) An overview of the commercial determinants of health. Global Health, 16, 74. 32807183 10.1186/s12992-020-00607-x PMC7433173

[CIT0057] Mialon, M., Vandevijvere, S., Carriedo-Lutzenkirchen, A., Bero, L., Gomes, F., Petticrew, M. et al (2020) Mechanisms for addressing and managing the influence of corporations on public health policy, research and practice: a scoping review. BMJ Open, 10, e034082. 10.1136/bmjopen-2019-034082 PMC7371213 32690498

[CIT0058] Millar, J. S. (2013) The corporate determinants of health: how big business affects our health, and the need for government action! Canadian Journal of Public Health, 104, e327–e329. 24044474 10.17269/cjph.104.3849 PMC6973691

[CIT0059] Muhunthan, J., Angell, B., Hackett, M. L., Wilson, A., Latimer, J., Eades, A. M. et al (2017) Global systematic review of Indigenous community-led legal interventions to control alcohol. BMJ Open, 7, e013932. 10.1136/bmjopen-2016-013932 PMC5372059 28348189

[CIT0060] Naik, Y., Baker, P., Ismail, S. A., Tillmann, T., Bash, K., Quantz, D. et al (2019) Going upstream - an umbrella review of the macroeconomic determinants of health and health inequalities. BMC Public Health, 19, 1678. 31842835 10.1186/s12889-019-7895-6 PMC6915896

[CIT0061] Nelson, J. P. and McNall, A. D. (2016) Alcohol prices, taxes, and alcohol-related harms: a critical review of natural experiments in alcohol policy for nine countries. Health Policy, 120, 264–272. 26861971 10.1016/j.healthpol.2016.01.018

[CIT0062] Nepal, S., Kypri, K., Tekelab, T., Hodder, R. K., Attia, J., Bagade, T. et al (2020) Effects of extensions and restrictions in alcohol trading hours on the incidence of assault and unintentional injury: systematic review. Journal of Studies on Alcohol and Drugs, 81, 5–23. 32048597

[CIT0063] Nuffield Council on Bioethics. (2007) *Public Health*: *Ethical Issues*. Nuffield Council on Bioethics, London.

[CIT0064] O’Neill, J., Tabish, H., Welch, V., Petticrew, M., Pottie, K., Clarke, M. et al (2014) Applying an equity lens to interventions: using PROGRESS ensures consideration of socially stratifying factors to illuminate inequities in health. Journal of Clinical Epidemiology, 67, 56–64. 24189091 10.1016/j.jclinepi.2013.08.005

[CIT0065] Office of the Surgeon General (OSG). (2023) *Social Media and Youth Mental Health*: *The U.S. Surgeon General’s Advisory*. US Department of Health and Human Services, Washington (DC). 37721985

[CIT0066] Page, M. J., McKenzie, J. E., Bossuyt, P. M., Boutron, I., Hoffmann, T. C., Mulrow, C. D. et al (2021) The PRISMA 2020 statement: an updated guideline for reporting systematic reviews. British Medical Journal, 372, n71. 33782057 10.1136/bmj.n71 PMC8005924

[CIT0067] Papatheodorou, S. (2019) Umbrella reviews: what they are and why we need them. European Journal of Epidemiology, 34, 543–546. 30852716 10.1007/s10654-019-00505-6

[CIT0068] Petticrew, M., Glover, R. E., Volmink, J., Blanchard, L., Cott, E., Knai, C. et al (2023) The Commercial Determinants of Health and Evidence Synthesis (CODES): methodological guidance for systematic reviews and other evidence syntheses. Systematic Reviews, 12, 165. 37710334 10.1186/s13643-023-02323-0 PMC10503085

[CIT0069] PHE. (2021) *Gambling-Related Harms Evidence Review*: *The Economic and Social Cost of Harms*. Public Health England, London.

[CIT0070] PMHIC. (2022) *Public Mental Health Implementation*: *A New Centre and New Opportunities*. Public Mental Health Implementation Centre, the Royal College of Psychiatrists, London.

[CIT0071] Pridemore, W. A., Chamlin, M. B. and Andreev, E. (2013) Reduction in male suicide mortality following the 2006 Russian alcohol policy: an interrupted time series analysis. American Journal of Public Health, 103, 2021–2026. 24028249 10.2105/AJPH.2013.301405 PMC3828708

[CIT0072] Pridemore, W. A. and Snowden, A. J. (2009) Reduction in suicide mortality following a new national alcohol policy in Slovenia: an interrupted time-series analysis. American Journal of Public Health, 99, 915–920. 19299669 10.2105/AJPH.2008.146183 PMC2667837

[CIT0073] Ramstedt, M. (2002) The repeal of medium-strength beer in grocery stores in Sweden – the impact on alcohol-related hospitalizations in different age groups. NAD Publication, 2002, 117–131.

[CIT0074] RCPSYCH. (2019) Alcohol and Depression. Royal College of Psychiatrists, London.

[CIT0075] Regan, M., Smolar, M., Burton, R., Clarke, Z., Sharpe, C., Henn, C. et al (2022) Policies and interventions to reduce harmful gambling: an international Delphi consensus and implementation rating study. Lancet Public Health, 7, e705–e717. 35907421 10.1016/S2468-2667(22)00137-2

[CIT0076] Reifels, L., Mishara, B. L., Dargis, L., Vijayakumar, L., Phillips, M. R. and Pirkis, J. (2019) Outcomes of community-based suicide prevention approaches that involve reducing access to pesticides: a systematic literature review. Suicide and Life-Threatening Behavior, 49, 1019–1031. 30105769 10.1111/sltb.12503

[CIT0077] Rinaldi, C., D’Aguilar, M. and Egan, M. (2022) Understanding the online environment for the delivery of food, alcohol and tobacco: an exploratory analysis of ‘dark kitchens’ and rapid grocery delivery services. International Journal of Environmental Research and Public Health, 19, 5523. 35564918 10.3390/ijerph19095523 PMC9099441

[CIT0078] Rintoul, A., Marionneau, V., Livingstone, C., Nikkinen, J. and Kipsaina, C. (2023) Editorial: gambling, stigma, suicidality, and the internalization of the ‘responsible gambling’ mantra. Frontiers in Psychiatry, 14, 1214531. 37333930 10.3389/fpsyt.2023.1214531 PMC10269197

[CIT0079] Roodbeen, R. T. J., Dijkstra, R. I., Schelleman-Offermans, K., Friele, R. and van de Mheen, D. (2021) Examining the intended and unintended impacts of raising a minimum legal drinking age on primary and secondary societal harm and violence from a contextual policy perspective: a scoping review. International Journal of Environmental Research and Public Health, 18, 1999. 33669507 10.3390/ijerph18041999 PMC7922690

[CIT0080] Savona, N., Macauley, T., Aguiar, A., Banik, A., Boberska, M., Brock, J. et al (2021) Identifying the views of adolescents in five European countries on the drivers of obesity using group model building. European Journal of Public Health, 31, 391–396. 33608719 10.1093/eurpub/ckaa251 PMC8071593

[CIT0103] Serôdio, P. M., McKee, M. and Stuckler, D. (2018) Coca-Cola – a model of transparency in research partnerships? A network analysis of Coca-Cola’s research funding (2008–2016). Public Health Nutrition, 21, 1594–1607. 29560842 10.1017/S136898001700307X PMC5962884

[CIT0082] Sharma, M. K., John, N. and Sahu, M. (2020) Influence of social media on mental health: a systematic review. Current Opinion in Psychiatry, 33, 467–475. 32639362 10.1097/YCO.0000000000000631

[CIT0083] Shea, B. J., Reeves, B. C., Wells, G., Thuku, M., Hamel, C., Moran, J. et al (2017) AMSTAR 2: a critical appraisal tool for systematic reviews that include randomised or non-randomised studies of healthcare interventions, or both. British Medical Journal, 358, j4008. 28935701 10.1136/bmj.j4008 PMC5833365

[CIT0084] Skog, O. J. (1993) Alcohol and suicide in Denmark 1911-24—experiences from a ‘natural experiment’. Addiction, 88, 1189–1193. 8241918 10.1111/j.1360-0443.1993.tb02141.x

[CIT0085] Sloan, F. A., Reilly, B. A. and Schenzler, C. (1994) Effects of prices, civil and criminal sanctions, and law enforcement on alcohol-related mortality. Journal of Studies on Alcohol, 55, 454–465. 7934053 10.15288/jsa.1994.55.454

[CIT0086] Son, C. H. and Topyan, K. (2011) The effect of alcoholic beverage excise tax on alcohol-attributable injury mortalities. European Journal of Health Economics, 12, 103–113. 10.1007/s10198-010-0231-9 20306111

[CIT0087] Stansfield, J., Cavill, N., Marshall, L., Robson, C. and Rutter, H. (2021) Using complex systems mapping to build a strategic public health response to mental health in England. Journal of Public Mental Health, 20, 286–297.

[CIT0088] Steptoe, A. and Frank, P. (2023) Obesity and psychological distress. *Philosophical Transactions of the Royal Society B*: *Biological Sciences*, 378, 20220225. 10.1098/rstb.2022.0225 PMC10475872 37661745

[CIT0089] Stuckler, D., McKee, M., Ebrahim, S. and Basu, S. (2012) Manufacturing epidemics: the role of global producers in increased consumption of unhealthy commodities including processed foods, alcohol, and tobacco. PLoS Medicine, 9, e1001235. 22745605 10.1371/journal.pmed.1001235 PMC3383750

[CIT0090] Taylor, G. M., Lindson, N., Farley, A., Leinberger-Jabari, A., Sawyer, K., Te Water Naude, R. et al (2021) Smoking cessation for improving mental health. Cochrane Database of Systematic Reviews, 3, CD013522. 33687070 10.1002/14651858.CD013522.pub2 PMC8121093

[CIT0091] The Gambling Commission. (2022) Industry Statistics – November 2022. The Gambling Commission, London.

[CIT0092] Torrance, J., John, B., Greville, J., O’Hanrahan, M., Davies, N. and Roderique-Davies, G. (2021) Emergent gambling advertising; a rapid review of marketing content, delivery and structural features. BMC Public Health, 21, 718. 33849493 10.1186/s12889-021-10805-w PMC8043759

[CIT0093] van Schalkwyk, M. C., Maani, N., McKee, M., Thomas, S., Knai, C. and Petticrew, M. (2021a) “When the Fun Stops, Stop”: an analysis of the provenance, framing and evidence of a ‘responsible gambling’ campaign. PLoS One, 16, e0255145. 34437561 10.1371/journal.pone.0255145 PMC8389453

[CIT0094] van Schalkwyk, M. C. I. and Cassidy, R. (2022) The gambling industry: harmful products, predatory practices, and the politics of knowledge. In Maani, N., Petticrew, M. and Galea, S. (eds), The Commercial Determinants of Health, online edition, Chapter 13. Oxford University Press, Oxford, pp. 120–130.

[CIT0095] van Schalkwyk, M. C. I., Collin, J., Eddleston, M., Petticrew, M., Pearson, M., Scholin, L. et al (2023) Conceptualising the commercial determinants of suicide: broadening the lens on suicide and self-harm prevention. Lancet Psychiatry, 10, 363–370. 37019125 10.1016/S2215-0366(23)00043-3

[CIT0096] van Schalkwyk, M. C. I., Petticrew, M., Cassidy, R., Adams, P., McKee, M., Reynolds, J. et al (2021b) A public health approach to gambling regulation: countering powerful influences. Lancet Public Health, 6, e614–e619. 34166631 10.1016/S2468-2667(21)00098-0

[CIT0097] WHO. (2019) Metal Disorders Factsheet. World Health Organization, Geneva.

[CIT0098] WHO. (2022) *World Mental Health Report*: *Transforming Mental Health for All*. World Health Organization, Geneva.

[CIT0099] Wolpert, M. (2018) Rethinking public mental health: learning from obesity. Lancet Psychiatry, 5, 458–460. 29426614 10.1016/S2215-0366(18)30046-4

[CIT0100] Wood, D. S. and Gruenewald, P. J. (2006) Local alcohol prohibition, police presence and serious injury in isolated Alaska Native villages. Addiction, 101, 393–403. 16499512 10.1111/j.1360-0443.2006.01347.x

[CIT0101] Yamasaki, A., Chinami, M., Suzuki, M., Kaneko, Y., Fujita, D. and Shirakawa, T. (2005) Tobacco and alcohol tax relationships with suicide in Switzerland. Psychological Reports, 97, 213–216. 16279328 10.2466/pr0.97.1.213-216

[CIT0102] Zenone, M., Kenworthy, N. and Maani, N. (2022) The social media industry as a commercial determinant of health. International Journal of Health Policy and Management, 12, 6840. 35490262 10.34172/ijhpm.2022.6840 PMC10125226

