# Supplementary material for: Addressing the commercial determinants of mental health: an umbrella review of population-level interventions
Source: Health Promot Int. 2024 Nov 21;39(6):daae147. doi: 10.1093/heapro/daae147 (PMC11579613; doi:10.1093/heapro/daae147)
Supplement: daae147_suppl_Supplementary_Files_2 [file daae147_suppl_supplementary_files_2.docx]

**Supplementary file two: Eligible and ineligible interventions**

| **Eligible ‘upstream’ interventions** |
| --- |
| Product/ Ingredient bans  Temporal limits on purchase  Spatial limits on purchase/ consumption  Age limits on purchase/ consumption  Affordability – prices, excise, taxes  Re-monopolisation of sales  Enforcement & penalties re sales  Marketing limits  Purchase limits  Product Labelling  Warnings at point of choice/ consumption/ purchase  Packaging - plain standardised  Reformulation to make less hazardous (single products, range of products offered)  Changes to consumption/ choice environment  Staff training |
| **Ineligible ‘downstream’ interventions** |
| Clinical services  Educational interventions targeting consumers  Counselling  Personalised/ behavioural feedback  Self-exclusion facilities  Sign posting to counselling/ support services |
