# Supplementary material for: Addressing the commercial determinants of mental health: an umbrella review of population-level interventions
Source: Health Promot Int. 2024 Nov 21;39(6):daae147. doi: 10.1093/heapro/daae147 (PMC11579613; doi:10.1093/heapro/daae147)
Supplement: daae147_suppl_Supplementary_Files_3 [file daae147_suppl_supplementary_files_3.docx]

**Supplementary file three - search strategies**

**Ovid MEDLINE(R)**

| exp Alcohol Drinking/ or exp Alcoholic Beverages/ or exp Smoking/ or exp Tobacco Products/ or exp Electronic Nicotine Delivery Systems/ or Tobacco/ or Nicotine/ or Tobacco Industry/ or Gambling/ or Social media/ or exp Social Networking/ or Fast foods/ or Exp food industry/ or Food-Processing Industry/ or exp Fossil Fuels/ or exp "Oil and Gas Industry"/ or Coal Industry/ or coal mining/ or (alcohol* drink* or alcohol* beverage? or binge drink* or wine or beer or spirits or liquor* or heated tobacco or carbon-heated tobacco or heat-not-burn or tobacco heating system? or tobacco heating device? or tobacco heating product? or tobacco vapor product? or tobacco vapour product? or "heat not burn" or tobacco or smok* or cigarette* or nicotine or e-cig* or vape? or vaping or snuff or snus or betel or quid or gutka or naswar or zarda or maras or toombak or chimo or iqmik or mishri or qimanor or paan or shammah or waterpipe or shisha or gambling or gamble* or betting or wager* or lottery or lotto or lotteries or wager or electronic gambling machine? or social media or Facebook or twitter or Instagram or tik?tok or YouTube or tweet? or whatsapp? or ultra-processed food? or processed food? or junk food? or fast food? or fossil fuel? or oil or non-renewable energy or natural gas or petrol or petroleum or coal or pesticide?).mp. Or ((gaming or fruit or slot) adj machine?).mp. or (social adj2 media).mp. or ((virtual or online) adj2 (communit* or network*)).mp.  or  (((commerce or commercial or corporation? or corporate) adj3 health) or corporate social responsibility).mp.  or (exp Commerce/ or Professional Corporations/ or exp Industry/ or (commerce or commercial or industry or industrial or corporat* or company or companies or business* or firm or firms).mp.) and (exp Marketing/ or Lobbying/ or Conflict of Interest/ or exp Environmental Pollution/ or exp Climate Change/ or Greenhouse Effect/ or (adverti#ing or advertisement or advertisements or advert or adverts or marketing or lobb* or conflict? of interest? or interaction or interactions or influence or influences or activity or activities or tactics or practice or practices or Interference or Corrup* or Strategy or strategies or pollut* or contamina* or climate change or global warming or global heating or greenhouse ga* or sea level?).mp.) |
| --- |
|  |
| exp Public Policy/ or exp Policy/or Legislation/ or Legislation, Food/ or exp "Codes of Ethics"/ or Harm Reduction/ or (Intervention? Or Program* or Policy or policies or Legislat* or Initiative? Or control or controls or Ban? or Framework? or Limit* or Prevention or code? of conduct or Standard or standards or governance).mp. or ((reduc* or mitigati*) adj3 harm?).mp |
| Systematic Review/ or Meta-Analysis/ or (Meta-analysis or systematic review or review of reviews or umbrella review Or Scoping review or mapping review).mp. |
| 1 & 2 & 3 |
| **Limits to human, 2012 to current, in English** |

**Embase Classic+Embase (OVID)**

| exp Alcohol Drinking/ or exp Alcoholic Beverages/ or exp Smoking/ or exp Tobacco Products/ or exp Electronic Nicotine Delivery Systems/ or Tobacco/ or Nicotine/ or Tobacco Industry/ or Gambling/ or Social media/ or exp Social Networking/ or Fast foods/ or Exp food industry/ or Food-Processing Industry/ or exp Fossil Fuels/ or exp "Oil and Gas Industry"/ or Coal Industry/ or coal mining/ or (alcohol* drink* or alcohol* beverage? or binge drink* or wine or beer or spirits or liquor* or heated tobacco or carbon-heated tobacco or heat-not-burn or tobacco heating system? or tobacco heating device? or tobacco heating product? or tobacco vapor product? or tobacco vapour product? or "heat not burn" or tobacco or smok* or cigarette* or nicotine or e-cig* or vape? or vaping or snuff or snus or betel or quid or gutka or naswar or zarda or maras or toombak or chimo or iqmik or mishri or qimanor or paan or shammah or waterpipe or shisha or gambling or gamble* or betting or wager* or lottery or lotto or lotteries or wager or electronic gambling machine? or social media or Facebook or twitter or Instagram or tik?tok or YouTube or tweet? or whatsapp? or ultra-processed food? or processed food? or junk food? or fast food? or fossil fuel? or oil or non-renewable energy or natural gas or petrol or petroleum or coal or pesticide?).mp. Or ((gaming or fruit or slot) adj machine?).mp. or (social adj2 media).mp. or ((virtual or online) adj2 (communit$ or network$)).mp.  or  (((commerce or commercial or corporation? or corporate) adj3 health) or corporate social responsibility).mp.  Or  (exp Commerce/ or Professional Corporations/ or exp Industry/ or (commerce or commercial or industry or industrial or corporat* or company or companies or business* or firm or firms).mp.) and (exp Marketing/ or Lobbying/ or Conflict of Interest/ or exp Environmental Pollution/ or exp Climate Change/ or Greenhouse Effect/ or (adverti#ing or advertisement or advertisements or advert or adverts or marketing or lobb* or conflict? of interest? or interaction or interactions or influence or influences or activity or activities or tactics or practice or practices or Interference or Corrup* or Strategy or strategies or pollut* or contamina* or climate change or global warming or global heating or greenhouse ga* or sea level?).mp.) |
| --- |
|  |
| exp Public Policy/ or exp Policy/or Legislation/ or Legislation, Food/ or exp "Codes of Ethics"/ or Harm Reduction/ or (Intervention? Or Program* or Policy or policies or Legislat* or Initiative? Or Control or controls or Ban? or Framework? or Limit* or Prevention or code? of conduct or Standard or standards or governance).mp. or ((reduc* or mitigati*) adj3 harm?).mp |
| Systematic Review/ or Meta-Analysis/or (Meta-analysis or systematic review or review of reviews or umbrella review Or Scoping review or mapping review).mp. |
| 1 & 2 & 3 |
| **Limits to human, 2012 to current, in English** |

**APA PsycInfo (Ovid)**

| exp Alcoholic Beverages/ or exp Smoking/ or exp Tobacco Products/ or exp Electronic Nicotine Delivery Systems/ or Tobacco/ or Nicotine/ or Tobacco Industry/ or Gambling/ or Social media/ or exp Social Networking/ or Fast foods/ or Exp food industry/ or Food-Processing Industry/ or exp Fossil Fuels/ or exp "Oil and Gas Industry"/ or Coal Industry/ or coal mining/ or (alcohol* drink* or alcohol* beverage? or binge drink* or wine or beer or spirits or liquor* or heated tobacco or carbon-heated tobacco or heat-not-burn or tobacco heating system? or tobacco heating device? or tobacco heating product? or tobacco vapor product? or tobacco vapour product? or "heat not burn" or tobacco or smok* or cigarette* or nicotine or e-cig* or vape? or vaping or snuff or snus or betel or quid or gutka or naswar or zarda or maras or toombak or chimo or iqmik or mishri or qimanor or paan or shammah or waterpipe or shisha or gambling or gamble* or betting or wager* or lottery or lotto or lotteries or wager or electronic gambling machine? or social media or Facebook or twitter or Instagram or tik?tok or YouTube or tweet? or whatsapp? or ultra-processed food? or processed food? or junk food? or fast food? or fossil fuel? or oil or non-renewable energy or natural gas or petrol or petroleum or coal or pesticide?).mp. Or ((gaming or fruit or slot) adj machine?).mp. or (social adj2 media).mp. or ((virtual or online) adj2 (communit$ or network$)).mp.  or  (((commerce or commercial or corporation? or corporate) adj3 health) or corporate social responsibility).mp.  Or  (exp Commerce/ or Professional Corporations/ or exp Industry/ or (commerce or commercial or industry or industrial or corporat* or company or companies or business* or firm or firms).mp.) and (exp Marketing/ or Lobbying/ or Conflict of Interest/ or exp Environmental Pollution/ or exp Climate Change/ or Greenhouse Effect/ or (adverti#ing or advertisement or advertisements or advert or adverts or marketing or lobb* or conflict? of interest? or interaction or interactions or influence or influences or activity or activities or tactics or practice or practices or Interference or Corrup* or Strategy or strategies or pollut* or contamina* or climate change or global warming or global heating or greenhouse ga* or sea level?).mp.) |
| --- |
|  |
| exp Public Policy/ or exp Policy/or Legislation/ or Legislation, Food/ or exp "Codes of Ethics"/ or Harm Reduction/ or (Intervention? Or Program* or Policy or policies or Legislat* or Initiative? Or Control or controls or Ban? or Framework? or Limit* or Prevention or code? of conduct or Standard or standards or governance).mp. or ((reduc* or mitigati*) adj3 harm?).mp |
| Systematic Review/ or Meta-Analysis/or (Meta-analysis or systematic review or review of reviews or umbrella review Or Scoping review or mapping review).mp. |
| 1 & 2 & 3 |
| **Limits to human, 2012 to current, in English** |

**SCOPUS**

| **(("alcoholic drink*" or "alcohol beverage" or "alcoholic beverage" or "binge drink*"or wine or beer or spirits or liquor*) or ("heated tobacco" or "carbon heated tobacco" or "heat not burn" or "tobacco heating system" or "tobacco heating device" or "tobacco heating product" or "tobacco vapor product" or "tobacco vapour product" or tobacco or smok* or cigarette* or nicotine or e-cig* or  vape* or vaping or snuff or snus or betel or quid or gutka or naswar or zarda or maras or  toombak or chimo or iqmik or mishri or qimanor or paan or shammah or waterpipe or shisha) or** (**gambling or gamble* or betting or wager* or lottery or lotto or lotteries or wager or "electronic gambling machine" or (( gaming  OR  fruit  OR  slot )  PRE/1  machine* )) or ({social media} or facebook or twitter or Instagram or tiktok or youtube or tweet* or whatsapp* or (social  W/2  media ) or ((virtual  OR  online)  W/2  ( communit* or network*))) or** (**"ultra processed food" or "processed food" or "junk food" or "fast food") or ("fossil fuel" or oil or "non renewable energy" or "natural gas" or petrol or petroleum or coal or pesticide*))** **]** **OR**  **(((commerce or commercial or corporation* or corporate) W/3 health) or {corporate social responsibility})**  **OR**  **((commerce or commercial or industry or industrial or corporat* or company or companies or business* or firm or firms) and (adverti?ing or advertisement or advertisements or advert or adverts or marketing or lobb* or "conflict of interest" or interaction or interactions or influence or influences or activity or activities or tactics or practice or practices or interference or corrup* or strategy or strategies or** pollut* or contamina* or **{**climate change} or {global warming} or {global heating} or "greenhouse gas*" or "sea level*")) |
| --- |
| And **(intervention* or program* or policy or policies or legislat* or initiative* or control or controls or framework* or limit* or ban or bans or banning or prevention or "code of conduct" or standard or standards or governance or ((reduc* or mitigati*) W/3 harm*))** |
| And **("Meta analysis" or {systematic review} or {review of reviews} or {umbrella review} or {scoping review} or {mapping review})** |
| **Three concepts combined:** **]** |
| **Limits:**  English language |

**Cochrane (Ovid)**

Coal Industry / **Legislation** / Legislation, Food mesh terms excluded as no results.

| MeSH descriptor: [Alcohol Drinking] explode all trees  or MeSH descriptor: [Alcoholic Beverages] explode all trees  or MeSH descriptor: [ Smoking] explode all trees  or MeSH descriptor: [Smoking Devices] explode all trees  or MeSH descriptor: [Tobacco] explode all trees  or MeSH descriptor: [Nicotine] explode all trees  or MeSH descriptor: [Tobacco Industry] explode all trees  or MeSH descriptor: [Gambling] explode all trees  or MeSH descriptor: [Social Networking] explode all trees  or MeSH descriptor: [Fast Foods] explode all trees  or MeSH descriptor: [Food Industry] explode all trees  or MeSH descriptor: [Food-Processing Industry] explode all trees  or MeSH descriptor: [Fossil Fuels] explode all trees  or MeSH descriptor: [Oil and Gas Industry] explode all trees  or “alcohol* drink*” or “alcohol* beverage?” or “binge drink*” or wine or beer or spirits or liquor* or “heat not burn” or “tobacco heating” or “heated tobacco” or “tobacco vapor product?” or “tobacco vapour product?” or "heat not burn" or tobacco or smok* or cigarette* or nicotine or e-cig* or vape? or vaping or snuff or snus or betel or quid or gutka or naswar or zarda or maras or toombak or chimo or iqmik or mishri or qimanor or paan or shammah or waterpipe or shisha or gambling or gamble* or betting or wager* or lottery or lotto or lotteries or wager or “electronic gambling machine?” or “social media” or Facebook or twitter or Instagram or tik?tok or YouTube or tweet? or whatsapp? or “processed food?” or pesticide* or coal or petroleum or petrol or "natural gas" or "non renewable energy" or oil or "fossil fuel?" or ((gaming or fruit or slot) NEAR/1 machine?) or (social NEAR/2 media) or ((virtual or online) NEAR/2 (communit* or network?)) or "junk food?" or "fast food?" |
| --- |
| Or ((commerce or commercial or corporation? or corporate) NEAR/3 health) or “corporate social responsibility” |
| Or  (MeSH descriptor: [Commerce] explode all trees  or MeSH descriptor: [Professional Corporations] explode all trees  or MeSH descriptor: [Industry] explode all trees  or commerce or commercial or industry or industrial or corporat* or company or companies or business* or firm or firms)  **and**  (MeSH descriptor: [Marketing] explode all trees  or MeSH descriptor: [Lobbying] explode all trees  or MeSH descriptor: [Conflict of Interest] explode all trees  or MeSH descriptor: [Environmental Pollution] explode all trees  or MeSH descriptor: [Climate Change] explode all trees  or MeSH descriptor: [Greenhouse Effect] explode all trees  or adverti#ing or advertisement or advertisements or advert or adverts or marketing or lobb* or “conflicts of interests” or "conflict of interest" or interaction or interactions or influence or influences or activity or activities or tactics or practice or practices or Interference or Corrup* or Strategy or strategies or pollut* or contamina* or “climate change” or “global warming” or “global heating” or “greenhouse gas*” or “sea level?”) |
|  |
| MeSH descriptor: [Public Policy] explode all trees  or MeSH descriptor: [Policy] explode all trees  or MeSH descriptor: [Codes of Ethics] explode all trees  or MeSH descriptor: [Harm Reduction] explode all trees  or (Intervention? Or Program* or Policy or policies or Legislat* or Initiative? Or Control or controls or Ban? or Framework? or Limit* or Prevention or “code? of conduct” or Standard or standards or governance) or ((reduc* or mitigati*) NEAR/3 harm?) |
| MeSH descriptor: [Systematic Reviews as Topic] explode all trees  MeSH descriptor: [Meta-Analysis as Topic] explode all trees  or Meta-analysis or systematic review or review of reviews or umbrella review Or Scoping review or mapping review |
| 1 & 2 & 3 |
| **Limits to Cochrane reviews, Cochrane protocols, 2012 to current (July 2022)** |
