# Supplementary material for: Addressing the commercial determinants of mental health: an umbrella review of population-level interventions
Source: Health Promot Int. 2024 Nov 21;39(6):daae147. doi: 10.1093/heapro/daae147 (PMC11579613; doi:10.1093/heapro/daae147)
Supplement: daae147_suppl_Supplementary_Files_4 [file daae147_suppl_supplementary_files_4.docx]

**Supplementary file 4: Studies excluded at full text review stage**

**Social Media**

| **Count** | **Lead Author** | **Year** | **Title** | **Final decision** |
| --- | --- | --- | --- | --- |
| 1 | Rodgers (1) | 2021 | #Take idealized bodies out of the picture: A scoping review of social media content aiming to protect and promote positive body image. | Exclude – not systematic review |
| 2 | Feng (2) | 2020 | Categorizing Online Harassment Interventions | Exclude – not systematic review |
| 3 | Bychkov (3) | 2018 | Facing Up to Nomophobia: A Systematic Review of Mobile Phone Apps that Reduce Smartphone Usage | Exclude – not systematic review |
| 4 | Tiidenberg (4) | 2021 | Sex, power and platform governance | Exclude – not systematic review |
| 5 | Pennycook (5) | 2022 | Accuracy prompts are a replicable and generalizable approach for reducing the spread of misinformation | Exclude – not systematic review |
| 6 | Danthinne (6) | 2020 | Labels to prevent the detrimental effects of media on body image: A systematic review and meta-analysis. | Exclude - wrong intervention |
| 7 | Robinson (7) | 2016 | Social media and suicide prevention: a systematic review. | Exclude - wrong intervention |
| 8 | Bodhi (8) | 2021 | Recent themes in social media research: A systematic review | Exclude - wrong intervention |
| 9 | Throuvala (9) | 2019 | School-based Prevention for Adolescent Internet Addiction: Prevention is the Key. A Systematic Literature Review. | Exclude - wrong intervention |
| 10 | Gaffney (10) | 2019 | Are cyberbullying intervention and prevention programs effective? A systematic and meta-analytical review | Exclude - wrong intervention |
| 11 | Lancaster (11) | 2018 | A Systematic Research Synthesis on Cyberbullying Interventions in the United States. | Exclude - wrong intervention |
| 12 | Ademiluyi (12) | 2022 | Implications and Preventions of Cyberbullying and Social Exclusion in Social Media: Systematic Review | Exclude wrong outcome |
| 13 | Shaikh (13) | 2020 | Cyberbullying: A Systematic Literature Review to Identify the Factors Impelling University Students Towards Cyberbullying | Exclude - wrong outcome |
| 14 | Al-Sarem (14) | 2019 | Deep learning-based rumor detection on microblogging platforms: A systematic review | Exclude wrong outcomes |
| 15 | Almazyad (15) | 2020 | Control vs content: A systematic review of the social media research literature | Exclude wrong outcome |
| 16 | BenSassi (16) | 2021 | Malicious accounts detection from online social networks: a systematic review of literature | Exclude wrong outcome |
| 17 | Mitchell (17) | 2020 | The effect of privacy policies on information sharing behavior on social networks: A systematic literature review | Exclude - wrong outcome |
| 18 | Picardo (18) | 2020 | Suicide and self-harm content on Instagram: A systematic scoping review | Exclude - mechanism not intervention |
| 19 | Marchant (19) | 2021 | Impact of Web-based sharing and viewing of self-harm-related videos and photographs on young people: Systematic review | Exclude - mechanism not intervention |
| 20 | Al-Samarraie (20) | 2021 | Young users' social media addiction: causes, consequences and preventions | Exclude - mechanism not intervention |
| 21 | Barsaiyan (21) | 2021 | Twitter Blue Tick-A Study of its Impact on Society | Exclude - can't access full text |
| 22 | Blaya (22) | 2019 | Cyberhate: A review and content analysis of intervention strategies | Exclude - cites reviews |
| 23 | Charles (23) | 2022 | Typology of content warnings and trigger warnings: Systematic review. | Exclude - cites reviews |
| 24 | Orben (24) | 2020 | Teenagers, screens and social media: a narrative review of reviews and key studies. | Exclude - cites reviews |

**Alcohol**

| **Count** | **Lead Author** | **Year** | **Title** | **Final decision** |
| --- | --- | --- | --- | --- |
| 1 | Baccini (25) | 2014 | Analyzing and comparing the association between control policy measures and alcohol consumption in Europe. | Exclude - not systematic review |
| 2 | Galizzi (26) | 2012 | Label, nudge or tax? A review of health policies for risky behaviours | Exclude - not systematic review |
| 3 | Giesbrecht (27) | 2021 | Alcohol retail privatisation in Canadian provinces between 2012 and 2017. Is decision making oriented to harm reduction? | Exclude - not systematic review |
| 4 | Watterson (28) | 2021 | Workplace intervention programmes for decreasing alcohol use in military personnel: A systematic review | Exclude - wrong intervention |
| 5 | Witt (29) | 2021 | Effect of alcohol interventions on suicidal ideation and behaviour: A systematic review and meta-analysis. | Exclude - wrong intervention |
| 6 | Erng (30) | 2020 | Prevention of Alcohol-Exposed Pregnancies and Fetal Alcohol Spectrum Disorder Among Pregnant and Postpartum Women: A Systematic Review. | Exclude - wrong intervention |
| 7 | Tremblay (31) | 2020 | Primary substance use prevention programs for children and youth: A systematic review | Exclude - wrong intervention |
| 8 | Armstrong-Moore (32) | 2018 | Interventions to reduce the negative effects of alcohol consumption in older adults: a systematic review. | Exclude - wrong intervention |
| 9 | Kelly (33) | 2018 | Interventions to prevent and reduce excessive alcohol consumption in older people: a systematic review and meta-analysis. | Exclude - wrong intervention |
| 10 | Reid (34) | 2021 | Preconception interventions to reduce the risk of alcohol-exposed pregnancies: A systematic review. | Exclude - wrong intervention |
| 11 | Montag (35) | 2012 | A review of evidence-based approaches for reduction of alcohol consumption in Native women who are pregnant or of reproductive age. | Exclude - wrong intervention |
| 12 | Plotnikoff (36) | 2019 | Efficacy of interventions targeting alcohol, drug and smoking behaviors in university and college students: A review of randomized controlled trials. | Exclude - wrong intervention |
| 13 | Yuvaraj (37) | 2019 | Effectiveness of Workplace Intervention for Reducing Alcohol Consumption: a Systematic Review and Meta-Analysis. | Exclude - wrong intervention |
| 14 | Prosser (38) | 2018 | A meta-analysis of effectiveness of E-interventions to reduce alcohol consumption in college and university students. | Exclude - wrong intervention |
| 15 | Riper (39) | 2018 | Effectiveness and treatment moderators of internet interventions for adult problem drinking: An individual patient data meta-analysis of 19 randomised controlled trials. | Exclude - wrong intervention |
| 16 | Scott-Sheldon (40) | 2016 | Alcohol interventions for Greek letter organizations: A systematic review and meta-analysis, 1987 to 2014. | Exclude - wrong intervention |
| 17 | Agabio (41) | 2015 | A systematic review of school-based alcohol and other drug prevention programs | Exclude - wrong intervention |
| 18 | Scott-Sheldon (42) | 2014 | Efficacy of alcohol interventions for first-year college students: a meta-analytic review of randomized controlled trials. | Exclude - wrong intervention |
| 19 | Strom (43) | 2014 | Effectiveness of school-based preventive interventions on adolescent alcohol use: a meta-analysis of randomized controlled trials. | Exclude - wrong intervention |
| 20 | Lee (44) | 2014 | A systematic review of alcohol interventions among workers in male-dominated industries | Exclude - wrong intervention |
| 21 | Foxcroft (45) | 2012 | Universal alcohol misuse prevention programmes for children and adolescents: Cochrane systematic reviews. | Exclude - wrong intervention |
| 22 | Champion (46) | 2016 | Prevention of alcohol and other drug use and related harm in the digital age: what does the evidence tell us?. | Exclude - wrong intervention |
| 23 | Boumans (47) | 2022 | Understanding How and Why Alcohol Interventions Prevent and Reduce Problematic Alcohol Consumption among Older Adults: A Systematic Review | Exclude - wrong intervention |
| 24 | Oh (48) | 2022 | Effectiveness of Digital Interventions for Preventing Alcohol Consumption in Pregnancy: Systematic Review and Meta-analysis. | Exclude - wrong intervention |
| 25 | Ahankari (49) | 2019 | The effectiveness of combined alcohol and sexual risk taking reduction interventions on the sexual behaviour of teenagers and young adults: a systematic review. | Exclude - wrong intervention |
| 26 | McFadyen (50) | 2018 | Strategies to improve the implementation of policies, practices or programmes in sporting organisations targeting poor diet, physical inactivity, obesity, risky alcohol use or tobacco use: a systematic review. | Exclude - wrong intervention |
| 27 | Pena (51) | 2021 | Alcohol policy in Chile: a systematic review of policy developments and evaluations. | Exclude - wrong intervention |
| 28 | Moss (52) | 2018 | The Science of Absent Evidence: Is There Such Thing as an Effective Responsible Drinking Message? | Exclude - wrong intervention |
| 29 | Gray (53) | 2021 | A Scoping Review of "Responsible Drinking" Interventions. | Exclude - wrong intervention |
| 30 | Sherk (54) | 2018 | Alcohol Consumption and the Physical Availability of TakeAway Alcohol: Systematic Reviews and Meta-Analyses of the Days and Hours of Sale and Outlet Density | Exclude - wrong intervention |
| 31 | Noel (55) | 2017 | Does industry self-regulation protect young people from exposure to alcohol marketing? A review of compliance and complaint studies. | Exclude - wrong outcome |
| 32 | Colbert (56) | 2021 | Online alcohol sales and home delivery: an international policy review and systematic literature review | Exclude - wrong outcome |
| 33 | Hollands (57) | 2019 | Altering the availability or proximity of food, alcohol, and tobacco products to change their selection and consumption. | Exclude - wrong outcome |
| 34 | Wettlaufer (58) | 2018 | Can a Label Help me Drink in Moderation? A Review of the Evidence on Standard Drink Labelling. | Exclude - wrong outcome |
| 35 | Noel (59) | 2017 | Industry self-regulation of alcohol marketing: A systematic review of content and exposure research. | Exclude - wrong outcome |
| 36 | Holmes (60) | 2014 | The impact of spatial and temporal availability of alcohol on its consumption and related harms: a critical review in the context of UK licensing policies. | Exclude - wrong outcome |
| 37 | Rehm (61) | 2019 | Regulatory Policies for Alcohol, other Psychoactive Substances and Addictive Behaviours: The Role of Level of Use and Potency. A Systematic Review. | Exclude - wrong outcome |
| 38 | Finan (62) | 2020 | Alcohol Marketing and Adolescent and Young Adult Alcohol Use Behaviors: A Systematic Review of Cross-Sectional Studies. | Exclude - mechanism not intervention |
| 39 | Curtis (63) | 2018 | Meta-Analysis of the Association of Alcohol-Related Social Media Use with Alcohol Consumption and Alcohol-Related Problems in Adolescents and Young Adults. | Exclude - mechanism not intervention |
| 40 | Brown (64) | 2016 | Association Between Alcohol Sports Sponsorship and Consumption: A Systematic Review. | Exclude - mechanism not intervention |
| 41 | Stautz (65) | 2016 | Immediate effects of alcohol marketing communications and media portrayals on consumption and cognition: a systematic review and meta-analysis of experimental studies. | Exclude - mechanism not intervention |
| 42 | Nelson (66) | 2014 | Gender differences in alcohol demand: a systematic review of the role of prices and taxes | Exclude - mechanism not intervention |
| 43 | Bryden (67) | 2012 | A systematic review of the influence on alcohol use of community level availability and marketing of alcohol. | Exclude - mechanism not intervention |
| 44 | Noel (68) | 2020 | Exposure to Digital Alcohol Marketing and Alcohol Use: A Systematic Review | Exclude - mechanism not intervention |
| 45 | Scott (69) | 2017 | Does Industry-Driven Alcohol Marketing Influence Adolescent Drinking Behaviour? A Systematic Review. | Exclude - mechanism not intervention |
| 46 | Khamis (70) | 2022 | Alcohol Consumption Patterns: A Systematic Review of Demographic and Sociocultural Influencing Factors. | Exclude - mechanism not intervention |
| 47 | Herrera Amul (71) | 2020 | Alcohol Advertising, Promotion, and Sponsorship: A Review of Regulatory Policies in the Association of Southeast Asian Nations. | Exclude - can't access full text |
| 48 | Ospina (72) | 2012 | Systematic review on the effectiveness of prevention approaches for fetal alcohol spectrum disorders | Exclude - can't access full text |
| 49 | Clifford (73) | 2021 | A historical overview of legislated alcohol policy in the Northern Territory of Australia: 1979-2021. | Exclude - cites reviews |
| 50 | Anderson (74) | 2021 | Production, Consumption, and Potential Public Health Impact of Low- and No-Alcohol Products: Results of a Scoping Review | Exclude - cites reviews |
| 51 | Sharma (75) | 2017 | Pricing as a means of controlling alcohol consumption | Exclude - cites reviews |
| 52 | Rehm (76) | 2022 | The impact of alcohol taxation changes on unrecorded alcohol consumption: A review and recommendations | Exclude - cites reviews |

**Tobacco**

| **Count** | **Author** | **Year** | **Title** | **Final Decision** |
| --- | --- | --- | --- | --- |
| 1 | Mozaffarian (77) | 2012 | Population approaches to improve diet, physical activity, and smoking habits: a scientific statement from the American Heart Association. | Exclude – not systematic review |
| 2 | Napirah (78) | 2020 | A model of cigarette advertisement policy in preventing children smoking habits in Palu city, Indonesia: A systematic review | Exclude – not systematic review |
| 3 | Rahman (79) | 2014 | Electronic cigarettes: Patterns of use, health effects, use in smoking cessation and regulatory issues | Exclude – not systematic review |
| 4 | Omare (80) | 2022 | A review of tobacco abuse and its epidemiological consequences | Exclude – not systematic review |
| 5 | Coppo (81) | 2014 | School policies for preventing smoking among young people. | Exclude - wrong intervention |
| 6 | Brown (82) | 2015 | Interventions to reduce harm from smoking with families in infancy and early childhood: a systematic review. | Exclude - wrong intervention |
| 7 | O'Connell (83) | 2022 | The effectiveness of smoking cessation interventions for socio-economically disadvantaged women: a systematic review and meta-analysis. | Exclude - wrong intervention |
| 8 | Adibelli (84) | 2020 | Effectiveness of interventions to reduce water pipe smoking behaviors among adolescents: A systematic review | Exclude - wrong intervention |
| 9 | Blaga (85) | 2018 | Use and effectiveness of behavioural economics in interventions for lifestyle risk factors of non-communicable diseases: a systematic review with policy implications. | Exclude - wrong intervention |
| 10 | Carson (86) | 2012 | Interventions for tobacco use prevention in Indigenous youth. | Exclude - wrong intervention |
| 11 | Champion (46) | 2016 | Prevention of alcohol and other drug use and related harm in the digital age: what does the evidence tell us? | Exclude - wrong intervention |
| 12 | Dale (87) | 2014 | The impact of healthy lifestyle interventions on mental health and wellbeing: A systematic review. | Exclude - wrong intervention |
| 13 | Evans-Polce (88) | 2015 | The downside of tobacco control? Smoking and self-stigma: A systematic review. | Exclude - wrong intervention |
| 14 | Huriah (89) | 2020 | School-based smoking prevention in adolescents in developing countries: A literature review | Exclude - wrong intervention |
| 15 | Kader (90) | 2019 | Systematic review of interventions aimed at reducing hookah pipe use: Implications for practitioners and clinicians. | Exclude - wrong intervention |
| 16 | Kohler (91) | 2014 | Smoke-free laws and direct democracy initiatives on smoking bans in Germany: a systematic review and quantitative assessment. | Exclude - wrong intervention |
| 17 | Nethan (92) | 2018 | Smokeless tobacco cessation interventions: A systematic review. | Exclude - wrong intervention |
| 18 | Nishio (93) | 2018 | Systematic review of school tobacco prevention programs in African countries from 2000 to 2016. | Exclude - wrong intervention |
| 19 | Nwosu (94) | 2020 | Reducing Secondhand Smoke Exposure Among Nonsmoking Pregnant Women: A Systematic Review. | Exclude - wrong intervention |
| 20 | Parkinson (95) | 2022 | Exploring Multilevel Workplace Tobacco Control Interventions: A Scoping Review | Exclude - wrong intervention |
| 21 | Peer (96) | 2020 | A narrative systematic review of tobacco cessation interventions in Sub-Saharan Africa | Exclude - wrong intervention |
| 22 | Rosen (97) | 2015 | Effectiveness of Interventions to Reduce Tobacco Smoke Pollution in Homes: A Systematic Review and Meta-Analysis. | Exclude - wrong intervention |
| 23 | Saroj (98) | 2022 | Non-pharmacological interventions for tobacco cessation: A systematic review of existing practices and their effectiveness | Exclude - wrong intervention |
| 24 | Scheffers-van Schayck (99) | 2021 | The Effectiveness of Smoking Cessation Interventions Tailored to Smoking Parents of Children Aged 0-18 Years: A Meta-Analysis. | Exclude - wrong intervention |
| 25 | Song (100) | 2021 | Meta-Analysis of the Effects of Smoking Prevention Programs for Young Adolescents | Exclude - wrong intervention |
| 26 | Soto (101) | 2018 | International Approaches to Tobacco Use Cessation Programming and Prevention Interventions among Indigenous Adolescents and Young Adults | Exclude - wrong intervention |
| 27 | Stanton (102) | 2013 | Tobacco cessation interventions for young people. | Exclude - wrong intervention |
| 28 | Suls (103) | 2012 | Efficacy of smoking-cessation interventions for young adults: a meta-analysis. | Exclude - wrong intervention |
| 29 | Taylor (104) | 2017 | Internet-based interventions for smoking cessation. | Exclude - wrong intervention |
| 30 | Thomas (105) | 2013 | School-based programmes for preventing smoking. | Exclude - wrong intervention |
| 31 | Vance (106) | 2022 | The effectiveness of smoking cessation interventions in rural and remote populations: Systematic review and meta-analyses | Exclude - wrong intervention |
| 32 | Villanti (107) | 2020 | Smoking-Cessation Interventions for U.S. Young Adults: Updated Systematic Review. | Exclude - wrong intervention |
| 33 | Washio (108) | 2014 | Systematic Review of Interventions for Racial/Ethnic-Minority Pregnant Smokers | Exclude - wrong intervention |
| 34 | Behbod (109) | 2018 | Family and carer smoking control programmes for reducing children's exposure to environmental tobacco smoke | Exclude - wrong intervention |
| 35 | Cahill (110) | 2014 | Workplace interventions for smoking cessation | Exclude - wrong intervention |
| 36 | Stevenson (111) | 2017 | Establishing Smoke-Free Homes in the Indigenous Populations of Australia, New Zealand, Canada and the United States: A Systematic Literature Review. | Exclude - wrong intervention |
| 37 | Monson (112) | 2017 | Effects of enactment of legislative (public) smoking bans on voluntary home smoking restrictions: A review | Exclude - wrong outcome |
| 38 | Allen (113) | 2018 | Evaluation of research on interventions aligned to WHO 'Best Buys' for NCDs in low-income and lower-middle-income countries: A systematic review from 1990 to 2015 | Exclude - wrong outcome |
| 39 | Collins (114) | 2019 | E-Cigarette Marketing and Communication: How E-Cigarette Companies Market E-Cigarettes and the Public Engages with E-cigarette Information. | Exclude - wrong outcome |
| 40 | Drovandi (115) | 2018 | A systematic review of smoker and non-smoker perceptions of visually unappealing cigarette sticks | Exclude - wrong outcome |
| 41 | Francis (116) | 2019 | Impact of tobacco-pack pictorial warnings on youth and young adults: A systematic review of experimental studies | Exclude - wrong outcome |
| 42 | Gardner (117) | 2018 | A scoping review of the evidence on health promotion interventions for reducing waterpipe smoking: Implications for practice | Exclude - wrong outcome |
| 43 | Guindon (118) | 2015 | The impact of prices and taxes on the use of tobacco products in Latin America and the Caribbean | Exclude - wrong outcome |
| 44 | Hughes (119) | 2016 | Perceptions and impact of plain packaging of tobacco products in low and middle income countries, middle to upper income countries and low-income settings in high-income countries: a systematic review of the literature. | Exclude - wrong outcome |
| 45 | Iqbal (120) | 2022 | Smoking cessation interventions in South Asian Region: a systematic scoping review. | Exclude - wrong outcome |
| 46 | Janmohamed (121) | 2022 | Interventions to Mitigate Vaping Misinformation: A Meta-Analysis. | Exclude - wrong outcome |
| 47 | Jo (122) | 2017 | Child-resistant and tamper-resistant packaging: A systematic review to inform tobacco packaging regulation. | Exclude - wrong outcome |
| 48 | Johnson (123) | 2021 | Responses to reduced nicotine cigarette marketing features: a systematic review | Exclude - wrong outcome |
| 49 | Lee (124) | 2016 | "May I Buy a Pack of Marlboros, Please?" A Systematic Review of Evidence to Improve the Validity and Impact of Youth Undercover Buy Inspections. | Exclude - wrong outcome |
| 50 | Moodie (125) | 2021 | Consumer response to standardized tobacco packaging in the United Kingdom: A synthesis of evidence from two systematic reviews | Exclude - wrong outcome |
| 51 | Noar (126) | 2017 | Effects of Strengthening Cigarette Pack Warnings on Attention and Message Processing: A Systematic Review | Exclude - wrong outcome |
| 52 | Noar (127) | 2016 | Pictorial cigarette pack warnings: a meta-analysis of experimental studies. | Exclude - wrong outcome |
| 53 | Padhiary (128) | 2021 | A systematic review report on tobacco products and its health issues in India. | Exclude - wrong outcome |
| 54 | Pang (129) | 2021 | The effectiveness of graphic health warnings on tobacco products: a systematic review on perceived harm and quit intentions. | Exclude - wrong outcome |
| 55 | Papanastasiou (130) | 2019 | Evidence From Qualitative Studies of Youth About the Impacts of Tobacco Control Policy on Young People in Europe: A Systematic Review. | Exclude - wrong outcome |
| 56 | Rando-Matos (131) | 2017 | Smokefree legislation effects on respiratory and sensory disorders: A systematic review and meta-analysis. | Exclude - wrong outcome |
| 57 | Stead (132) | 2013 | Is consumer response to plain/standardised tobacco packaging consistent with framework convention on tobacco control guidelines? A systematic review of quantitative studies. | Exclude - wrong outcome |
| 58 | Tee (133) | 2015 | Systematic review on international practices in controlling waterpipe tobacco smoking. | Exclude - wrong outcome |
| 59 | Munthali (134) | 2021 | Assessment of tobacco control policy instruments, status and effectiveness in Africa: A systematic literature review | Exclude - wrong outcome |
| 60 | Tremblay (135) | 2015 | Regulation profiles of e-cigarettes in the United States: A critical review with qualitative synthesis | Exclude - wrong outcome |
| 61 | Vilhelmsson (136) | 2018 | Reducing health inequalities with interventions targeting behavioral factors among individuals with low levels of education - A rapid review. | Exclude - wrong outcome |
| 62 | Sheikh (137) | 2021 | Tobacco industry pricing strategies in response to excise tax policies: a systematic review | Exclude - wrong outcome |
| 63 | Golden (138) | 2016 | Beyond excise taxes: a systematic review of literature on non-tax policy approaches to raising tobacco product prices. | Exclude - wrong outcome |
| 64 | Babaie (139) | 2021 | Preventing and controlling water pipe smoking: a systematic review of management interventions | Exclude - Mechanism not intervention |
| 65 | Grilo (140) | 2021 | A scoping review on disparities in exposure to advertising for e-cigarettes and heated tobacco products and implications for advancing a health equity research agenda. | Exclude - Mechanism not intervention |
| 66 | Kyriakos (141) | 2021 | Flavour capsule cigarette use and perceptions: a systematic review | Exclude - Mechanism not intervention |
| 67 | Lee (142) | 2018 | A new form of nicotine retailers: a systematic review of the sales and marketing practices of vape shops | Exclude - Mechanism not intervention |
| 68 | McCausland (143) | 2019 | The Messages Presented in Electronic Cigarette-Related Social Media Promotions and Discussion: Scoping Review. | Exclude - Mechanism not intervention |
| 69 | Robertson (144) | 2016 | Point-of-sale tobacco promotion and youth smoking: a meta-analysis. | Exclude - Mechanism not intervention |
| 70 | Lee (145) | 2012 | The vector of the tobacco epidemic: tobacco industry practices in low and middle-income countries. | Exclude - Mechanism not intervention |
| 71 | Amul (146) | 2021 | A Systematic Review of Tobacco Industry Tactics in Southeast Asia: Lessons for Other Low- And Middle Income Regions. | Exclude - Mechanism not intervention |
| 72 | Banks (147) | 2017 | Factors influencing the tobacco control policy process in Egypt and Iran: a scoping review | Exclude - Mechanism not intervention |
| 73 | Finan (62) | 2019 | Tobacco outlet density and adolescents' cigarette smoking: a meta-analysis. | Exclude - Mechanism not intervention |
| 74 | Krishnamoorthy (148) | 2020 | Impact of tobacco industry pricing and marketing strategy on brand choice, loyalty and cessation in global south countries: a systematic review. | Exclude - Mechanism not intervention |
| 75 | Lee (149) | 2021 | Associations of tobacco retailer density and proximity with adult tobacco use behaviours and health outcomes: a meta-analysis | Exclude - Mechanism not intervention |
| 76 | Marsh (150) | 2021 | Association between density and proximity of tobacco retail outlets with smoking: A systematic review of youth studies. | Exclude - Mechanism not intervention |
| 77 | Nuyts (151) | 2021 | The Association between Tobacco Outlet Density and Smoking among Young People: A Systematic Methodological Review | Exclude - Mechanism not intervention |
| 78 | Reimold (152) | 2022 | Tobacco company agreements with tobacco retailers for price discounts and prime placement of products and advertising: a scoping review | Exclude - Mechanism not intervention |
| 79 | Savell (153) | 2014 | How does the tobacco industry attempt to influence marketing regulations? A systematic review. | Exclude - Mechanism not intervention |
| 80 | Smith (154) | 2013 | What is known about tobacco industry efforts to influence tobacco tax? A systematic review of empirical studies. | Exclude - Mechanism not intervention |
| 81 | Travis (155) | 2021 | Tobacco retail availability and cigarette and e-cigarette use among youth and adults: a scoping review | Exclude - Mechanism not intervention |
| 82 | Villanti (156) | 2017 | Menthol cigarettes and the public health standard: a systematic review. | Exclude - Mechanism not intervention |
| 83 | Wynne (157) | 2018 | Signs, Fines and Compliance Officers: A Systematic Review of Strategies for Enforcing Smoke-Free Policy. | Exclude - Mechanism not intervention |
| 84 | Liber (158) | 2022 | Tobacco Couponing: A Systematic Review of Exposures and Effects on Tobacco Initiation and Cessation | Exclude - can't access full text |
| 85 | Napirah (159) | 2021 | Implementing a Non-Smoking Regional Policy to Prohibit Childrens' Smoking Habits In Palu City, Indonesia: A Systematic Review | Exclude - can't access full text |
| 86 | Rosario (160) | 2021 | Tobacco use among native Hawaiian and Pacific Islander youth in the U.S. and USAPI: a systematic review of the literature | Exclude - can't access full text |
| 87 | Xiao (161) | 2015 | Implementation of the World Health Organization Framework Convention on Tobacco Control in China: An arduous and long-term task | Exclude - cites reviews |
| 88 | Chamberlain (162) | 2017 | Evidence for a comprehensive approach to Aboriginal tobacco control to maintain the decline in smoking: an overview of reviews among Indigenous peoples. | Exclude - cites reviews |
| 89 | Glasser (163) | 2021 | Retailer density reduction approaches to tobacco control: A review | Exclude - cites reviews |
| 90 | Haddad (164) | 2015 | Waterpipe smoking and regulation in the United States: A comprehensive review of the literature | Exclude - cites reviews |
| 91 | Hill (165) | 2014 | Impact of tobacco control interventions on socioeconomic inequalities in smoking: review of the evidence | Exclude - cites reviews |
| 92 | Livingstone-Banks (166) | 2022 | Effects of interventions to combat tobacco addiction: Cochrane update of 2019 and 2020 reviews. | Exclude - cites reviews |
| 93 | Mannocci (167) | 2019 | What public health strategies work to reduce the tobacco demand among young people? An umbrella review of systematic reviews and meta-analyses. | Exclude - cites reviews |
| 94 | O'Connell (168) | 2022 | Local and State Policy Action Taken in the United States to Address the Emergence of E-Cigarettes and Vaping: A Scoping Review of Literature. | Exclude - cites reviews |
| 95 | Owotomo (169) | 2014 | Current Trends and Impact of Smoking Cessation Interventions for Adult Smokers in Low and Middle Income Countries: A Systematic Literature Review | Exclude - cites reviews |
| 96 | Palmer (170) | 2022 | Urgent Need for Novel Investigations of Treatments to Quit E-cigarettes: Findings from a Systematic Review | Exclude - cites reviews |
| 97 | Pratiti (171) | 2019 | Epidemiology and Adverse Consequences of Hookah/Waterpipe Use: A Systematic Review. | Exclude - cites reviews |
| 98 | Puljevic (172) | 2022 | Closing the gaps in tobacco endgame evidence: a scoping review. | Exclude - cites reviews |
| 99 | South (173) | 2022 | Reducing lifestyle risk behaviours in disadvantaged groups in high-income countries: A scoping review of systematic reviews. | Exclude - cites reviews |
| 100 | Struik (174) | 2020 | Tactics for Drawing Youth to Vaping: Content Analysis of Electronic Cigarette Advertisements. | Exclude - cites reviews |
| 101 | van der Eijk (175) | 2021 | E-Cigarette Markets and Policy Responses in Southeast Asia: A Scoping Review | Exclude - cites reviews |
| 102 | Wolfenden (176) | 2014 | Translation of tobacco control programs in schools: findings from a rapid review of systematic reviews of implementation and dissemination interventions. | Exclude - cites reviews |
| 103 | Lee (177) | 2014 | Promotion of tobacco use cessation for lesbian, gay, bisexual, and transgender people: a systematic review. | Exclude - wrong population |

**Gambling**

| **Count** | **Lead Author** | **Year** | **Title** | **Final decision** |
| --- | --- | --- | --- | --- |
| 1 | Gainsbury (178) | 2014 | Recommendations for international gambling harm-minimisation guidelines: Comparison with effective public health policy. | Exclude - not a systematic review |
| 2 | Gainsbury (179) | 2014 | Review of self-exclusion from gambling venues as an intervention for problem gambling | Exclude - not a systematic review |
| 3 | Shi (180) | 2020 | A Perspective on Age Restrictions and Other Harm Reduction Approaches Targeting Youth Online Gambling, Considering Convergences of Gambling and Videogaming | Exclude - not a systematic review |
| 4 | Yu (181) | 2019 | Youth Gambling in Hong Kong: Prevalence, Psychosocial Correlates, and Prevention | Exclude - wrong intervention |
| 5 | Lozano (182) | 2022 | Systematic Review: Preventive Intervention to Curb the Youth Online Gambling Problem | Exclude - wrong intervention |
| 6 | Hahmann (183) | 2021 | Problem gambling within the context of poverty: a scoping review | Exclude - wrong intervention |
| 7 | Marchica (184) | 2016 | Examining personalized feedback interventions for gambling disorders: A systematic review | Exclude wrong intervention |
| 8 | Kotter (185) | 2019 | A Systematic Review of Land-Based Self-Exclusion Programs: Demographics, Gambling Behavior, Gambling Problems, Mental Symptoms, and Mental Health. | Exclude - wrong intervention |
| 9 | Matheson (186) | 2019 | The use of self-management strategies for problem gambling: a scoping review. | Exclude - wrong intervention |
| 10 | Ladouceur (187) | 2012 | Pre-commitment in gambling: a review of the empirical evidence | Exclude - wrong intervention |
| 11 | Paterson (188) | 2021 | An overview of digital and online strategies to reduce gambling harm. | Exclude - wrong intervention |
| 12 | Grande-Gosende (189) | 2020 | Systematic Review of Preventive Programs for Reducing Problem Gambling Behaviors Among Young Adults. | Exclude - wrong intervention |
| 13 | Akcayir (190) | 2022 | Whose Responsibility Is It to Prevent or Reduce Gambling Harm? A Mapping Review of Current Empirical Research | Exclude - wrong outcome |
| 14 | Raybould (191) | 2021 | Is there a health inequality in gambling related harms? A systematic review. | Exclude – mechanism not intervention |
| 15 | Akcayir(192) | 2022 | Emerging Gambling Problems and Suggested Interventions: A Systematic Review of Empirical Research | Exclude - can't access full text |
| 16 | Skarupova (193) | 2020 | Early intervention and identification of gambling disorder: a systematic literature review of strategies implemented by gambling operators. | Exclude - cites reviews |
| 17 | Ginley (194) | 2017 | Warning messages for electronic gambling machines: Evidence for regulatory policies. | Exclude - cites reviews |
| 18 | Ariyabuddhiphongs (195) | 2013 | Problem Gambling Prevention: Before, During, and After Measures | Exclude - cites reviews |
| 19 | Williams (196) | 2012 | Prevention of Problem Gambling: A Comprehensive Review of the Evidence and Identified Best Practices; | Exclude - cites reviews |

**Pesticides**

| **Count** | **Author** | **Year** | **Title** | **Final decision** |
| --- | --- | --- | --- | --- |
| 1 | Chen (197) | 2016 | Suicide Prevention Through Restricting Access to Suicide Means and Hotspots | Exclude – not systematic review |
| 2 | Panuwet (198) | 2012 | Agricultural pesticide management in Thailand: Status and population health risk | Exclude – not systematic review |
| 3 | Varghese (199) | 2022 | Pesticide Poisoning Among Children in India: The Need for an Urgent Solution | Exclude – not systematic review |
| 4 | Afshari (200) | 2021 | Effectiveness of interventions to promote pesticide safety and reduce pesticide exposure in agricultural health studies: A systematic review. | Exclude - wrong intervention |
| 5 | Fuhrimann (201) | 2022 | Pesticide research on environmental and human exposure and risks in sub-Saharan Africa: A systematic literature review | Exclude – mechanism rather than intervention |
| 6 | Lopes-Ferreira (202) | 2022 | Impact of Pesticides on Human Health in the Last Six Years in Brazil | Exclude – mechanism rather than intervention |
| 7 | Haby (203) | 2016 | Interventions that facilitate sustainable development by preventing toxic exposure to chemicals: an overview of systematic reviews. | Exclude - cites reviews |

**Ultra-Processed Foods**

| **Count** | **Author** | **Year** | **Title** | **Final decision** |
| --- | --- | --- | --- | --- |
| 1 | Bowen (204) | 2015 | Identifying the effects of environmental and policy change interventions on healthy eating | Exclude – not systematic review |
| 2 | Vandevijvere (205) | 2019 | Effect of Formulation, Labelling, and Taxation Policies on the Nutritional Quality of the Food Supply | Exclude – not systematic review |
| 3 | Watt (206) | 2020 | Reducing consumption of unhealthy foods and beverages through banning price promotions: what is the evidence and will it work? | Exclude – not systematic review |
| 4 | Ikonen (207) | 2020 | Consumer effects of front-of-package nutrition labeling: An interdisciplinary meta-analysis. | Exclude – not systematic review |
| 5 | Temple (208) | 2020 | Front-of-package food labels: A narrative review | Exclude – not systematic review |
| 6 | Nikolaou (209) | 2015 | Calorie-labelling: does it impact on calorie purchase in catering outlets and the views of young adults? | Exclude – not systematic review |
| 7 | Hawley (210) | 2013 | The science on front-of-package food labels | Exclude – not systematic review |
| 8 | Trude (211) | 2022 | An equity-oriented systematic review of online grocery shopping among low-income populations: implications for policy and research. | Exclude - wrong intervention |
| 9 | Wolfenden (212) | 2017 | Strategies for enhancing the implementation of school-based policies or practices targeting risk factors for chronic disease | Exclude - wrong intervention |
| 10 | Balhareth (213) | 2019 | Overweight and obesity among adults in the Gulf States: A systematic literature review of correlates of weight, weight-related behaviours, and interventions | Exclude - wrong intervention |
| 11 | Grieger (214) | 2016 | Discrete strategies to reduce intake of discretionary food choices: A scoping review | Exclude - wrong intervention |
| 12 | Robinson (215) | 2014 | What everyone else is eating: a systematic review and meta-analysis of the effect of informational eating norms on eating behavior. | Exclude - wrong intervention |
| 13 | Sirasa (216) | 2019 | Family and community factors shaping the eating behaviour of preschool-aged children in low and middle-income countries: A systematic review of interventions. | Exclude - wrong intervention |
| 14 | Wolfenden (217) | 2014 | A systematic review and meta-analysis of whole of community interventions to prevent excessive population weight gain | Exclude - wrong intervention |
| 15 | Wolfenden (212) | 2020 | Strategies to improve the implementation of healthy eating, physical activity and obesity prevention policies, practices or programmes within childcare services | Exclude - wrong intervention |
| 16 | Boelsen-Robinson (218) | 2015 | A systematic review of the effectiveness of whole-of-community interventions by socioeconomic position | Exclude - wrong intervention |
| 17 | Whittemore (219) | 2013 | School-based internet obesity prevention programs for adolescents: A systematic literature review | Exclude - wrong intervention |
| 18 | Saraf (220) | 2012 | A systematic review of school-based interventions to prevent risk factors associated with noncommunicable diseases. | Exclude - wrong intervention |
| 19 | Rachman (221) | 2022 | The effectiveness of nutrition and health intervention in workplace setting: A systematic review | Exclude - wrong intervention |
| 20 | Sawada (222) | 2019 | Social marketing including financial incentive programs at worksite cafeterias for preventing obesity: a systematic review. | Exclude - wrong intervention |
| 21 | Tam (223) | 2018 | A systematic review of the long-term effectiveness of work-based lifestyle interventions to tackle overweight and obesity | Exclude - wrong intervention |
| 22 | Geaney (224) | 2013 | The effectiveness of workplace dietary modification interventions: a systematic review. | Exclude - wrong intervention |
| 23 | Aceves-Martins (225) | 2022 | Interventions to Prevent Obesity in Mexican Children and Adolescents: Systematic Review | Exclude - wrong intervention |
| 24 | Bagnall (226) | 2019 | Whole systems approaches to obesity and other complex public health challenges: a systematic review. | Exclude - wrong intervention |
| 25 | Hillier-Brown (227) | 2014 | A systematic review of the effectiveness of individual, community and societal-level interventions at reducing socio-economic inequalities in obesity among adults. | Exclude - wrong intervention |
| 26 | Macmillan (228) | 2018 | Do natural experiments of changes in neighborhood built environment impact physical activity and diet? A systematic review | Exclude - wrong intervention |
| 27 | Abeykoon (229) | 2017 | Health-related outcomes of new grocery store interventions: a systematic review. | Exclude - wrong intervention |
| 28 | Escaron (230) | 2013 | Supermarket and grocery store-based interventions to promote healthful food choices and eating practices: a systematic review. | Exclude - wrong intervention |
| 29 | Hartmann-Boyce (231) | 2018 | Grocery store interventions to change food purchasing behaviors: a systematic review of randomized controlled trials. | Exclude - wrong intervention |
| 30 | Kraak (232) | 2019 | Progress Evaluation for Transnational Restaurant Chains to Reformulate Products and Standardize Portions to Meet Healthy Dietary Guidelines and Reduce Obesity and Non-Communicable Disease Risks, 2000-2018: A Scoping and Systematic Review to Inform Policy. | Exclude - wrong outcome |
| 31 | Colón-Ramos (233) | 2014 | Impact of WHO recommendations to eliminate industrial trans-fatty acids from the food supply in Latin America and the Caribbean | Exclude - wrong outcome |
| 32 | Elliott (234) | 2022 | Health Taxes on Tobacco, Alcohol, Food and Drinks in Low- and Middle-Income Countries: A Scoping Review of Policy Content, Actors, Process and Context. | Exclude - wrong outcome |
| 33 | Eyles (235) | 2012 | Food pricing strategies, population diets, and non-communicable disease: a systematic review of simulation studies. | Exclude - wrong outcome |
| 34 | Kenny (236) | 2020 | The Retail Food Sector and Indigenous Peoples in High-Income Countries: A Systematic Scoping Review. | Exclude - wrong outcome |
| 35 | Blake (237) | 2019 | Investigating business outcomes of healthy food retail strategies: A systematic scoping review. | Exclude - wrong outcome |
| 36 | Adam (238) | 2016 | What is the effectiveness of obesity related interventions at retail grocery stores and supermarkets? -a systematic review. | Exclude - wrong outcome |
| 37 | Fuster (239) | 2021 | Facilitating healthier eating at restaurants: A multidisciplinary scoping review comparing strategies, barriers, motivators, and outcomes by restaurant type and initiator | Exclude - wrong outcome |
| 38 | Valdivia Espino (240) | 2015 | Community-based restaurant interventions to promote healthy eating: a systematic review. | Exclude - wrong outcome |
| 39 | Thorpe (241) | 2021 | Business outcomes of healthy food service initiatives in schools: A systematic review. | Exclude - wrong outcome |
| 40 | Marcano-Olivier (242) | 2020 | Using Nudges to Promote Healthy Food Choices in the School Dining Room: A Systematic Review of Previous Investigations. | Exclude - wrong outcome |
| 41 | Gordon (243) | 2018 | Healthier Choices in School Cafeterias: A Systematic Review of Cafeteria Interventions. | Exclude - wrong outcome |
| 42 | Barbour (244) | 2022 | Local urban government policies to facilitate healthy and environmentally sustainable diet-related practices: a scoping review. | Exclude - wrong outcome |
| 43 | Luongo (245) | 2020 | The Retail Food Environment, Store Foods, and Diet and Health among Indigenous Populations: a Scoping Review. | Exclude - wrong outcome |
| 44 | Chung (246) | 2022 | Policies to restrict unhealthy food and beverage advertising in outdoor spaces and on publicly owned assets: A scoping review of the literature. | Exclude - wrong outcome |
| 45 | Smithers (247) | 2014 | Industry self-regulation and TV advertising of foods to Australian children. | Exclude - wrong outcome |
| 46 | Galbraith-Emami (248) | 2013 | The impact of initiatives to limit the advertising of food and beverage products to children: a systematic review. | Exclude - wrong outcome |
| 47 | Gynell (249) | 2022 | The effectiveness of implicit interventions in food menus to promote healthier eating behaviours: A systematic review. | Exclude - wrong outcome |
| 48 | Benajiba (250) | 2020 | Food Labeling Use by Consumers in Arab countries: A Scoping Review | Exclude - wrong outcome |
| 49 | Feteira-Santos (251) | 2020 | Effectiveness of interpretive front-of-pack nutritional labelling schemes on the promotion of healthier food choices: a systematic review. | Exclude - wrong outcome |
| 50 | Rincon-Gallardo (252) | 2020 | Effects of Menu Labeling Policies on Transnational Restaurant Chains to Promote a Healthy Diet: A Scoping Review to Inform Policy and Research. | Exclude - wrong outcome |
| 51 | Oostenbach (253) | 2019 | Systematic review of the impact of nutrition claims related to fat, sugar and energy content on food choices and energy intake. | Exclude - wrong outcome |
| 52 | Blaga (85) | 2018 | Use and effectiveness of behavioural economics in interventions for lifestyle risk factors of non-communicable diseases: a systematic review with policy implications. | Exclude - wrong outcome |
| 53 | Bergallo (254) | 2018 | Regulatory initiatives to reduce sugar-sweetened beverages (SSBs) in Latin America. | Exclude - wrong outcome |
| 54 | Needham (255) | 2020 | A systematic review of the Australian food retail environment: Characteristics, variation by geographic area, socioeconomic position and associations with diet and obesity. | Exclude - mechanism not intervention |
| 55 | Mackenbach (256) | 2019 | A Systematic Review on Socioeconomic Differences in the Association between the Food Environment and Dietary Behaviors. | Exclude - mechanism not intervention |
| 56 | Bennett (257) | 2020 | Prevalence of healthy and unhealthy food and beverage price promotions and their potential influence on shopper purchasing behaviour: A systematic review of the literature | Exclude - mechanism not intervention |
| 57 | Maganja (258) | 2022 | Evidence Gaps in Assessments of the Healthiness of Online Supermarkets Highlight the Need for New Monitoring Tools: a Systematic Review. | Exclude - mechanism not intervention |
| 58 | Xin (259) | 2021 | Association between access to convenience stores and childhood obesity: A systematic review | Exclude - mechanism not intervention |
| 59 | Stevenson (260) | 2019 | Neighbourhood retail food outlet access, diet and body mass index in Canada: a systematic review. | Exclude - mechanism not intervention |
| 60 | Turbutt (261) | 2019 | The impact of hot food takeaways near schools in the UK on childhood obesity: a systematic review of the evidence. | Exclude - mechanism not intervention |
| 61 | Cobb (262) | 2015 | The relationship of the local food environment with obesity: A systematic review of methods, study quality, and results. | Exclude - mechanism not intervention |
| 62 | Williams (263) | 2014 | A systematic review of the influence of the retail food environment around schools on obesity-related outcomes. | Exclude - mechanism not intervention |
| 63 | McCarthy (264) | 2022 | The influence of unhealthy food and beverage marketing through social media and advergaming on diet-related outcomes in children-A systematic review. | Exclude - mechanism not intervention |
| 64 | Packer (265) | 2022 | The impact on dietary outcomes of licensed and brand equity characters in marketing unhealthy foods to children: A systematic review and meta-analysis. | Exclude - mechanism not intervention |
| 65 | Packer (266) | 2022 | The Impact on Dietary Outcomes of Celebrities and Influencers in Marketing Unhealthy Foods to Children: A Systematic Review and Meta-Analysis. | Exclude - mechanism not intervention |
| 66 | Pourmoradian (267) | 2021 | Television food advertisements and childhood obesity: A systematic review | Exclude - mechanism not intervention |
| 67 | Chemas-Velez (268) | 2020 | Scoping review of studies on food marketing in Latin America: Summary of existing evidence and research gaps | Exclude - mechanism not intervention |
| 68 | Villegas-Navas (269) | 2020 | The Effects of Foods Embedded in Entertainment Media on Children's Food Choices and Food Intake: A Systematic Review and Meta-Analyses. | Exclude - mechanism not intervention |
| 69 | Elliott (270) | 2019 | Measuring the Power of Food Marketing to Children: a Review of Recent Literature. | Exclude - mechanism not intervention |
| 70 | Smith (271) | 2019 | Food marketing influences children's attitudes, preferences and consumption: A systematic critical review | Exclude - mechanism not intervention |
| 71 | Truman (272) | 2019 | Identifying food marketing to teenagers: a scoping review. | Exclude - mechanism not intervention |
| 72 | Folkvord (273) | 2018 | The persuasive effect of advergames promoting unhealthy foods among children: A meta-analysis. | Exclude - mechanism not intervention |
| 73 | Prowse (274) | 2017 | Food marketing to children in Canada: a settings-based scoping review on exposure, power and impact. | Exclude - mechanism not intervention |
| 74 | Norman (275) | 2016 | The Impact of Marketing and Advertising on Food Behaviours: Evaluating the Evidence for a Causal Relationship | Exclude - mechanism not intervention |
| 75 | Kelly (276) | 2015 | A hierarchy of unhealthy food promotion effects: identifying methodological approaches and knowledge gaps. | Exclude - mechanism not intervention |
| 76 | Kraak (277) | 2015 | Influence of food companies' brand mascots and entertainment companies' cartoon media characters on children's diet and health: a systematic review and research needs. | Exclude - mechanism not intervention |
| 77 | Cairns (278) | 2013 | Systematic reviews of the evidence on the nature, extent and effects of food marketing to children. A retrospective summary. | Exclude - mechanism not intervention |
| 78 | Chavez-Ugalde (279) | 2021 | Conceptualizing the commercial determinants of dietary behaviors associated with obesity: A systematic review using principles from critical interpretative synthesis | Exclude - mechanism not intervention |
| 79 | Buchanan (280) | 2018 | The effects of digital marketing of unhealthy commodities on young people: a systematic review. | Exclude - mechanism not intervention |
| 80 | Nguyen (281) | 2019 | A Systematic Review on the Effects of Personalized Price Promotions for Food Products. | Exclude - mechanism not intervention |
| 81 | Castro (282) | 2018 | Customer Purchase Intentions and Choice in Food Retail Environments: A Scoping Review (282) | Exclude - mechanism not intervention |
| 82 | Haby (283) | 2016 | Agriculture, food, and nutrition interventions that facilitate sustainable food production and impact health: an overview of systematic reviews. | Exclude - cites reviews |
| 83 | Hansen (284) | 2022 | Effectiveness of food environment policies in improving population diets: a review of systematic reviews. | Exclude - cites reviews |
| 84 | Hyseni (285) | 2017 | The effects of policy actions to improve population dietary patterns and prevent diet-related non-communicable diseases: scoping review. | Exclude - cites reviews |
| 85 | Kirkpatrick (286) | 2018 | Gaps in the Evidence on Population Interventions to Reduce Consumption of Sugars: A Review of Reviews. | Exclude - cites reviews |
| 86 | Lobstein (287) | 2020 | Costs, equity and acceptability of three policies to prevent obesity: A narrative review to support policy development | Exclude - cites reviews |
| 87 | Roberts (288) | 2019 | Efficacy of population-wide diabetes and obesity prevention programs: An overview of systematic reviews on proximal, intermediate, and distal outcomes and a meta-analysis of impact on BMI. | Exclude - cites reviews |
| 88 | Shemilt (289) | 2013 | Economic instruments for population diet and physical activity behaviour change: a systematic scoping review. | Exclude - cites reviews |
| 89 | Almiron-Roig (290) | 2020 | A review of evidence supporting current strategies, challenges, and opportunities to reduce portion sizes | Exclude - cites reviews |
| 90 | Hyseni (291) | 2017 | Systematic review of dietary trans-fat reduction interventions. | Exclude - cites reviews |
| 91 | Claudy (292) | 2021 | Are Sugar-Sweetened Beverage Taxes Effective? Reviewing the Evidence Through a Marketing Systems Lens | Exclude - cites reviews |
| 92 | Milani (293) | 2019 | An Umbrella Review and Narrative Synthesis of the Effectiveness of Interventions Aimed at Decreasing Food Prices to Increase Food Quality. | Exclude - cites reviews |
| 93 | Niebylski (294) | 2015 | Healthy food subsidies and unhealthy food taxation: A systematic review of the evidence. | Exclude - cites reviews |
| 94 | Granheim (295) | 2022 | Mapping the digital food environment: A systematic scoping review. | Exclude - cites reviews |
| 95 | Wright (296) | 2018 | Interventions to promote healthy eating choices when dining out: A systematic review of reviews. | Exclude - cites reviews |
| 96 | Cairns (297) | 2019 | A critical review of evidence on the sociocultural impacts of food marketing and policy implications | Exclude - cites reviews |

**References**

1. Rodgers RF, Paxton SJ, Wertheim EH. #Take idealized bodies out of the picture: A scoping review of social media content aiming to protect and promote positive body image. Body Image. 2021;38:10-36.

2. Feng C, Wohn DY, editors. Categorizing Online Harassment Interventions. 2020 IEEE International Symposium on Technology and Society (ISTAS); 2020 12-15 Nov. 2020.

3. Bychkov D, Young SD. Facing Up to Nomophobia: A Systematic Review of Mobile Phone Apps that Reduce Smartphone Usage. In: Roy SS, Samui P, Deo R, Ntalampiras S, editors. Big Data in Engineering Applications. Singapore: Springer Singapore; 2018. p. 161-71.

4. Tiidenberg K. Sex, power and platform governance. Porn Studies. 2021;8(4):381-93.

5. Pennycook G, Rand DG. Accuracy prompts are a replicable and generalizable approach for reducing the spread of misinformation. Nat Commun. 2022;13(1):2333.

6. Danthinne ES, Giorgianni FE, Rodgers RF. Labels to prevent the detrimental effects of media on body image: A systematic review and meta-analysis. Int J Eat Disord. 2020;53(5):377-91.

7. Robinson J, Cox G, Bailey E, Hetrick S, Rodrigues M, Fisher S, et al. Social media and suicide prevention: a systematic review. Early Interv Psychiatry. 2016;10(2):103-21.

8. Bodhi R, Singh T, Rahman S. Recent themes in social media research: a systematic review. International Journal of Business Information Systems. 2021;37(3):287-307.

9. Throuvala MA, Griffiths MD, Rennoldson M, Kuss DJ. School-based Prevention for Adolescent Internet Addiction: Prevention is the Key. A Systematic Literature Review. Curr Neuropharmacol. 2019;17(6):507-25.

10. Gaffney H, Farrington DP, Espelage DL, Ttofi MM. Are cyberbullying intervention and prevention programs effective? A systematic and meta-analytical review. Aggression and Violent Behavior. 2019;45:134-53.

11. Lancaster M. A Systematic Research Synthesis on Cyberbullying Interventions in the United States. Cyberpsychol Behav Soc Netw. 2018;21(10):593-602.

12. Ademiluyi A, Li C, Park A. Implications and Preventions of Cyberbullying and Social Exclusion in Social Media: Systematic Review. JMIR Form Res. 2022;6(1):e30286.

13. Bashir Shaikh F, Rehman M, Amin A. Cyberbullying: A Systematic Literature Review to Identify the Factors Impelling University Students Towards Cyberbullying. IEEE Access. 2020;8:148031-51.

14. Al-Sarem M, Boulila W, Al-Harby M, Qadir J, Alsaeedi A. Deep Learning-Based Rumor Detection on Microblogging Platforms: A Systematic Review. IEEE Access. 2019;7:152788-812.

15. Almazyad F, Loiacono ET. Control vs Content: A Systematic Review of the Social Media Research Literature Proceedings of the 53rd Hawaii International Conference on System Sciences; Hawaii University of Hawaiʻi at Mānoa; 2020.

16. Ben Sassi I, Ben Yahia S. Malicious accounts detection from online social networks: a systematic review of literature. International Journal of General Systems. 2021;50(7):741-814.

17. Mitchell D, El-Gayar O. The Effect of Privacy Policies on Information Sharing Behavior on Social Networks: A Systematic Literature Review. Hawaii International Conference on System Sciences 2020; University of Hawaiʻi at Mānoa2020.

18. Picardo J, McKenzie SK, Collings S, Jenkin G. Suicide and self-harm content on Instagram: A systematic scoping review. PLoS One. 2020;15(9):e0238603.

19. Marchant A, Hawton K, Burns L, Stewart A, John A. Impact of Web-Based Sharing and Viewing of Self-Harm-Related Videos and Photographs on Young People: Systematic Review. J Med Internet Res. 2021;23(3):e18048.

20. Al-Samarraie H, Bello K-A, Alzahrani AI, Smith AP, Emele C. Young users' social media addiction: causes, consequences and preventions. Information Technology & People. 2022;35(7):2314-43.

21. Barsaiyan S, Sijoria C. Twitter Blue Tick - A Study of its Impact on Society. Indian Journal of Marketing. 2021.

22. Blaya C. Cyberhate: A review and content analysis of intervention strategies. Aggression and Violent Behavior. 2019;45:163-72.

23. Charles A, Hare-Duke L, Nudds H, Franklin D, Llewellyn-Beardsley J, Rennick-Egglestone S, et al. Typology of content warnings and trigger warnings: Systematic review. PLoS One. 2022;17(5):e0266722.

24. Orben A. Teenagers, screens and social media: a narrative review of reviews and key studies. Soc Psychiatry Psychiatr Epidemiol. 2020;55(4):407-14.

25. Baccini M, Carreras G. Analyzing and comparing the association between control policy measures and alcohol consumption in Europe. Subst Use Misuse. 2014;49(12):1684-91.

26. Galizzi MM. Label, nudge or tax? A review of health policies for risky behaviours. J Public Health Res. 2012;1(1):14-21.

27. Giesbrecht N, Wettlaufer A, Stockwell T, Vallance K, Chow C, April N, et al. Alcohol retail privatisation in Canadian provinces between 2012 and 2017. Is decision making oriented to harm reduction? Drug Alcohol Rev. 2021;40(3):459-67.

28. Watterson JR, Gabbe B, Rosenfeld JV, Ball H, Romero L, Dietze P. Workplace intervention programmes for decreasing alcohol use in military personnel: a systematic review. BMJ Mil Health. 2021;167(3):192-200.

29. Witt K, Chitty KM, Wardhani R, Varnik A, de Leo D, Kolves K. Effect of alcohol interventions on suicidal ideation and behaviour: A systematic review and meta-analysis. Drug Alcohol Depend. 2021;226:108885.

30. Erng MN, Smirnov A, Reid N. Prevention of Alcohol-Exposed Pregnancies and Fetal Alcohol Spectrum Disorder Among Pregnant and Postpartum Women: A Systematic Review. Alcohol Clin Exp Res. 2020;44(12):2431-48.

31. Tremblay M, Baydala L, Khan M, Currie C, Morley K, Burkholder C, et al. Primary Substance Use Prevention Programs for Children and Youth: A Systematic Review. Pediatrics. 2020;146(3).

32. Armstrong-Moore R, Haighton C, Davinson N, Ling J. Interventions to reduce the negative effects of alcohol consumption in older adults: a systematic review. BMC Public Health. 2018;18(1):302.

33. Kelly S, Olanrewaju O, Cowan A, Brayne C, Lafortune L. Interventions to prevent and reduce excessive alcohol consumption in older people: a systematic review and meta-analysis. Age Ageing. 2018;47(2):175-84.

34. Reid N, Scholin L, Erng MN, Montag A, Hanson J, Smith L. Preconception interventions to reduce the risk of alcohol-exposed pregnancies: A systematic review. Alcohol Clin Exp Res. 2021;45(12):2414-29.

35. Montag A, Clapp JD, Calac D, Gorman J, Chambers C. A review of evidence-based approaches for reduction of alcohol consumption in Native women who are pregnant or of reproductive age. Am J Drug Alcohol Abuse. 2012;38(5):436-43.

36. Plotnikoff RC, Costigan SA, Kennedy SG, Robards SL, Germov J, Wild C. Efficacy of interventions targeting alcohol, drug and smoking behaviors in university and college students: A review of randomized controlled trials. J Am Coll Health. 2019;67(1):68-84.

37. Yuvaraj K, Eliyas SK, Gokul S, Manikandanesan S. Effectiveness of Workplace Intervention for Reducing Alcohol Consumption: a Systematic Review and Meta-Analysis. Alcohol Alcohol. 2019;54(3):264-71.

38. Prosser T, Gee KA, Jones F. A meta-analysis of effectiveness of E-interventions to reduce alcohol consumption in college and university students. J Am Coll Health. 2018;66(4):292-301.

39. Riper H, Hoogendoorn A, Cuijpers P, Karyotaki E, Boumparis N, Mira A, et al. Effectiveness and treatment moderators of internet interventions for adult problem drinking: An individual patient data meta-analysis of 19 randomised controlled trials. PLoS Med. 2018;15(12):e1002714.

40. Scott-Sheldon LA, Carey KB, Kaiser TS, Knight JM, Carey MP. Alcohol Interventions for Greek Letter Organizations: A Systematic Review and Meta-Analysis, 1987 to 2014. Health Psychol. 2016.

41. Agabio R, Trincas G, Floris F, Mura G, Sancassiani F, Angermeyer MC. A Systematic Review of School-Based Alcohol and other Drug Prevention Programs. Clin Pract Epidemiol Ment Health. 2015;11(Suppl 1 M6):102-12.

42. Scott-Sheldon LA, Carey KB, Elliott JC, Garey L, Carey MP. Efficacy of alcohol interventions for first-year college students: a meta-analytic review of randomized controlled trials. J Consult Clin Psychol. 2014;82(2):177-88.

43. Strom HK, Adolfsen F, Fossum S, Kaiser S, Martinussen M. Effectiveness of school-based preventive interventions on adolescent alcohol use: a meta-analysis of randomized controlled trials. Subst Abuse Treat Prev Policy. 2014;9:48.

44. Lee NK, Roche AM, Duraisingam V, Fischer J, Cameron J, Pidd K. A Systematic Review of Alcohol Interventions Among Workers in Male-Dominated Industries. Journal of Men's Health. 2014;11(2):53-63.

45. Foxcroft DR, Tsertsvadze A. Universal alcohol misuse prevention programmes for children and adolescents: Cochrane systematic reviews. Perspect Public Health. 2012;132(3):128-34.

46. Champion KE, Newton NC, Teesson M. Prevention of alcohol and other drug use and related harm in the digital age: what does the evidence tell us? Curr Opin Psychiatry. 2016;29(4):242-9.

47. Boumans J, van de Mheen D, Crutzen R, Dupont H, Bovens R, Rozema A. Understanding How and Why Alcohol Interventions Prevent and Reduce Problematic Alcohol Consumption among Older Adults: A Systematic Review. Int J Environ Res Public Health. 2022;19(6).

48. Oh SS, Moon JY, Chon D, Mita C, Lawrence JA, Park EC, et al. Effectiveness of Digital Interventions for Preventing Alcohol Consumption in Pregnancy: Systematic Review and Meta-analysis. J Med Internet Res. 2022;24(4):e35554.

49. Ahankari AS, Wray J, Jomeen J, Hayter M. The effectiveness of combined alcohol and sexual risk taking reduction interventions on the sexual behaviour of teenagers and young adults: a systematic review. Public Health. 2019;173:83-96.

50. McFadyen T, Chai LK, Wyse R, Kingsland M, Yoong SL, Clinton-McHarg T, et al. Strategies to improve the implementation of policies, practices or programmes in sporting organisations targeting poor diet, physical inactivity, obesity, risky alcohol use or tobacco use: a systematic review. BMJ Open. 2018;8(9):e019151.

51. Pena S, Sierralta P, Norambuena P, Leyton F, Pemjean A, Roman F. Alcohol policy in Chile: a systematic review of policy developments and evaluations. Addiction. 2021;116(3):438-56.

52. Moss AC, Albery IP. The Science of Absent Evidence: Is There Such Thing as an Effective Responsible Drinking Message? Alcohol Alcohol. 2018;53(1):26-30.

53. Gray HM, Wiley RC, Williams PM, Shaffer HJ. A Scoping Review of "Responsible Drinking" Interventions. Health Commun. 2021;36(2):236-56.

54. Sherk A, Stockwell T, Chikritzhs T, Andreasson S, Angus C, Gripenberg J, et al. Alcohol Consumption and the Physical Availability of Take-Away Alcohol: Systematic Reviews and Meta-Analyses of the Days and Hours of Sale and Outlet Density. J Stud Alcohol Drugs. 2018;79(1):58-67.

55. Noel JK, Babor TF. Does industry self-regulation protect young people from exposure to alcohol marketing? A review of compliance and complaint studies. Addiction. 2017;112 Suppl 1:51-6.

56. Colbert S, Wilkinson C, Thornton L, Feng X, Richmond R. Online alcohol sales and home delivery: An international policy review and systematic literature review. Health Policy. 2021;125(9):1222-37.

57. Hollands GJ, Carter P, Anwer S, King SE, Jebb SA, Ogilvie D, et al. Altering the availability or proximity of food, alcohol, and tobacco products to change their selection and consumption. Cochrane Database Syst Rev. 2019;9(9):CD012573.

58. Wettlaufer A. Can a Label Help me Drink in Moderation? A Review of the Evidence on Standard Drink Labelling. Subst Use Misuse. 2018;53(4):585-95.

59. Noel JK, Babor TF, Robaina K. Industry self-regulation of alcohol marketing: a systematic review of content and exposure research. Addiction. 2017;112 Suppl 1:28-50.

60. Holmes J, Guo Y, Maheswaran R, Nicholls J, Meier PS, Brennan A. The impact of spatial and temporal availability of alcohol on its consumption and related harms: a critical review in the context of UK licensing policies. Drug Alcohol Rev. 2014;33(5):515-25.

61. Rehm J, Crepault JF, Hasan OSM, Lachenmeier DW, Room R, Sornpaisarn B. Regulatory Policies for Alcohol, other Psychoactive Substances and Addictive Behaviours: The Role of Level of Use and Potency. A Systematic Review. Int J Environ Res Public Health. 2019;16(19).

62. Finan LJ, Lipperman-Kreda S, Grube JW, Balassone A, Kaner E. Alcohol Marketing and Adolescent and Young Adult Alcohol Use Behaviors: A Systematic Review of Cross-Sectional Studies. J Stud Alcohol Drugs Suppl. 2020;Sup 19(Suppl 19):42-56.

63. Curtis BL, Lookatch SJ, Ramo DE, McKay JR, Feinn RS, Kranzler HR. Meta-Analysis of the Association of Alcohol-Related Social Media Use with Alcohol Consumption and Alcohol-Related Problems in Adolescents and Young Adults. Alcohol Clin Exp Res. 2018;42(6):978-86.

64. Brown K. Association Between Alcohol Sports Sponsorship and Consumption: A Systematic Review. Alcohol Alcohol. 2016;51(6):747-55.

65. Stautz K, Brown KG, King SE, Shemilt I, Marteau TM. Immediate effects of alcohol marketing communications and media portrayals on consumption and cognition: a systematic review and meta-analysis of experimental studies. BMC Public Health. 2016;16:465.

66. Nelson JP. Gender differences in alcohol demand: a systematic review of the role of prices and taxes. Health Econ. 2014;23(10):1260-80.

67. Bryden A, Roberts B, McKee M, Petticrew M. A systematic review of the influence on alcohol use of community level availability and marketing of alcohol. Health Place. 2012;18(2):349-57.

68. Noel JK, Sammartino CJ, Rosenthal SR. Exposure to Digital Alcohol Marketing and Alcohol Use: A Systematic Review. J Stud Alcohol Drugs Suppl. 2020;Sup 19(Suppl 19):57-67.

69. Scott S, Muirhead C, Shucksmith J, Tyrrell R, Kaner E. Does Industry-Driven Alcohol Marketing Influence Adolescent Drinking Behaviour? A Systematic Review. Alcohol Alcohol. 2017;52(1):84-94.

70. Khamis AA, Salleh SZ, Ab Karim MS, Mohd Rom NA, Janasekaran S, Idris A, et al. Alcohol Consumption Patterns: A Systematic Review of Demographic and Sociocultural Influencing Factors. Int J Environ Res Public Health. 2022;19(13).

71. Herrera Amul GG. Alcohol Advertising, Promotion, and Sponsorship: A Review of Regulatory Policies in the Association of Southeast Asian Nations. J Stud Alcohol Drugs. 2020;81(6):697-709.

72. Ospina M, Moga C, Dennett L, Harstall C. A Systematic Review of the Effectiveness of Prevention Approaches for Fetal Alcohol Spectrum Disorder. Prevention of Fetal Alcohol Spectrum Disorder FASD. Health Care and Disease Management2011. p. 99-335.

73. Clifford S, Smith JA, Livingston M, Wright CJC, Griffiths KE, Miller PG. A historical overview of legislated alcohol policy in the Northern Territory of Australia: 1979-2021. BMC Public Health. 2021;21(1):1921.

74. Anderson P, Kokole D, Llopis EJ. Production, Consumption, and Potential Public Health Impact of Low- and No-Alcohol Products: Results of a Scoping Review. Nutrients. 2021;13(9).

75. Sharma A, Sinha K, Vandenberg B. Pricing as a means of controlling alcohol consumption. Br Med Bull. 2017;123(1):149-58.

76. Rehm J, Neufeld M, Room R, Sornpaisarn B, Stelemekas M, Swahn MH, et al. The impact of alcohol taxation changes on unrecorded alcohol consumption: A review and recommendations. Int J Drug Policy. 2022;99:103420.

77. Mozaffarian D, Afshin A, Benowitz NL, Bittner V, Daniels SR, Franch HA, et al. Population approaches to improve diet, physical activity, and smoking habits: a scientific statement from the American Heart Association. Circulation. 2012;126(12):1514-63.

78. Napirah MR, Amiruddin R, Palutturi S, Stang, Vidyanto, Nur R, et al. A Model of Cigarette Advertisement Policy in Preventing Children Smoking Habits in Palu City, Indonesia: A Systematic Review. Indian Journal of Forensic Medicine & Toxicology. 2020.

79. Rahman MA, Hann N, Wilson A, Worrall-Carter L. Electronic cigarettes: patterns of use, health effects, use in smoking cessation and regulatory issues. Tob Induc Dis. 2014;12(1):21.

80. Omare MO, Kibet JK, Cherutoi JK, Kengara FO. A review of tobacco abuse and its epidemiological consequences. Z Gesundh Wiss. 2022;30(6):1485-500.

81. Coppo A, Galanti MR, Giordano L, Buscemi D, Bremberg S, Faggiano F. School policies for preventing smoking among young people. Cochrane Database Syst Rev. 2014;2014(10):Cd009990.

82. Brown N, Luckett T, Davidson PM, Di Giacomo M. Interventions to reduce harm from smoking with families in infancy and early childhood: a systematic review. Int J Environ Res Public Health. 2015;12(3):3091-119.

83. O'Connell N, Burke E, Dobbie F, Dougall N, Mockler D, Darker C, et al. The effectiveness of smoking cessation interventions for socio-economically disadvantaged women: a systematic review and meta-analysis. Syst Rev. 2022;11(1):111.

84. Adıbelli N. Effectiveness of Interventions to Reduce Water Pipe Smoking Behaviors among Adolescents: A Systematic Review. International Journal of Academic Medicine and Pharmacy. 2020;Volume: 2 Issue: 3:313-9.

85. Blaga OM, Vasilescu L, Chereches RM. Use and effectiveness of behavioural economics in interventions for lifestyle risk factors of non-communicable diseases: a systematic review with policy implications. Perspect Public Health. 2018;138(2):100-10.

86. Carson KV, Brinn MP, Labiszewski NA, Peters M, Chang AB, Veale A, et al. Interventions for tobacco use prevention in Indigenous youth. Cochrane Database Syst Rev. 2012;2012(8):CD009325.

87. Dale H, Brassington L, King K. The impact of healthy lifestyle interventions on mental health and wellbeing: a systematic review. Mental Health Review Journal. 2014;19(1):1-26.

88. Evans-Polce RJ, Castaldelli-Maia JM, Schomerus G, Evans-Lacko SE. The downside of tobacco control? Smoking and self-stigma: A systematic review. Soc Sci Med. 2015;145:26-34.

89. Huriah T, Lestari V. School-based Smoking Prevention in Adolescents in Developing Countries: A Literature Review. Open Access Macedonian Journal of Medical Sciences. 2020;8:84-9.

90. Kader Z, Roman NV, Crutzen R. Systematic review of interventions aimed at reducing hookah pipe use: Implications for practitioners and clinicians. S Afr Med J. 2019;109(6):392-406.

91. Kohler S, Minkner P. Smoke-free laws and direct democracy initiatives on smoking bans in Germany: a systematic review and quantitative assessment. Int J Environ Res Public Health. 2014;11(1):685-700.

92. Nethan ST, Sinha DN, Chandan K, Mehrotra R. Smokeless tobacco cessation interventions: A systematic review. Indian J Med Res. 2018;148(4):396-410.

93. Nishio A, Saito J, Tomokawa S, Kobayashi J, Makino Y, Akiyama T, et al. Systematic review of school tobacco prevention programs in African countries from 2000 to 2016. PLoS One. 2018;13(2):e0192489.

94. Nwosu C, Angus K, Cheeseman H, Semple S. Reducing Secondhand Smoke Exposure Among Nonsmoking Pregnant Women: A Systematic Review. Nicotine Tob Res. 2020;22(12):2127-33.

95. Parkinson R, Jessiman-Perreault G, Frenette N, Allen Scott LK. Exploring Multilevel Workplace Tobacco Control Interventions: A Scoping Review. Workplace Health Saf. 2022;70(8):368-82.

96. Peer N, Naicker A, Khan M, Kengne AP. A narrative systematic review of tobacco cessation interventions in Sub-Saharan Africa. SAGE Open Med. 2020;8:2050312120936907.

97. Rosen LJ, Myers V, Winickoff JP, Kott J. Effectiveness of Interventions to Reduce Tobacco Smoke Pollution in Homes: A Systematic Review and Meta-Analysis. Int J Environ Res Public Health. 2015;12(12):16043-59.

98. Saroj SK, Bhardwaj T. Non-pharmacological interventions for tobacco cessation: A systematic review of existing practices and their effectiveness. Monaldi Arch Chest Dis. 2022;92(4).

99. Scheffers-van Schayck T, Mujcic A, Otten R, Engels R, Kleinjan M. The Effectiveness of Smoking Cessation Interventions Tailored to Smoking Parents of Children Aged 0-18 Years: A Meta-Analysis. Eur Addict Res. 2021;27(4):278-93.

100. Song R, Park M. Meta-analysis of the effects of smoking prevention programs for young adolescents. Child Health Nurs Res. 2021;27(2):95-110.

101. Soto C, Unger JB, Sussman S, Zeledon I. International Approaches to Tobacco Use Cessation Programming and Prevention Interventions among Indigenous Adolescents and Young Adults. Current Addiction Reports. 2018;5(1):35-41.

102. Stanton A, Grimshaw G. Tobacco cessation interventions for young people. Cochrane Database Syst Rev. 2013(8):CD003289.

103. Suls JM, Luger TM, Curry SJ, Mermelstein RJ, Sporer AK, An LC. Efficacy of smoking-cessation interventions for young adults: a meta-analysis. Am J Prev Med. 2012;42(6):655-62.

104. Taylor GMJ, Dalili MN, Semwal M, Civljak M, Sheikh A, Car J. Internet-based interventions for smoking cessation. Cochrane Database Syst Rev. 2017;9(9):CD007078.

105. Thomas RE, McLellan J, Perera R. School-based programmes for preventing smoking. Cochrane Database Syst Rev. 2013;2013(4):CD001293.

106. Vance L, Glanville B, Ramkumar K, Chambers J, Tzelepis F. The effectiveness of smoking cessation interventions in rural and remote populations: Systematic review and meta-analyses. Int J Drug Policy. 2022;106:103775.

107. Villanti AC, West JC, Klemperer EM, Graham AL, Mays D, Mermelstein RJ, et al. Smoking-Cessation Interventions for U.S. Young Adults: Updated Systematic Review. Am J Prev Med. 2020;59(1):123-36.

108. Washio Y, Cassey H. Systematic Review of Interventions for Racial/Ethnic-Minority Pregnant Smokers. J Smok Cessat. 2016;11(1):12-27.

109. Behbod B, Sharma M, Baxi R, Roseby R, Webster P. Family and carer smoking control programmes for reducing children's exposure to environmental tobacco smoke. Cochrane Database Syst Rev. 2018;1(1):CD001746.

110. Cahill K, Lancaster T. Workplace interventions for smoking cessation. Cochrane Database Syst Rev. 2014(2):CD003440.

111. Stevenson L, Campbell S, Bohanna I, Gould GS, Robertson J, Clough AR. Establishing Smoke-Free Homes in the Indigenous Populations of Australia, New Zealand, Canada and the United States: A Systematic Literature Review. Int J Environ Res Public Health. 2017;14(11).

112. Monson E, Arsenault N. Effects of Enactment of Legislative (Public) Smoking Bans on Voluntary Home Smoking Restrictions: A Review. Nicotine Tob Res. 2017;19(2):141-8.

113. Allen LN, Pullar J, Wickramasinghe KK, Williams J, Roberts N, Mikkelsen B, et al. Evaluation of research on interventions aligned to WHO 'Best Buys' for NCDs in low-income and lower-middle-income countries: a systematic review from 1990 to 2015. BMJ Glob Health. 2018;3(1):e000535.

114. Collins L, Glasser AM, Abudayyeh H, Pearson JL, Villanti AC. E-Cigarette Marketing and Communication: How E-Cigarette Companies Market E-Cigarettes and the Public Engages with E-cigarette Information. Nicotine Tob Res. 2019;21(1):14-24.

115. Drovandi A, Teague PA, Glass B, Malau-Aduli B. A systematic review of smoker and non-smoker perceptions of visually unappealing cigarette sticks. Tob Induc Dis. 2018;16:02.

116. Francis DB, Mason N, Ross JC, Noar SM. Impact of tobacco-pack pictorial warnings on youth and young adults: A systematic review of experimental studies. Tob Induc Dis. 2019;17:41.

117. Gardner K, Kearns R, Woodland L, Silveira M, Hua M, Katz M, et al. A Scoping Review of the Evidence on Health Promotion Interventions for Reducing Waterpipe Smoking: Implications for Practice. Front Public Health. 2018;6:308.

118. Guindon GE, Paraje GR, Chaloupka FJ. The impact of prices and taxes on the use of tobacco products in Latin America and the Caribbean. Am J Public Health. 2015;105(3):e9-19.

119. Hughes N, Arora M, Grills N. Perceptions and impact of plain packaging of tobacco products in low and middle income countries, middle to upper income countries and low-income settings in high-income countries: a systematic review of the literature. BMJ Open. 2016;6(3):e010391.

120. Iqbal S, Barolia R, Petrucka P, Ladak L, Rehmani R, Kabir A. Smoking cessation interventions in South Asian Region: a systematic scoping review. BMC Public Health. 2022;22(1):1096.

121. Janmohamed K, Walter N, Sangngam N, Hampsher S, Nyhan K, De Choudhury M, et al. Interventions to Mitigate Vaping Misinformation: A Meta-Analysis. J Health Commun. 2022;27(2):84-92.

122. Jo CL, Ambs A, Dresler CM, Backinger CL. Child-resistant and tamper-resistant packaging: A systematic review to inform tobacco packaging regulation. Prev Med. 2017;95:89-95.

123. Johnson AC, Mercincavage M, Souprountchouk V, Rogelberg S, Sidhu AK, Delnevo CD, et al. Responses to reduced nicotine cigarette marketing features: a systematic review. Tob Control. 2021.

124. Lee JG, Gregory KR, Baker HM, Ranney LM, Goldstein AO. "May I Buy a Pack of Marlboros, Please?" A Systematic Review of Evidence to Improve the Validity and Impact of Youth Undercover Buy Inspections. PLoS One. 2016;11(4):e0153152.

125. Moodie C, Angus K, Stead M. Consumer Response to Standardized Tobacco Packaging in the United Kingdom: A Synthesis of Evidence from Two Systematic Reviews. Risk Manag Healthc Policy. 2021;14:1465-80.

126. Noar SM, Francis DB, Bridges C, Sontag JM, Brewer NT, Ribisl KM. Effects of Strengthening Cigarette Pack Warnings on Attention and Message Processing: A Systematic Review. Journal Mass Commun Q. 2017;94(2):416-42.

127. Noar SM, Hall MG, Francis DB, Ribisl KM, Pepper JK, Brewer NT. Pictorial cigarette pack warnings: a meta-analysis of experimental studies. Tob Control. 2016;25(3):341-54.

128. Padhiary S, Samal D, Khandayataray P, Murthy MK. A systematic review report on tobacco products and its health issues in India. Rev Environ Health. 2021;36(3):367-89.

129. Pang B, Saleme P, Seydel T, Kim J, Knox K, Rundle-Thiele S. The effectiveness of graphic health warnings on tobacco products: a systematic review on perceived harm and quit intentions. BMC Public Health. 2021;21(1):884.

130. Papanastasiou N, Hill S, Amos A. Evidence From Qualitative Studies of Youth About the Impacts of Tobacco Control Policy on Young People in Europe: A Systematic Review. Nicotine Tob Res. 2019;21(7):863-70.

131. Rando-Matos Y, Pons-Vigues M, Lopez MJ, Cordoba R, Ballve-Moreno JL, Puigdomenech-Puig E, et al. Smokefree legislation effects on respiratory and sensory disorders: A systematic review and meta-analysis. PLoS One. 2017;12(7):e0181035.

132. Stead M, Moodie C, Angus K, Bauld L, McNeill A, Thomas J, et al. Is consumer response to plain/standardised tobacco packaging consistent with framework convention on tobacco control guidelines? A systematic review of quantitative studies. PLoS One. 2013;8(10):e75919.

133. Tee GH, Hairi NN, Nordin F, Choo WY, Chan YY, Kaur G, et al. Systematic review on international practices in controlling waterpipe tobacco smoking. Asian Pac J Cancer Prev. 2015;16(9):3659-65.

134. Munthali GNC, Wu XL, Rizwan M, Daru GR, Shi Y. Assessment of Tobacco Control Policy Instruments, Status and Effectiveness in Africa: A Systematic Literature Review. Risk Manag Healthc Policy. 2021;14:2913-27.

135. Tremblay MC, Pluye P, Gore G, Granikov V, Filion KB, Eisenberg MJ. Regulation profiles of e-cigarettes in the United States: a critical review with qualitative synthesis. BMC Med. 2015;13:130.

136. Vilhelmsson A, Östergren PO. Reducing health inequalities with interventions targeting behavioral factors among individuals with low levels of education - A rapid review. PLoS One. 2018;13(4):e0195774.

137. Sheikh ZD, Branston JR, Gilmore AB. Tobacco industry pricing strategies in response to excise tax policies: a systematic review. Tob Control. 2023;32(2):239-50.

138. Golden SD, Smith MH, Feighery EC, Roeseler A, Rogers T, Ribisl KM. Beyond excise taxes: a systematic review of literature on non-tax policy approaches to raising tobacco product prices. Tob Control. 2016;25(4):377-85.

139. Babaie J, Ahmadi A, Abdollahi G, Doshmangir L. Preventing and controlling water pipe smoking: a systematic review of management interventions. BMC Public Health. 2021;21(1):344.

140. Grilo G, Crespi E, Cohen JE. A scoping review on disparities in exposure to advertising for e-cigarettes and heated tobacco products and implications for advancing a health equity research agenda. Int J Equity Health. 2021;20(1):238.

141. Kyriakos CN, Zatonski MZ, Filippidis FT. Flavour capsule cigarette use and perceptions: a systematic review. Tob Control. 2023;32(e1):e83-e94.

142. Lee JGL, Orlan EN, Sewell KB, Ribisl KM. A new form of nicotine retailers: a systematic review of the sales and marketing practices of vape shops. Tob Control. 2018;27(e1):e70-e5.

143. McCausland K, Maycock B, Leaver T, Jancey J. The Messages Presented in Electronic Cigarette-Related Social Media Promotions and Discussion: Scoping Review. J Med Internet Res. 2019;21(2):e11953.

144. Robertson L, Cameron C, McGee R, Marsh L, Hoek J. Point-of-sale tobacco promotion and youth smoking: a meta-analysis. Tob Control. 2016;25(e2):e83-e9.

145. Lee S, Ling PM, Glantz SA. The vector of the tobacco epidemic: tobacco industry practices in low and middle-income countries. Cancer Causes Control. 2012;23 Suppl 1(0 1):117-29.

146. Amul GGH, Tan GPP, van der Eijk Y. A Systematic Review of Tobacco Industry Tactics in Southeast Asia: Lessons for Other Low- And MiddleIncome Regions. Int J Health Policy Manag. 2021;10(6):324-37.

147. Banks C, Rawaf S, Hassounah S. Factors influencing the tobacco control policy process in Egypt and Iran: a scoping review. Glob Health Res Policy. 2017;2:19.

148. Krishnamoorthy Y, Majella MG, Murali S. Impact of tobacco industry pricing and marketing strategy on brand choice, loyalty and cessation in global south countries: a systematic review. Int J Public Health. 2020;65(7):1057-66.

149. Lee JGL, Kong AY, Sewell KB, Golden SD, Combs TB, Ribisl KM, et al. Associations of tobacco retailer density and proximity with adult tobacco use behaviours and health outcomes: a meta-analysis. Tob Control. 2022;31(e2):e189-e200.

150. Marsh L, Vaneckova P, Robertson L, Johnson TO, Doscher C, Raskind IG, et al. Association between density and proximity of tobacco retail outlets with smoking: A systematic review of youth studies. Health & Place. 2021;67:102275.

151. Nuyts PAW, Davies LEM, Kunst AE, Kuipers MAG. The Association Between Tobacco Outlet Density and Smoking Among Young People: A Systematic Methodological Review. Nicotine Tob Res. 2021;23(2):239-48.

152. Reimold AE, Lee JGL, Ribisl KM. Tobacco company agreements with tobacco retailers for price discounts and prime placement of products and advertising: a scoping review. Tob Control. 2022.

153. Savell E, Gilmore AB, Fooks G. How does the tobacco industry attempt to influence marketing regulations? A systematic review. PLoS One. 2014;9(2):e87389.

154. Smith KE, Savell E, Gilmore AB. What is known about tobacco industry efforts to influence tobacco tax? A systematic review of empirical studies. Tob Control. 2013;22(2):144-53.

155. Travis N, Levy DT, McDaniel PA, Henriksen L. Tobacco retail availability and cigarette and e-cigarette use among youth and adults: a scoping review. Tob Control. 2022;31(e2):e175-e88.

156. Villanti AC, Collins LK, Niaura RS, Gagosian SY, Abrams DB. Menthol cigarettes and the public health standard: a systematic review. BMC Public Health. 2017;17(1):983.

157. Wynne O, Guillaumier A, Twyman L, McCrabb S, Denham AMJ, Paul C, et al. Signs, Fines and Compliance Officers: A Systematic Review of Strategies for Enforcing Smoke-Free Policy. Int J Environ Res Public Health. 2018;15(7).

158. Liber AC, Sanchez-Romero LM, Cadham CJ, Yuan Z, Li Y, Oh H, et al. Tobacco Couponing: A Systematic Review of Exposures and Effects on Tobacco Initiation and Cessation. Nicotine Tob Res. 2022;24(10):1523-33.

159. Napirah MR, Amiruddin R, Palutturi S, Syam A, Mallongi A, Nur R, et al. Implementing a Non-Smoking Regional Policy to Prohibit Childrens’ Smoking Habits In Palu City, Indonesia: A Systematic Review. Malaysian Journal of Medicine and Health Sciences. 2021;17: 43-6.

160. Rosario MH, Johnson DL, Manglallan KS, Phillips KT, Pokhrel P, Okamoto SK. Tobacco use among native Hawaiian and Pacific Islander youth in the U.S. and USAPI: a systematic review of the literature. J Ethn Subst Abuse. 2021:1-16.

161. Xiao D, Bai CX, Chen ZM, Wang C. Implementation of the World Health Organization Framework Convention on Tobacco Control in China: An arduous and long-term task. Cancer. 2015;121 Suppl 17:3061-8.

162. Chamberlain C, Perlen S, Brennan S, Rychetnik L, Thomas D, Maddox R, et al. Evidence for a comprehensive approach to Aboriginal tobacco control to maintain the decline in smoking: an overview of reviews among Indigenous peoples. Syst Rev. 2017;6(1):135.

163. Glasser AM, Roberts ME. Retailer density reduction approaches to tobacco control: A review. Health Place. 2021;67:102342.

164. Haddad L, El-Shahawy O, Ghadban R, Barnett TE, Johnson E. Waterpipe Smoking and Regulation in the United States: A Comprehensive Review of the Literature. Int J Environ Res Public Health. 2015;12(6):6115-35.

165. Hill S, Amos A, Clifford D, Platt S. Impact of tobacco control interventions on socioeconomic inequalities in smoking: review of the evidence. Tob Control. 2014;23(e2):e89-97.

166. Livingstone-Banks J, Lindson N, Hartmann-Boyce J, Aveyard P. Effects of interventions to combat tobacco addiction: Cochrane update of 2019 and 2020 reviews. Addiction. 2022;117(6):1573-88.

167. Mannocci A, Backhaus I, D'Egidio V, Federici A, Villari P, La Torre G. What public health strategies work to reduce the tobacco demand among young people? An umbrella review of systematic reviews and meta-analyses. Health Policy. 2019;123(5):480-91.

168. O'Connell M, Kephart L. Local and State Policy Action Taken in the United States to Address the Emergence of E-Cigarettes and Vaping: A Scoping Review of Literature. Health Promot Pract. 2022;23(1):51-63.

169. Owotomo O. Current Trends and Impact of Smoking Cessation Interventions for Adult Smokers in Low and Middle Income Countries: A Systematic Literature Review. Journal of Smoking Cessation. 2016;11(1):37-49.

170. Palmer AM, Price SN, Foster MG, Sanford BT, Fucito LM, Toll BA. Urgent Need for Novel Investigations of Treatments to Quit E-cigarettes: Findings from a Systematic Review. Cancer Prev Res (Phila). 2022;15(9):569-80.

171. Pratiti R, Mukherjee D. Epidemiology and Adverse Consequences of Hookah/Waterpipe Use: A Systematic Review. Cardiovasc Hematol Agents Med Chem. 2019;17(2):82-93.

172. Puljevic C, Morphett K, Hefler M, Edwards R, Walker N, Thomas DP, et al. Closing the gaps in tobacco endgame evidence: a scoping review. Tob Control. 2022;31(2):365-75.

173. South E, Rodgers M, Wright K, Whitehead M, Sowden A. Reducing lifestyle risk behaviours in disadvantaged groups in high-income countries: A scoping review of systematic reviews. Prev Med. 2022;154:106916.

174. Struik LL, Dow-Fleisner S, Belliveau M, Thompson D, Janke R. Tactics for Drawing Youth to Vaping: Content Analysis of Electronic Cigarette Advertisements. J Med Internet Res. 2020;22(8):e18943.

175. van der Eijk Y, Tan Ping Ping G, Ong SE, Tan Li Xin G, Li D, Zhang D, et al. E-Cigarette Markets and Policy Responses in Southeast Asia: A Scoping Review. Int J Health Policy Manag. 2021;11(9):1616-24.

176. Wolfenden L, Carruthers J, Wyse R, Yoong S. Translation of tobacco control programs in schools: findings from a rapid review of systematic reviews of implementation and dissemination interventions. Health Promot J Austr. 2014;25(2):136-8.

177. Lee JG, Matthews AK, McCullen CA, Melvin CL. Promotion of tobacco use cessation for lesbian, gay, bisexual, and transgender people: a systematic review. Am J Prev Med. 2014;47(6):823-31.

178. Gainsbury SM, Blankers M, Wilkinson C, Schelleman-Offermans K, Cousijn J. Recommendations for international gambling harm-minimisation guidelines: comparison with effective public health policy. J Gambl Stud. 2014;30(4):771-88.

179. Gainsbury SM. Review of self-exclusion from gambling venues as an intervention for problem gambling. J Gambl Stud. 2014;30(2):229-51.

180. Shi J, Colder Carras M, Potenza MN, Turner NE. A Perspective on Age Restrictions and Other Harm Reduction Approaches Targeting Youth Online Gambling, Considering Convergences of Gambling and Videogaming. Front Psychiatry. 2020;11:601712.

181. Yu L, Ma CMS. Youth Gambling in Hong Kong: Prevalence, Psychosocial Correlates, and Prevention. J Adolesc Health. 2019;64(6S):S44-S51.

182. Giménez Lozano JM, Morales Rodríguez FM. Systematic Review: Preventive Intervention to Curb the Youth Online Gambling Problem. Sustainability [Internet]. 2022; 14(11).

183. Hahmann T, Hamilton-Wright S, Ziegler C, Matheson FI. Problem gambling within the context of poverty: a scoping review. International Gambling Studies. 2021;21(2):183-219.

184. Marchica L, Derevensky JL. Examining personalized feedback interventions for gambling disorders: A systematic review. J Behav Addict. 2016;5(1):1-10.

185. Kotter R, Kraplin A, Pittig A, Buhringer G. A Systematic Review of Land-Based Self-Exclusion Programs: Demographics, Gambling Behavior, Gambling Problems, Mental Symptoms, and Mental Health. J Gambl Stud. 2019;35(2):367-94.

186. Matheson FI, Hamilton-Wright S, Kryszajtys DT, Wiese JL, Cadel L, Ziegler C, et al. The use of self-management strategies for problem gambling: a scoping review. BMC Public Health. 2019;19(1):445.

187. Ladouceur R, Blaszczynski A, Lalande DR. Pre-commitment in gambling: a review of the empirical evidence. International Gambling Studies. 2012;12(2):215-30.

188. Paterson M, Whitty M, Boyer C. An overview of digital and online strategies to reduce gambling harm. Health Promot J Austr. 2021;32(2):248-57.

189. Grande-Gosende A, López-Núñez C, García-Fernández G, Derevensky J, Fernández-Hermida JR. Systematic Review of Preventive Programs for Reducing Problem Gambling Behaviors Among Young Adults. J Gambl Stud. 2020;36(1):1-22.

190. Akçayır M, Nicoll F, Baxter DG, Palmer ZS. Whose Responsibility Is It to Prevent or Reduce Gambling Harm? A Mapping Review of Current Empirical Research. International Journal of Mental Health and Addiction. 2022;20(3):1516-36.

191. Raybould JN, Larkin M, Tunney RJ. Is there a health inequality in gambling related harms? A systematic review. BMC Public Health. 2021;21(1):305.

192. Akcayir M, Nicoll F, Baxter DG. Emerging Gambling Problems and Suggested Interventions: A Systematic Review of Empirical Research. J Gambl Stud. 2023;39(2):857-82.

193. Skarupova K, Vlach T, Mravcik V. Early intervention and identification of gambling disorder: a systematic literature review of strategies implemented by gambling operators. Cent Eur J Public Health. 2020;28(1):18-23.

194. Ginley MK, Whelan JP, Pfund RA, Peter SC, Meyers AW. Warning messages for electronic gambling machines: evidence for regulatory policies. Addiction Research & Theory. 2017;25(6):495-504.

195. Ariyabuddhiphongs V. Problem Gambling Prevention: Before, During, and After Measures. International Journal of Mental Health and Addiction. 2013;11(5):568-82.

196. Williams R, West B, Simpson R. Prevention of Problem Gambling: A Comprehensive Review of the Evidence, and Identified Best Practices2012.

197. Chen Y-Y, Chien-Chang Wu K, Yip PSF. Suicide Prevention through Restricting Access to Suicide Means and Hotspots. International Handbook of Suicide Prevention2011. p. 545-60.

198. Panuwet P, Siriwong W, Prapamontol T, Ryan PB, Fiedler N, Robson MG, et al. Agricultural Pesticide Management in Thailand: Situation and Population Health Risk. Environ Sci Policy. 2012;17:72-81.

199. Varghese P, Erickson TB. Pesticide Poisoning Among Children in India: The Need for an Urgent Solution. Glob Pediatr Health. 2022;9:2333794x221086577.

200. Afshari M, Karimi-Shahanjarini A, Khoshravesh S, Besharati F. Effectiveness of interventions to promote pesticide safety and reduce pesticide exposure in agricultural health studies: A systematic review. PLoS One. 2021;16(1):e0245766.

201. Fuhrimann S, Wan C, Blouzard E, Veludo A, Holtman Z, Chetty-Mhlanga S, et al. Pesticide Research on Environmental and Human Exposure and Risks in Sub-Saharan Africa: A Systematic Literature Review. Int J Environ Res Public Health. 2021;19(1).

202. Lopes-Ferreira M, Maleski ALA, Balan-Lima L, Bernardo JTG, Hipolito LM, Seni-Silva AC, et al. Impact of Pesticides on Human Health in the Last Six Years in Brazil. Int J Environ Res Public Health. 2022;19(6).

203. Haby MM, Soares A, Chapman E, Clark R, Korc M, Galvão LA. Interventions that facilitate sustainable development by preventing toxic exposure to chemicals: an overview of systematic reviews. Rev Panam Salud Publica. 2016;39(6):378-86.

204. Bowen DJ, Barrington WE, Beresford SA. Identifying the effects of environmental and policy change interventions on healthy eating. Annu Rev Public Health. 2015;36:289-306.

205. Vandevijvere S, Vanderlee L. Effect of Formulation, Labelling, and Taxation Policies on the Nutritional Quality of the Food Supply. Curr Nutr Rep. 2019;8(3):240-9.

206. Watt TL, Beckert W, Smith RD, Cornelsen L. Reducing consumption of unhealthy foods and beverages through banning price promotions: what is the evidence and will it work? Public Health Nutr. 2020;23(12):2228-33.

207. Ikonen I, Sotgiu F, Aydinli A, Verlegh PWJ. Consumer effects of front-of-package nutrition labeling: an interdisciplinary meta-analysis. Journal of the Academy of Marketing Science. 2020;48(3):360-83.

208. Temple NJ. Front-of-package food labels: A narrative review. Appetite. 2020;144:104485.

209. Nikolaou CK, Hankey CR, Lean ME. Calorie-labelling: does it impact on calorie purchase in catering outlets and the views of young adults? Int J Obes (Lond). 2015;39(3):542-5.

210. Hawley KL, Roberto CA, Bragg MA, Liu PJ, Schwartz MB, Brownell KD. The science on front-of-package food labels. Public Health Nutr. 2013;16(3):430-9.

211. Trude ACB, Lowery CM, Ali SH, Vedovato GM. An equity-oriented systematic review of online grocery shopping among low-income populations: implications for policy and research. Nutr Rev. 2022;80(5):1294-310.

212. Wolfenden L, Nathan NK, Sutherland R, Yoong SL, Hodder RK, Wyse RJ, et al. Strategies for enhancing the implementation of school-based policies or practices targeting risk factors for chronic disease. Cochrane Database Syst Rev. 2017;11(11):CD011677.

213. Balhareth A, Meertens R, Kremers S, Sleddens E. Overweight and obesity among adults in the Gulf States: A systematic literature review of correlates of weight, weight-related behaviours, and interventions. Obes Rev. 2019;20(5):763-93.

214. Grieger JA, Wycherley TP, Johnson BJ, Golley RK. Discrete strategies to reduce intake of discretionary food choices: a scoping review. Int J Behav Nutr Phys Act. 2016;13:57.

215. Robinson E, Thomas J, Aveyard P, Higgs S. What everyone else is eating: a systematic review and meta-analysis of the effect of informational eating norms on eating behavior. J Acad Nutr Diet. 2014;114(3):414-29.

216. Sirasa F, Mitchell LJ, Rigby R, Harris N. Family and community factors shaping the eating behaviour of preschool-aged children in low and middle-income countries: A systematic review of interventions. Prev Med. 2019;129:105827.

217. Wolfenden L, Wyse R, Nichols M, Allender S, Millar L, McElduff P. A systematic review and meta-analysis of whole of community interventions to prevent excessive population weight gain. Prev Med. 2014;62:193-200.

218. Boelsen-Robinson T, Peeters A, Beauchamp A, Chung A, Gearon E, Backholer K. A systematic review of the effectiveness of whole-of-community interventions by socioeconomic position. Obes Rev. 2015;16(9):806-16.

219. Whittemore R, Chao A, Popick R, Grey M. School-based internet obesity prevention programs for adolescents: a systematic literature review. Yale J Biol Med. 2013;86(1):49-62.

220. Saraf DS, Nongkynrih B, Pandav CS, Gupta SK, Shah B, Kapoor SK, et al. A systematic review of school-based interventions to prevent risk factors associated with noncommunicable diseases. Asia Pac J Public Health. 2012;24(5):733-52.

221. Rachmah Q, Martiana T, Mulyono M, Paskarini I, Dwiyanti E, Widajati N, et al. The effectiveness of nutrition and health intervention in workplace setting: a systematic review. J Public Health Res. 2021;11(1).

222. Sawada K, Wada K, Shahrook S, Ota E, Takemi Y, Mori R. Social marketing including financial incentive programs at worksite cafeterias for preventing obesity: a systematic review. Syst Rev. 2019;8(1):66.

223. Tam G, Yeung MPS. A systematic review of the long-term effectiveness of work-based lifestyle interventions to tackle overweight and obesity. Prev Med. 2018;107:54-60.

224. Geaney F, Kelly C, Greiner BA, Harrington JM, Perry IJ, Beirne P. The effectiveness of workplace dietary modification interventions: a systematic review. Prev Med. 2013;57(5):438-47.

225. Aceves-Martins M, Lopez-Cruz L, Garcia-Botello M, Gutierrez-Gomez YY, Moreno-Garcia CF. Interventions to Prevent Obesity in Mexican Children and Adolescents: Systematic Review. Prev Sci. 2022;23(4):563-86.

226. Bagnall AM, Radley D, Jones R, Gately P, Nobles J, Van Dijk M, et al. Whole systems approaches to obesity and other complex public health challenges: a systematic review. BMC Public Health. 2019;19(1):8.

227. Hillier-Brown FC, Bambra CL, Cairns JM, Kasim A, Moore HJ, Summerbell CD. A systematic review of the effectiveness of individual, community and societal-level interventions at reducing socio-economic inequalities in obesity among adults. Int J Obes (Lond). 2014;38(12):1483-90.

228. MacMillan F, George ES, Feng X, Merom D, Bennie A, Cook A, et al. Do Natural Experiments of Changes in Neighborhood Built Environment Impact Physical Activity and Diet? A Systematic Review. Int J Environ Res Public Health. 2018;15(2).

229. Abeykoon AH, Engler-Stringer R, Muhajarine N. Health-related outcomes of new grocery store interventions: a systematic review. Public Health Nutr. 2017;20(12):2236-48.

230. Escaron AL, Meinen AM, Nitzke SA, Martinez-Donate AP. Supermarket and grocery store-based interventions to promote healthful food choices and eating practices: a systematic review. Prev Chronic Dis. 2013;10:E50.

231. Hartmann-Boyce J, Bianchi F, Piernas C, Payne Riches S, Frie K, Nourse R, et al. Grocery store interventions to change food purchasing behaviors: a systematic review of randomized controlled trials. Am J Clin Nutr. 2018;107(6):1004-16.

232. Kraak V, Rincon-Gallardo Patino S, Renukuntla D, Kim E. Progress Evaluation for Transnational Restaurant Chains to Reformulate Products and Standardize Portions to Meet Healthy Dietary Guidelines and Reduce Obesity and Non-Communicable Disease Risks, 2000-2018: A Scoping and Systematic Review to Inform Policy. Int J Environ Res Public Health. 2019;16(15).

233. Colon-Ramos U, Monge-Rojas R, Campos H. Impact of WHO recommendations to eliminate industrial trans-fatty acids from the food supply in Latin America and the Caribbean. Health Policy Plan. 2014;29(5):529-41.

234. Elliott LM, Dalglish SL, Topp SM. Health Taxes on Tobacco, Alcohol, Food and Drinks in Low- and Middle-Income Countries: A Scoping Review of Policy Content, Actors, Process and Context. Int J Health Policy Manag. 2022;11(4):414-28.

235. Eyles H, Ni Mhurchu C, Nghiem N, Blakely T. Food pricing strategies, population diets, and non-communicable disease: a systematic review of simulation studies. PLoS Med. 2012;9(12):e1001353.

236. Kenny TA, Little M, Lemieux T, Griffin PJ, Wesche SD, Ota Y, et al. The Retail Food Sector and Indigenous Peoples in High-Income Countries: A Systematic Scoping Review. Int J Environ Res Public Health. 2020;17(23).

237. Blake MR, Backholer K, Lancsar E, Boelsen-Robinson T, Mah C, Brimblecombe J, et al. Investigating business outcomes of healthy food retail strategies: A systematic scoping review. Obes Rev. 2019;20(10):1384-99.

238. Adam A, Jensen JD. What is the effectiveness of obesity related interventions at retail grocery stores and supermarkets? -a systematic review. BMC Public Health. 2016;16(1):1247.

239. Fuster M, Handley MA, Alam T, Fullington LA, Elbel B, Ray K, et al. Facilitating Healthier Eating at Restaurants: A Multidisciplinary Scoping Review Comparing Strategies, Barriers, Motivators, and Outcomes by Restaurant Type and Initiator. Int J Environ Res Public Health. 2021;18(4).

240. Valdivia Espino JN, Guerrero N, Rhoads N, Simon NJ, Escaron AL, Meinen A, et al. Community-based restaurant interventions to promote healthy eating: a systematic review. Prev Chronic Dis. 2015;12:E78.

241. Thorpe CP, Boelsen-Robinson T, Cameron AJ, Blake MR. Business outcomes of healthy food service initiatives in schools: A systematic review. Obes Rev. 2021;22(8):e13264.

242. Marcano-Olivier MI, Horne PJ, Viktor S, Erjavec M. Using Nudges to Promote Healthy Food Choices in the School Dining Room: A Systematic Review of Previous Investigations. J Sch Health. 2020;90(2):143-57.

243. Gordon K, Dynan L, Siegel R. Healthier Choices in School Cafeterias: A Systematic Review of Cafeteria Interventions. J Pediatr. 2018;203:273-9 e2.

244. Barbour L, Lindberg R, Woods J, Charlton K, Brimblecombe J. Local urban government policies to facilitate healthy and environmentally sustainable diet-related practices: a scoping review. Public Health Nutr. 2022;25(2):471-87.

245. Luongo G, Skinner K, Phillipps B, Yu Z, Martin D, Mah CL. The Retail Food Environment, Store Foods, and Diet and Health among Indigenous Populations: a Scoping Review. Curr Obes Rep. 2020;9(3):288-306.

246. Chung A, Zorbas C, Riesenberg D, Sartori A, Kennington K, Ananthapavan J, et al. Policies to restrict unhealthy food and beverage advertising in outdoor spaces and on publicly owned assets: A scoping review of the literature. Obes Rev. 2022;23(2):e13386.

247. Smithers LG, Lynch JW, Merlin T. Industry self-regulation and TV advertising of foods to Australian children. J Paediatr Child Health. 2014;50(5):386-92.

248. Galbraith-Emami S, Lobstein T. The impact of initiatives to limit the advertising of food and beverage products to children: a systematic review. Obes Rev. 2013;14(12):960-74.

249. Gynell I, Kemps E, Prichard I. The effectiveness of implicit interventions in food menus to promote healthier eating behaviours: A systematic review. Appetite. 2022;173:105997.

250. Benajiba N, Mahrous L, Bernstein J, Aboul-Enein BH. Food Labeling Use by Consumers in Arab countries: A Scoping Review. J Community Health. 2020;45(3):661-74.

251. Feteira-Santos R, Fernandes J, Virgolino A, Alarcao V, Sena C, Vieira CP, et al. Effectiveness of interpretive front-of-pack nutritional labelling schemes on the promotion of healthier food choices: a systematic review. Int J Evid Based Healthc. 2020;18(1):24-37.

252. Rincon-Gallardo PS, Zhou M, Da Silva Gomes F, Lemaire R, Hedrick V, Serrano E, et al. Effects of Menu Labeling Policies on Transnational Restaurant Chains to Promote a Healthy Diet: A Scoping Review to Inform Policy and Research. Nutrients. 2020;12(6).

253. Oostenbach LH, Slits E, Robinson E, Sacks G. Systematic review of the impact of nutrition claims related to fat, sugar and energy content on food choices and energy intake. BMC Public Health. 2019;19(1):1296.

254. Bergallo P, Castagnari V, Fernandez A, Mejia R. Regulatory initiatives to reduce sugar-sweetened beverages (SSBs) in Latin America. PLoS One. 2018;13(10):e0205694.

255. Needham C, Sacks G, Orellana L, Robinson E, Allender S, Strugnell C. A systematic review of the Australian food retail environment: Characteristics, variation by geographic area, socioeconomic position and associations with diet and obesity. Obes Rev. 2020;21(2):e12941.

256. Mackenbach JD, Nelissen KGM, Dijkstra SC, Poelman MP, Daams JG, Leijssen JB, et al. A Systematic Review on Socioeconomic Differences in the Association between the Food Environment and Dietary Behaviors. Nutrients. 2019;11(9).

257. Bennett R, Zorbas C, Huse O, Peeters A, Cameron AJ, Sacks G, et al. Prevalence of healthy and unhealthy food and beverage price promotions and their potential influence on shopper purchasing behaviour: A systematic review of the literature. Obes Rev. 2020;21(1):e12948.

258. Maganja D, Miller M, Trieu K, Scapin T, Cameron A, Wu JHY. Evidence Gaps in Assessments of the Healthiness of Online Supermarkets Highlight the Need for New Monitoring Tools: a Systematic Review. Curr Atheroscler Rep. 2022;24(4):215-33.

259. Xin J, Zhao L, Wu T, Zhang L, Li Y, Xue H, et al. Association between access to convenience stores and childhood obesity: A systematic review. Obes Rev. 2021;22 Suppl 1(Suppl 1):e12908.

260. Stevenson AC, Brazeau AS, Dasgupta K, Ross NA. Neighbourhood retail food outlet access, diet and body mass index in Canada: a systematic review. Health Promot Chronic Dis Prev Can. 2019;39(10):261-80.

261. Turbutt C, Richardson J, Pettinger C. The impact of hot food takeaways near schools in the UK on childhood obesity: a systematic review of the evidence. J Public Health (Oxf). 2019;41(2):231-9.

262. Cobb LK, Appel LJ, Franco M, Jones-Smith JC, Nur A, Anderson CA. The relationship of the local food environment with obesity: A systematic review of methods, study quality, and results. Obesity (Silver Spring). 2015;23(7):1331-44.

263. Williams J, Scarborough P, Matthews A, Cowburn G, Foster C, Roberts N, et al. A systematic review of the influence of the retail food environment around schools on obesity-related outcomes. Obes Rev. 2014;15(5):359-74.

264. Mc Carthy CM, de Vries R, Mackenbach JD. The influence of unhealthy food and beverage marketing through social media and advergaming on diet-related outcomes in children-A systematic review. Obes Rev. 2022;23(6):e13441.

265. Packer J, Russell SJ, McLaren K, Siovolgyi G, Stansfield C, Viner RM, et al. The impact on dietary outcomes of licensed and brand equity characters in marketing unhealthy foods to children: A systematic review and meta-analysis. Obes Rev. 2022;23(7):e13443.

266. Packer J, Russell SJ, Siovolgyi G, McLaren K, Stansfield C, Viner RM, et al. The Impact on Dietary Outcomes of Celebrities and Influencers in Marketing Unhealthy Foods to Children: A Systematic Review and Meta-Analysis. Nutrients. 2022;14(3).

267. Pourmoradian S, Ostadrahimi A, Bonab AM, Roudsari AH, Jabbari M, Irandoost P. Television food advertisements and childhood obesity: A systematic review. Int J Vitam Nutr Res. 2021;91(1-2):3-9.

268. Chemas-Velez MM, Gomez LF, Velasquez A, Mora-Plazas M, Parra DC. Scoping review of studies on food marketing in Latin America: Summary of existing evidence and research gaps. Rev Saude Publica. 2020;53:107.

269. Villegas-Navas V, Montero-Simo MJ, Araque-Padilla RA. The Effects of Foods Embedded in Entertainment Media on Children's Food Choices and Food Intake: A Systematic Review and Meta-Analyses. Nutrients. 2020;12(4).

270. Elliott C, Truman E. Measuring the Power of Food Marketing to Children: a Review of Recent Literature. Curr Nutr Rep. 2019;8(4):323-32.

271. Smith R, Kelly B, Yeatman H, Boyland E. Food Marketing Influences Children's Attitudes, Preferences and Consumption: A Systematic Critical Review. Nutrients. 2019;11(4).

272. Truman E, Elliott C. Identifying food marketing to teenagers: a scoping review. Int J Behav Nutr Phys Act. 2019;16(1):67.

273. Folkvord F, van 't Riet J. The persuasive effect of advergames promoting unhealthy foods among children: A meta-analysis. Appetite. 2018;129:245-51.

274. Prowse R. Food marketing to children in Canada: a settings-based scoping review on exposure, power and impact. Health Promot Chronic Dis Prev Can. 2017;37(9):274-92.

275. Norman J, Kelly B, Boyland E, McMahon A-T. The Impact of Marketing and Advertising on Food Behaviours: Evaluating the Evidence for a Causal Relationship. Current Nutrition Reports. 2016;5(3):139-49.

276. Kelly B, King ML, Chapman Mnd K, Boyland E, Bauman AE, Baur LA. A hierarchy of unhealthy food promotion effects: identifying methodological approaches and knowledge gaps. Am J Public Health. 2015;105(4):e86-95.

277. Kraak VI, Story M. Influence of food companies' brand mascots and entertainment companies' cartoon media characters on children's diet and health: a systematic review and research needs. Obes Rev. 2015;16(2):107-26.

278. Cairns G, Angus K, Hastings G, Caraher M. Systematic reviews of the evidence on the nature, extent and effects of food marketing to children. A retrospective summary. Appetite. 2013;62:209-15.

279. Chavez-Ugalde Y, Jago R, Toumpakari Z, Egan M, Cummins S, White M, et al. Conceptualizing the commercial determinants of dietary behaviors associated with obesity: A systematic review using principles from critical interpretative synthesis. Obes Sci Pract. 2021;7(4):473-86.

280. Buchanan L, Kelly B, Yeatman H, Kariippanon K. The Effects of Digital Marketing of Unhealthy Commodities on Young People: A Systematic Review. Nutrients. 2018;10(2).

281. Nguyen MTT, Emberger-Klein A, Menrad K. A Systematic Review on the Effects of Personalized Price Promotions for Food Products. Journal of Food Products Marketing. 2019;25(3):257-75.

282. Castro IA, Majmundar A, Williams CB, Baquero B. Customer Purchase Intentions and Choice in Food Retail Environments: A Scoping Review. Int J Environ Res Public Health. 2018;15(11).

283. Haby MM, Chapman E, Clark R, Galvão LA. Agriculture, food, and nutrition interventions that facilitate sustainable food production and impact health: an overview of systematic reviews. Rev Panam Salud Publica. 2016;40(1):48-56.

284. Hansen KL, Golubovic S, Eriksen CU, Jorgensen T, Toft U. Effectiveness of food environment policies in improving population diets: a review of systematic reviews. Eur J Clin Nutr. 2022;76(5):637-46.

285. Hyseni L, Atkinson M, Bromley H, Orton L, Lloyd-Williams F, McGill R, et al. The effects of policy actions to improve population dietary patterns and prevent diet-related non-communicable diseases: scoping review. Eur J Clin Nutr. 2017;71(6):694-711.

286. Kirkpatrick SI, Raffoul A, Maynard M, Lee KM, Stapleton J. Gaps in the Evidence on Population Interventions to Reduce Consumption of Sugars: A Review of Reviews. Nutrients. 2018;10(8).

287. Lobstein T, Neveux M, Landon J. Costs, equity and acceptability of three policies to prevent obesity: A narrative review to support policy development. Obes Sci Pract. 2020;6(5):562-83.

288. Roberts S, Pilard L, Chen J, Hirst J, Rutter H, Greenhalgh T. Efficacy of population-wide diabetes and obesity prevention programs: An overview of systematic reviews on proximal, intermediate, and distal outcomes and a meta-analysis of impact on BMI. Obes Rev. 2019;20(7):947-63.

289. Shemilt I, Hollands GJ, Marteau TM, Nakamura R, Jebb SA, Kelly MP, et al. Economic instruments for population diet and physical activity behaviour change: a systematic scoping review. PLoS One. 2013;8(9):e75070.

290. Almiron-Roig E, Forde CG, Hollands GJ, Vargas MA, Brunstrom JM. A review of evidence supporting current strategies, challenges, and opportunities to reduce portion sizes. Nutr Rev. 2020;78(2):91-114.

291. Hyseni L, Bromley H, Kypridemos C, O'Flaherty M, Lloyd-Williams F, Guzman-Castillo M, et al. Systematic review of dietary trans-fat reduction interventions. Bull World Health Organ. 2017;95(12):821-30G.

292. Claudy M, Doyle G, Marriott L, Campbell N, O’Malley G. Are Sugar-Sweetened Beverage Taxes Effective? Reviewing the Evidence Through a Marketing Systems Lens. Journal of Public Policy & Marketing. 2020;40(3):403-18.

293. Milani C, Lorini C, Baldasseroni A, Dellisanti C, Bonaccorsi G. An Umbrella Review and Narrative Synthesis of the Effectiveness of Interventions Aimed at Decreasing Food Prices to Increase Food Quality. Int J Environ Res Public Health. 2019;16(13).

294. Niebylski ML, Redburn KA, Duhaney T, Campbell NR. Healthy food subsidies and unhealthy food taxation: A systematic review of the evidence. Nutrition. 2015;31(6):787-95.

295. Granheim SI, Lovhaug AL, Terragni L, Torheim LE, Thurston M. Mapping the digital food environment: A systematic scoping review. Obes Rev. 2022;23(1):e13356.

296. Wright B, Bragge P. Interventions to promote healthy eating choices when dining out: A systematic review of reviews. Br J Health Psychol. 2018;23(2):278-95.

297. Cairns G. A critical review of evidence on the sociocultural impacts of food marketing and policy implications. Appetite. 2019;136:193-207.
