# Supplementary material for: Addressing the commercial determinants of mental health: an umbrella review of population-level interventions
Source: Health Promot Int. 2024 Nov 21;39(6):daae147. doi: 10.1093/heapro/daae147 (PMC11579613; doi:10.1093/heapro/daae147)
Supplement: daae147_suppl_Supplementary_Files_5 [file daae147_suppl_supplementary_files_5.docx]

**Supplementary file 5: AMSTAR ratings for reviews with mental health outcomes**

**Item 2: Did the report of the review contain an explicit statement that the review methods were established prior to the conduct of the review and did the report justify any significant deviations from the protocol?**

| **Author** | **Year** | Written protocol | with review question(s) | a search strategy | inclusion/ exclusion criteria | a risk of bias assessment | protocol is registered | meta-analysis/ synthesis plan, if appropriate | plan for investigating causes of heterogeneity | protocol deviations justified | **Item 2 rating** | **Comments** |
| --- | --- | --- | --- | --- | --- | --- | --- | --- | --- | --- | --- | --- |
| Roodbeen | 2021 | No | no | no | no | no | No | Yes | Yes | No deviations discussed | No | No mention of protocol (nor a prisma checklist which describes the presence of protocol) |
| Kolves | 2020 | Yes | Cannot access | Cannot access | Cannot access | Cannot access | No | Yes | Yes | No deviations discussed | No | Searches were conducted "according to the protocol" |
| Nepal | 2020 | Yes | Yes | Yes | Yes | Yes | Yes | Yes | No | No deviations discussed | Partial yes | pre-registered the review: CRD42015027584 |
| Muhunthan | 2017 | No | no | no | no | no | No | Yes | Yes | No deviations discussed | No | No mention of protocol (nor a prisma checklist which describes the presence of protocol) |
| Nelson | 2016 | No | no | no | no | no | No | Yes | Yes | No deviations discussed | No | No mention of protocol (nor a prisma checklist which describes the presence of protocol) |
| Hahn | 2012 | No | no | no | no | no | No | Yes | Yes | No deviations discussed | No | No mention of protocol (nor a prisma checklist which describes the presence of protocol) |
| Reifels | 2019 | No | no | no | no | no | No | Yes | Yes | No deviations discussed | No | No mention of protocol |
| Gunnell | 2017 | Yes | Yes | Yes | Yes | Yes | Yes | Yes | Yes | No deviations discussed | Yes | Registered with prospero: CRD42017053329 |

**Item 4. Did the review authors use a comprehensive literature search strategy?**

| **Author** | **Year** | Searched at least 2 relevant databases | provided key word and/or search strategy | Justified publication restrictions (e.g. language) | searched the ref lists / bibliographies of included studies | Searched trial/study registries | included/ consulted content experts in the field | where relevant, searched for grey literature | conducted search within 24 months of completion of the review | **Item 4 rating** | **Comments** |
| --- | --- | --- | --- | --- | --- | --- | --- | --- | --- | --- | --- |
| Roodbeen | 2021 | Yes | Yes | No - English/Dutch limits not justified | Yes | Not applicable | Yes | Yes | Yes | No | Web of Science, Sociological abstracts, PubMed, PsycINFO and Embase. Full details in supp file; hand-searching the references of included studies, No timespan was selected for the search. |
| Kolves | 2020 | Yes | Yes | No - English limits not justified | No | Yes | Yes - authors | No | Yes | No | Cochrane CENTRAL, Cochrane DARE, EMBASE, Medline, ProQuest, PsycINFO, PubMed, SCOPUS, and Web of Science electronic databases for English-language papers without date restriction until 19 March 2019 |
| Nepal | 2020 | Yes | Yes | No limits applied | Yes | Not applicable | Yes | No | Yes | Partial yes | Included studies up to dec 2018 (published jan 2020); CINAHL, Embase, Google Scholar, Medline, Medline In-Process, ProQuest, PsycINFO, Scopus; no language limits |
| Muhunthan | 2017 | Yes | Yes, online suppement | No limits applied | Yes | No | No | No | Yes | Partial yes | Searches Dec 2015 (Published jan 17) MEDLINE, EMBASE, CINAHL, PsycINFO from inception; Studies were not excluded on the basis of language; Reference lists of included papers reviewed; |
| Nelson | 2016 | Yes | Yes | No - English limits not justified | No | Not applicable | Yes - author | No | Y3s | No | PubMed and IARD Research Database, studies published during 2003–2015; IARD database dates back to 2003; |
| Hahn | 2012 | Yes | No - link provided doesn't work | No - English limits not justified | Yes | Not applicable | Yes | Yes | Yes | No | to December 2010. published april 2012; Econlit, PsycINFO, Sociology Abstracts, MEDLINE, Embase, and EtOH, ref list searching and experts |
| Reifels | 2019 | Yes | Yes | No limits applied | No | No | Yes - Authors | No | Yes | No | Embase, Scopus, PsycINFO, Cochrane Library, CINAHL, and PubMed; We did not place any language restrictions or time limitations. Seach = July 4, 2017, publication Epub 2018 Aug; |
| Gunnell | 2017 | Yes | Yes | No - English only not justified | Yes | No | Yes - Authors | No | Yes | No | Citation searches of key publications using Google Scholar to identify additional articles. |

**Item 7. Did the review authors provide a list of excluded studies and justify the exclusions?**

| **Author** | **Year** | provided a list of all potentially relevant studies that were read in full-text form but excluded from the review | Justified the exclusion from the review of each potentially relevant study | **Item 7 rating** | **Comments** |
| --- | --- | --- | --- | --- | --- |
| Roodbeen | 2021 | No | No | No |  |
| Kolves | 2020 | No | No | No |  |
| Nepal | 2020 | No | No | No |  |
| Muhunthan | 2017 | No | No | No |  |
| Nelson | 2016 | No | No | No |  |
| Hahn | 2012 | No | No | No |  |
| Reifels | 2019 | No | No | No |  |
| Gunnell | 2017 | No | No | No |  |

**Item 9. Did the review authors use a satisfactory technique for assessing the risk of bias (RoB) in individual studies that were included in the review? (NRSI = non-randomized studies of interventions)**

| **Author** | **Year** | Unconcealed allocation | lack of blinding of patients and assessors when assessing outcomes | allocation sequence that was not truly random, | Allocation of the reported result from among multiple measurements or analyses of a specified outcome | Confounding | selection bias | methods used to ascertain exposures and outcomes, | Selection of the reported result from among multiple measurements or analyses of a specified outcome | **Item 9 rating** | **Comments** |
| --- | --- | --- | --- | --- | --- | --- | --- | --- | --- | --- | --- |
| Roodbeen | 2021 | No | No | No | No | No | No | No | No | No | No Risk of Bias assessment undertaken |
| Kolves | 2020 |  |  |  |  | Yes | Yes | Yes | Yes | Yes | Risk Of Bias In Non-randomized Studies of Interventions—of Exposures (ROBINS-E) |
| Nepal | 2020 |  |  |  |  | Yes (3 items) | Yes: | Not relevant | Not relevant? | Yes | We relied on EPOC Guidelines (The Cochrane Collaboration, 2017), the only bias assessment protocol with criteria for ITS studies: "blinding of assessors, selective outcome reporting were not relevant" considered seasonality; inclusion criteria = whole population of the study area |
| Muhunthan | 2017 | No | No | No | No | No | No | No | No | No | No Risk of Bias assessment undertaken |
| Nelson | 2016 |  |  |  |  | No | No | No | No | No | No Risk of Bias assessment undertaken |
| Hahn | 2012 |  |  |  |  | Yes | Yes | Yes | Yes | Yes | Studies with greatest design suitability were those in which data on exposed and comparison populations were collected prospectively. On the basis of the number of threats to validity—such as poor measurement of exposure or outcome, lack of control of potential confounders, or high attrition—studies were characterized as having good (at most one threat to validity), fair (two to four threats), or limited (fıve or more threats) quality of execution. |
| Reifels | 2019 | No | No | No | No | No | No | No | No | No | No Risk of Bias assessment undertaken |
| Gunnell | 2017 |  |  |  |  | Yes | Yes | Yes | Yes | Yes | We used a modified version of the risk of bias criteria for interrupted time series studies suggested by the Cochrane Effective Practice and Organisation of Care. (Table 1) |

**Item 11. If meta-analysis was performed did the review authors use appropriate methods for statistical combination of results?**

| **Author** | **Year** | The authors justified combining the data in a meta-analysis | They used an appropriate weighted technique to combine study results and adjusted for heterogeneity if present | investigated the causes of any heterogeneity | The authors justified combining the data in a meta-analysis | they used an appropriate weighted technique to combine study results, adjusting for heterogeneity if present | they statistically combined effect estimates from NRSI that were adjusted for confounding | They reported separate summary estimates for RCTs and NRSI separately when both were included in the review | **Item 11 rating** | **Comments** |
| --- | --- | --- | --- | --- | --- | --- | --- | --- | --- | --- |
| Roodbeen | 2021 | Not applicable | Not applicable | Not applicable | Not applicable | Not applicable | Not applicable | Not applicable | Not applicable |  |
| Kolves | 2020 | Not applicable | Not applicable | Not applicable | Not applicable | Not applicable | Not applicable | Not applicable | Not applicable |  |
| Nepal | 2020 | Not applicable | Not applicable | Not applicable | Not applicable | Not applicable | Not applicable | Not applicable | Not applicable |  |
| Muhunthan | 2017 | Not applicable | Not applicable | Not applicable | Not applicable | Not applicable | Not applicable | Not applicable | Not applicable |  |
| Nelson | 2016 | Not applicable | Not applicable | Not applicable | Not applicable | Not applicable | Not applicable | Not applicable | Not applicable |  |
| Hahn | 2012 | Not applicable | Not applicable | Not applicable | Not applicable | Not applicable | Not applicable | Not applicable | Not applicable |  |
| Reifels | 2019 | Not applicable | Not applicable | Not applicable | Not applicable | Not applicable | Not applicable | Not applicable | Not applicable |  |
| Gunnell | 2017 | Not applicable | Not applicable | Not applicable | Not applicable | Not applicable | Not applicable | Not applicable | Not applicable |  |

**Item 13. Did the review authors account for Risk of Bias in individual studies when interpreting/ discussing the results of the review?**

| **Author** | **Year** | included only low risk of bias RCTs | Or RCTs with moderate or high RoB, or NRSI were included the review provided a discussion of the likely impact of RoB on the results | **Item 13 rating** | **Comments** |
| --- | --- | --- | --- | --- | --- |
| Roodbeen | 2021 | No | No | No | Adopted a realist approach. |
| Kolves | 2020 | No | Yes | Yes | Discusses RoB (section 3.5) but doesn't link to results…in abstract states " The majority of studies were rated as unclear risk of bias for a number of domains due to a lack of clear reporting" Briefly mentioned in discussion the limitations of ecological study designs |
| Nepal | 2020 | No | Yes | Yes | Table 4, P.15 - describes some ROB findings and possible impact on findings by study. Some mention in discussion, but doesn't link to confidence in findings. Not mentioned in abstract |
| Muhunthan | 2017 | No | No | No | "Interventions were classified as effective if the controls were associated with improvements within the same population or in comparison with other populations, except where authors deemed the results inconclusive due to methodological or reporting biases" but the latter not reported. No mention in discussion/abstract |
| Nelson | 2016 | No | No | No | Discusses limitations of natural experiments, "A review of natural experiments indicates the confidence placed on this measure is too high" but not by individual study. No mention of bias |
| Hahn | 2012 | No | Yes | Yes | Studies with good or fair quality of execution and any level of design suitability (greatest, moderate, or least) qualifıed for the body of evidence; "there is strong evidence". All studies were of fair quality. No discussion of quality/bias in discussion or abstract. |
| Reifels | 2019 | No | Yes | Yes |  |
| Gunnell | 2017 | No | Yes | Yes | At top of discussion, identifies and discusses the 3 reviews with low ROB |

**Item 15. If they performed quantitative synthesis did the review authors carry out an adequate investigation of publication bias (small study bias) and discuss its likely impact on the results of the review?**

| **Author** | **Year** | Performed graphical or statistical tests for publication bias and discussed the likelihood and magnitude of impact of publication bias | **Item 15 rating** | **Comments** |
| --- | --- | --- | --- | --- |
| Roodbeen | 2021 | Not applicable | Not applicable | Not applicable |
| Kolves | 2020 | Not applicable | Not applicable | Not applicable |
| Nepal | 2020 | Not applicable | Not applicable | Not applicable |
| Muhunthan | 2017 | Not applicable | Not applicable | Not applicable |
| Nelson | 2016 | Not applicable | Not applicable | Not applicable |
| Hahn | 2012 | Not applicable | Not applicable | Not applicable |
| Reifels | 2019 | Not applicable | Not applicable | Not applicable |
| Gunnell | 2017 | Not applicable | Not applicable | Not applicable |
