# Supplementary material for: Addressing the commercial determinants of mental health: an umbrella review of population-level interventions
Source: Health Promot Int. 2024 Nov 21;39(6):daae147. doi: 10.1093/heapro/daae147 (PMC11579613; doi:10.1093/heapro/daae147)
Supplement: daae147_suppl_Supplementary_Files_6 [file daae147_suppl_supplementary_files_6.docx]

**Supplementary file six: Included reviews with surrogate outcomes**

**Alcohol**

| **Number** | **Lead Author, Year** | **Intervention** | **Outcome** | **Industry involvement assessment** | |
| --- | --- | --- | --- | --- | --- |
|  |  |  |  | **Rating** | **Rationale** |
| 1 | Carter,  2018 (1) | Bar playlists that don't reference alcohol in their lyrics | Alcohol sales | Apparently independent |  |
| 2 | Gmel,  2016 (2) | Higher licence fees; compliance officers | Assaultive violence | Apparently independent |  |
| 3 | Hassan,  2018 (3) | Alcohol warning labels | Consumption | Apparently independent |  |
| 4 | Kingsland, 2016 (4) | Liquor licence adherence; signage; alcohol service hours: staff training; change in range of products served | Consumption | Apparently independent |  |
| 5 | Kokole,  2021 (5) | New or enhanced alcohol health warning labels | Consumption | Un-declared researcher links with industry found | From another 2021 publication: “Peter Anderson has received funds within the last 5 years from the AB InBev Foundation” |
| 6 | Li,  2015 (6) | Taxation | Consumption | Apparently independent |  |
| 7 | Nelson,  2015 (7) | Taxation, price increases | Binge drinking | Declared study funding from industry sources | Research leading to this paper was supported in part by the International Alliance for Responsible Drinking |
| 8 | Nelson,  2017 (8) | Excise, taxes to increase prices, age restrictions, retail limits, advertising bans | Assaults, other crime, drink driving | Declared study funding from industry sources | Research leading to this paper was supported in part by the International Alliance for Responsible Drinking |
| 9 | Nepal,  2018 (9) | Lockouts: prohibit patrons from entering licensed premises after a designated time | Assault, unintentional injury, drink-driving | Apparently independent |  |
| 10 | Robinson, 2021 (10) | Energy labelling | Consumption | Declared researcher links with industry | Previous researcher funding from American Beverage Association |
| 11 | Sanchez-Ramirez, 2018 (11) | Regulating alcohol trading hours and days | Hospitalisation, assault, violence | Apparently independent |  |
| 12 | Scholes-Balog, 2012 (12) | Alcohol warning labels | Consumption | Apparently independent |  |
| 13 | Siegfried, 2014 (13) | Advertising bans | Consumption | Apparently independent |  |
| 14 | Sileo,  2020 (14) | Staff training | Patron blood alcohol levels | Apparently independent |  |
| 15 | Stockings, 2018 (15) | Responsible service training, Enforcing Underage Drinking Laws | Consumption, drink driving, assaults | Apparently independent |  |
| 16 | Vashishtha, 2020 (16) | Alcopop tax; alcohol advertising regulations and codes of practice; restricting sales to minors; increasing age limits; criminalising secondary supply; increasing purchase price. | Consumption | Apparently independent |  |
| 17 | Wilkinson, 2016 (17) | Reducing the hours during which on premise alcohol outlets can sell alcohol | Violence | Apparently independent |  |
| 18 | Wilson,  2014 (18) | Pricing and taxation studies; hours of sale, alcohol outlet density | Intimate partner violence | Apparently independent |  |

**Tobacco**

| **Number** | **Lead Author, Year** | **Intervention** | **Outcome** | **Industry involvement assessment** | |
| --- | --- | --- | --- | --- | --- |
|  |  |  |  | **Rating** | **Rationale** |

| 1 | Bafunno,  2019 (19) | Tobacco prices, public smoking bans | Consumption | Apparently independent |  |
| --- | --- | --- | --- | --- | --- |
| 2 | Bafunno,  2020 (20) | Tobacco excise taxes and prices; smoke free indoor environments; ban in public places | Consumption | Apparently independent |  |
| 3 | Bardus,  2020 (21) | Smoke-free or tobacco-free university policies | Consumption | Apparently independent |  |
| 4 | Bennet,  2017 (22) | Smoke-free or tobacco-free campus policies, partial smoking restriction | Smoking behaviour | Apparently independent |  |
| 5 | Berman,  2019 (23) | Reducing nicotine levels to minimally or non-addictive levels. | Consumption | Apparently independent |  |
| 6 | Bird,  2020 (24) | Legislative bans; Home smoking ban, work place smoking ban | Smoking rates | Apparently independent |  |
| 7 | Brown,  2014 (25) | Smoke free places; price/tax; advertising controls; warning labels | Socioeconomic inequalities in adult smoking | Apparently independent |  |
| 8 | Brown,  2014 (26) | Smoke free places; price/tax; advertising controls; access controls; warning labels | Socioeconomic inequalities in youth smoking | Apparently independent |  |
| 9 | Cadham,  2020 (27) | Menthol cigarette ban | Consumption | Apparently independent |  |
| 10 | Drovandi, 2019 (28) | Graphic health warnings and/or plain-packaged cigarettes | Smoking behaviour | Apparently independent |  |
| 11 | Forberger, 2022 (29) | Taxation; product bans e.g. flavours; sales/advertisement bans near educational institutions; health warning labels; advertising, promotion &sponsorship controls; limits to sales to minors; packaging & labelling; enforcement & penalties; excise taxes | Smoking behaviour | Apparently independent |  |
| 12 | Frazer,  2016 (30) | Legislative smoking bans | Morbidity, mortality, consumption | Apparently independent |  |
| 13 | Frazer, 2016 (31) | Institutional smoking bans (hospitals, prisons, universities) | Second hand smoke exposure, consumption, | Apparently independent |  |
| 14 | Galanti,  2014 (32) | School policies including bans | Smoking prevalence | Apparently independent |  |
| 15 | Garritsen, 2022 (33) | Smoke-free legislation in any hospitality venue | Consumption | Apparently independent |  |
| 16 | Holland,  2015 (34) | Longer versus shorter cigarettes | Consumption | Apparently independent |  |
| 17 | Jawad,  2016 (35) | Public indoor smoking ban | Demand, cessation | Apparently independent |  |
| 18 | Jetly,  2022 (36) | Plain cigarette packaging | Smoking behaviour | Apparently independent |  |
| 19 | Kienhuis, 2020 (37) | Health warning labels; health warnings at the point-of consumption (eg menus in waterpipe cafes) | Attractiveness, addictiveness,  toxicity | Apparently independent |  |
| 20 | McKay,  2015 (38) | Tax; prohibition of smoking on clinical campuses; health warnings on cigarette and smokeless tobacco packaging | Tobacco use, sales, second hand smoke exposure | Apparently independent |  |
| 21 | McNeill,  2017 (39) | Standardised tobacco packaging | Tobacco use | Apparently independent |  |
| 22 | Minichiello,  2016 (40) | Smoking bans; taxes; Monitoring of compliance with legislation on tobacco sale; sales restrictions | Initiation; consumption; quit rates. | Apparently independent |  |
| 23 | Nanninga, 2019 (41) | Public smoking bans | Second hand smoke exposure | Apparently independent |  |
| 24 | Nazar,  2021 (42) | Tobacco price and taxation | Consumption, affordability | Apparently independent |  |
| 25 | Noar,  2016 (43) | Strengthening cigarette pack warnings (e.g. text to pictorial warnings) | Smoking behaviour | Apparently independent |  |
| 26 | Nuyts,  2018 (44) | Ban on sales to minors | Smoking behaviour | Apparently independent |  |
| 27 | Rado,  2021 (45) | Smoke-free policies in outdoor areas and private places (e.g. cars and schools) | Children's tobacco smoke exposure | Apparently independent |  |
| 28 | Rashiden, 2020 (46) | Smoke-free workplaces | Second hand smoke exposure | Apparently independent |  |
| 29 | Robertson, 2015 (47) | Point of sale promotion restrictions | Smoking behaviour | Apparently independent |  |
| 30 | Rogers,  2022 (48) | Laws restricting the sale of flavoured and menthol tobacco products | Consumption | Apparently independent |  |
| 31 | Schreuders, 2017 (49) | School tobacco policies such as bans | Adolescent smoking behaviour | Apparently independent |  |
| 32 | Shemilt,  2017 (50) | National bans on the use of descriptors such as ‘Light’ and ‘Mild’ on packs | Consumption | Apparently independent |  |
| 33 | Smith,  2020 (51) | Tobacco taxation, sales controls; warnings labels; standardised packs; retailer bans, (near schools, by pharmacies); smoke-free policies, marketing controls, | Inequalities in smoking | Apparently independent |  |
| 34 | Unal,  2021 (52) | Prohibition of cigarette adverts & sponsorship; age limitation on the sale of cigarettes, taxes, smoke-free workplaces; stimulant writing and pictures on cigarette packs | Smoking prevalence | Apparently independent |  |
| 35 | Wilson,  2012 (53) | Tobacco prices/ taxation; smoking bans in public places; health warning labels and bans on advertising and sponsorship | Smoking behaviour | Apparently independent |  |

**Gambling**

| **Number** | **Lead Author, Year** | **Intervention** | **Outcome** | **Industry involvement assessment** | |
| --- | --- | --- | --- | --- | --- |
|  |  |  |  | **Rating** | **Rationale** |
| 1 | Beckett,  2020 (54) | Staff training programs in land-based gambling venues | Gambling behaviour | Declared study funding from gambling industry | Project funded by ClubsNSW |
| 2 | Bjørseth,  2021 (55) | Pop-up messages | Gambling behaviour | Declared ongoing researcher consulting for gambling industry | MG regularly undertakes consultancy for various gaming companies in the area of social responsibility in gambling. |
| 3 | Drawson,  2017 (56) | Player Information Display System | Gambling behaviour | Declared study funding from industry linked organisations | Study funded by GREO: a gambling linked organisation |
| 4 | Forsström, 2021 (57) | Pop-up messages | Gambling behaviour | Undeclared previous researcher funding from gambling industry | Forstrom's PhD (2013-2017) was funded by Svenska spel, a Swedish gambling organisation. (this review was submitted in 2019, accepted in 2020) |
| 5 | Harris,  2017 (58) | Pop-up messages | Gambling behaviour | Declared current researcher employment in gambling industry-linked organisations | The Responsible Gambling Trust® (now GambleAware®). |
| 6 | Ladouceur,  2017 (59) | Venue staff responding to patron problem gambling; specific game/machine features | Gambling behaviour | Declared study funding from gambling industry sources | "During the preparation of this article Ladouceur received funding support for the International Group on Responsible Gambling from La Loterie Romande (Switzerland), ClubNSW (Australia), Camelot (United Kingdom), La Franc¸aise des Jeux (France), Loto Quebec (Quebec, Canada), the National Lottery (Belgium)" |
| 7 | Livingstone, 2014 (60) | The provision of responsible gambling via electronic gambling machines screens; Identification & interaction with gamblers; Removal of Automatic Teller Machines from venues; reduction in maximum bets. | Gambling behaviour | Un-declared previous researcher funding from gambling industry linked sources | From another publication: CL has received funding from Victorian Gambling Research Panel, the South Australian Independent Gambling Authority, and the Ontario Problem Gambling Research Council. These organisations receive funds from the proceeds of gambling |
| 8 | McAuliffe, 2021 (61) | Game-based structural features - pop-up messages, breaks in play, & covert structural tools; restricting the supply of EGMs; restricting EGM features - eg bank note acceptors removed. | Gambling behaviour | Declared study funding from gambling industry sources | Funded by GVC holdings (now Entain), an international gambling operator |
| 9 | Meyer,  2018 (62) | Bans; reducing machine numbers; age limits; limits on legal gambling forms | Gambling behaviour | Declared previous researcher funding from the gambling industry | Hayer has received grants from lottery providers |
| 10 | Rodda, 2022(63) | Restriction of access to money (e.g. removal of cash machines from gambling venues, ban on credit betting) | Gambling behaviour | Declared study funding from gambling industry linked organisations | Funded by GREO - an industry-linked organisation |
| 11 | Tanner,  2017 (64) | Pop-up messages; Cash-out/Winning Regulations; Max. Bets/Max. Line; Removal of Large Note Acceptors/ ATMs; Shutdown/Operating Hr; EGM caps; smoking bans | Gambling behaviour | Declared study funding from gambling industry linked sources | Funded by GREO - an industry-linked organisation |
| 12 | Vasiliadis,  2013 (65) | Reduction of gaming machine numbers and hours of access | Gambling behaviour | Un-declared previous researcher funding from sources linked to the gambling industry | From 2016 publication "The Problem Gambling Research and Treatment Centre at the University of Melbourne, with which several co-authors were affiliated when the data were collected, was previously supported by the Victorian Responsible Gambling Foundation"; |
| 13 | Whiteside, 2020 (66) | Legislation stipulating that half of any welfare payment could only be spent on certain products thus reducing the cash flow available for EGM gambling | Gambling behaviour | Declared study funding from Industry linked organisations | Review financially supported by Victorian Responsible Gambling Foundation |
| 14 | Zoglauer,  2021 (67) | Bans leading to removal of slot machines | Gambling behaviour | Declared previous researcher funding from the gambling industry | Roxana Kotter was funded by an unrestricted research donation by the Federal Association of German Casinos (BupriS) |

**Ultra-processed foods**

| **Number** | **Lead Author, Year** | **Intervention** | **Outcome** | **Industry involvement assessment** | |
| --- | --- | --- | --- | --- | --- |
|  |  |  |  | **Rating** | **Rationale** |
| 1 | Afshin,  2017 (68) | Price increases | Consumption, BMI, adiposity | Declared researcher funding from the (food) industry | DM reports ad hoc honoraria from Quaker Oats, ad hoc consulting fees from Foodminds, Nutrition Impact & membership of Unilever North America Scientific Advisory Board; research grants from GlaxoSmithKline |
| 2 | Alagiyawanna, 2015 (69) | Taxes | Consumption | Apparently independent |  |
| 3 | An,  2021 (70) | Front-of-package nutrition labelling (e.g. traffic light) | Purchases | Apparently independent |  |
| 4 | An,  2021 (71) | Sugar-Sweetened Beverage warning labels | Purchase, weight outcomes | Not enough information to rate |  |
| 5 | Anastasiou, 2019 (72) | Traffic light labels, serving size, nutrition panel labels | Intake | Apparently independent |  |
| 6 | Atanasova, 2022 (73) | Calorie labelling on menus; placing unhealthy snacks further away; calorie/ traffic light labelling; front of pack labelling; Health warnings on sugar-sweetened beverages | Consumption, BMI | Declared researcher funding from food industry | GF declare grants from Nestle outside the submitted work |
| 7 | Backholer, 2016 (74) | Tax on sugar-sweetened beverages | Purchases or consumption, BMI | Apparently independent |  |
| 8 | Bleich,  2017 (75) | Calorie labelling | Calories purchased, or consumed | Undeclared funder links to the food industry | This work was partially supported by ChildObesity180 whose funders include the Walmart Foundation |
| 9 | Bonab,  2020 (76) | Processed food reformulation; revising food standards -sugar & calorie reduction | Calorie intake and weight modification | Apparently independent |  |
| 10 | Boyland,  2022 (77) | Food and Non-alcoholic Beverage Marketing restrictions | Dietary intake, purchasing | Apparently independent |  |
| 11 | Brown,  2018 (78) | Food labels (traffic light, calories, fat, guideline daily amounts) | Calories consumed, | Apparently independent |  |
| 12 | Bucher,  2016 (79) | Positional changes of food placement | Product sales, food consumption | Apparently independent |  |
| 13 | Bucher,  2016 (80) | Serving sizes labelling | Intake/ consumption | Declared study food industry funding | This study was funded by a grant from Nestec Ltd. Two authors [KVDH and DL] are employed at Nestec Ltd. |
| 14 | Cabrera Escobar, 2013 (81) | Taxes, price rises for sugar-sweetened beverages | Consumption, obesity, overweight, body mass index | Apparently independent |  |
| 15 | Calancie,  2015 (82) | School menu labelling; school banning high-fat and high-sugar snack foods; smaller portion size in public service venues; limit advertisements of less healthy foods and beverages | Purchasing, BMI | Not enough information to rate |  |
| 16 | Cameron,  2016 (83) | Traffic–light nutrition labelling, summary scores based on food nutrient content | Purchasing, consumption or weight | Declared researcher links to the food industry | The authors are currently working with a supermarket chain (Champions IGA) in a publicly funded collaborative project to test a range of healthy eating interventions. |
| 17 | Carins,  2021 (84) | Menu labelling, calorie labelling, portion size offering, reformulation (to eliminate unhealthy fats and/or to reduce fat (PH responsibility deal) | Purchasing, consumption | Not enough information to rate |  |
| 18 | Carter,  2018 (1) | Information-based cues at point of choice (signs, banners, menu boards, posters) | Selection or consumption | Apparently independent |  |
| 19 | Cecchini,  2016 (85) | Front of packaging food labelling (traffic light, serving size, green red tick) | Product selection, calorie intake | Apparently independent |  |
| 20 | Chambers,  2015 (86) | Statutory or self-regulation of food marketing | Purchasing | Not enough information to rate |  |
| 21 | Chan,  2021 (87) | Calorie, nutrition labelling, | Food purchasing, intake | Apparently independent |  |
| 22 | Chu,  2021 (88) | Packaging (packaging size, partitioning, re-sealability, use of images) | Actual, intended consumption | Apparently independent |  |
| 23 | Clarke,  2020 (89) | Health warning labels for alcohol, food products high in sugar | Selection or consumption | Apparently independent |  |
| 24 | Crockett,  2018 (90) | Labelling on menus; nutritional labelling placed on, or adjacent to, a range of foods or drinks | Selection or consumption | Declared researcher links to the food industry | SJ: Chaired the Public Health Responsibility Deal Food Network (2011-2015) |
| 25 | Croker,  2020 (91) | Front of pack nutritional labelling/ serving size/ calorie content | Selection or consumption | Apparently independent |  |
| 26 | Daley,  2020 (92) | Physical activity calorie equivalent (PACE) food labelling | Selection or consumption | Apparently independent |  |
| 27 | Danielli,  2021 (93) | Restricting unhealthy food provision & advertising around schools; city-wide unhealthy food advertising bans on public transport; ban of unhealthy food & drinks sponsorship of sports events; mandated calorie labelling at fast food restaurant; banning new fast-food establishments; restricting unhealthy foods around schools; removing SSB from children's menus | Obesity, weight, BMI | Apparently independent |  |
| 28 | Downs,  2013 (94) | Mandatory trans-fatty acid ban; trans-fatty acid labelling; voluntary limits; | Trans fatty acid levels in people’s diet | Not enough information to rate |  |
| 29 | Fergus,  2021 (95) | Removal of sugar-sweetened beverages; substitution of sugary confectionary with healthy snacking; signs near the beverage cooler with calorie information | Purchases, nutrition related outcomes | Apparently independent |  |
| 30 | Fernandes, 2016 (96) | Labelling (eg calories, nutrient content, traffic-light) displayed at points of selection (eg menus, table displays, or menu boards, at buffets) | Calories ordered, food choices | Apparently independent |  |
| 31 | Gittelsohn, 2017 (97) | Sugar-sweetened beverage tax/ excise; high fat food tax | Purchasing, consumption | Not enough information to rate |  |
| 32 | Gittelsohn, 2012 (98) | Reduce the availability of unhealthy foods; healthy retailer initiatives; in store signage (shelf labels, posters), shop staff training, moved unhealthy products away from point of purchase | Obesity | Not enough information to rate |  |
| 33 | Golding,  2021 (99) | Product changes at checkout; sugary confectionery replaced with healthy snacks | Purchases, nutrition related outcomes | Declared researcher links to the food industry | Some members of the review team (AB, LP, SG, TC) have liaised with major food retailers in the UK on other public health projects |
| 34 | Grech,  2015 (100) | Traffic light labelling; calorie labelling; Decreasing sugar-sweetened beverage content of vending machines | Sales data, dietary intake or weight change | Apparently independent |  |
| 35 | Gressier,  2021 (101) | Reformulation (including trans fatty acids); Bans and voluntary reformulation | Purchases, sales, BMI, | Declared researcher funding direct from industry | MG is the recipient of a PhD Studentship grant from Nestec. |
| 36 | Grummon, 2020 (102) | Sugary drink warnings (displayed on products’ front of package, on menus, or at point of sale) | Consumption | Apparently independent |  |
| 37 | Gupta,  2021 (103) | Sugary drink front of package labels; Sugary drink point of sale signage | Purchase, consumption | Apparently independent |  |
| 38 | Hashem,  2019 (104) | Product reformulation to reduce sugar content | Intake, body weight | Apparently independent |  |
| 39 | Hendry,  2015 (105) | Trans fatty acid controls (maximum limits on the use of artificial TFAs; mandated explicit labelling of artificial TFAs) | Obesity, purchasing consumption | Not enough information to rate |  |
| 40 | Hersey,  2013 (106) | Front-of-package (eg traffic light, GDA) and shelf nutrition labelling | Consumer response | Apparently independent |  |
| 41 | Hillier-Brown, 2017 (107) | Trans fat law, Price increases for unhealthy choices; Changing pre-packed children’s meal content; smaller portion size option; signposting (unhealthy, healthy symbols on menus); calorie labelling law; voluntary calorie labelling; outlet award schemes. | Dietary outcomes (e.g. energy intake), purchasing behaviour | Apparently independent |  |
| 42 | Hollands,  2015 (34) | Portion size; Package size; Tableware size or shape | Selection or consumption | Declared researcher links to the food industry | SJ is Chair of the Public Health Responsibility Deal Food Network, which develops voluntary agreements with industry. She was also a co-author of a study (completed 2010) funded by the Coca-Cola Institute for Health & Wellness |
| 43 | Hollands,  2019 (108) | Reducing numbers of snacks/ drinks available; making snacks/ drinks less proximal | Selection or consumption | Apparently independent |  |
| 44 | Karpyn,  2020 (109) | Changes to endcap food displays ; point-of-sale nutritional information; calorie labelling intervention | Consumption or purchasing. | Apparently independent |  |
| 45 | Lhachimi,  2020 (110) | Tax on saturated fat content | Consumption, energy intake, overweight or obesity | Apparently independent |  |
| 46 | Littlewood, 2016 (111) | Menu labelling (e.g. energy) | Purchasing, energy consumed | Apparently independent |  |
| 47 | Liu,  2022 (112) | Reducing the package size of Energy Dense Nutrient Poor snacks and drinks | Consumption | Apparently independent |  |
| 48 | Long,  2015 (113) | Menu energy labelling | Calories ordered or purchased | Not enough information to rate |  |
| 49 | Mah,  2019 (114) | Taxes; shelf labelling requirements | Purchasing, dietary intakes, weight | Apparently independent |  |
| 50 | Mandracchia, 2021 (115) | Menu with limited unhealthy options (including portion size, nutrient-content limitation); Staff training; Point-of-purchase food positioning; Packaging: Prompts; Menu inserts & symbols; Incentives/ rewards for restaurants & canteens; Food labelling (i.e traffic lights) | Dietary intake, food availability, purchasing | Apparently independent |  |
| 51 | Maniadakis, 2013 (116) | Taxes | Consumption, caloric intake, weight outcomes | Not enough information to rate |  |
| 52 | Mayne,  2015 (117) | Trans fat ban; nutrition labelling in restaurants, sugar-sweetened beverage bans in school vending machines/cafes; nutrition labelling in supermarkets (point of choice) | body mass index, weight, diet | Apparently independent |  |
| 53 | McGill,  2015 (118) | Tax on high energy density foods/ sugar-sweetened beverages /less nutritionally healthy food/ saturated fats | Dietary intake, change in BMI | Apparently independent |  |
| 54 | Metcalfe,  2020 (119) | Nutrition / calorie labelling at point of school meal sale; changing product choice, changing product availability; changing product placement | Selection, consumption, | Apparently independent |  |
| 55 | Micha,  2018 (120) | Competitive food/beverage standards in schools | Dietary habits, adiposity | Declared researcher links to the food industry | Micha is PI of a research grant from Unilever on an investigator initiated project; Mozaffarian reports personal fees from Haas Avocado Board, Pollock Communications, |
| 56 | Molina,  2022 (121) | Taxes on sugar-sweetened beverages; marketing regulations on sugar sweetened products; food labelling | purchases, pricing, formulation | Apparently independent |  |
| 57 | Nakhimovsky, 2016 (122) | Taxes on sugar-sweetened beverages | Consumption, adiposity | Apparently independent |  |
| 58 | Olstad,  2016 (123) | Tax on unhealthy foods; menu labelling; soft drink taxes; grocery & vending machine soda taxes | Impact on inequalities | Apparently independent |  |
| 59 | Pereira,  2021 (124) | Sugar-sweetened beverage taxation; food labelling | BMI | Apparently independent |  |
| 60 | Pfinder,  2020 (125) | Taxation of sugary foods | Consumption, energy intake, overweight or obesity | Apparently independent |  |
| 61 | Powell,  2013 (126) | Soda taxes, fast food taxes | Consumption or weight outcomes | Not enough information to rate |  |
| 62 | Redondo,  2018 (127) | Sugar-sweetened beverage taxes | consumption, sales, or purchase | Not enough information to rate |  |
| 63 | Roberts,  2017 (128) | Fiscal measures targeted at high sugar foods & sugar-sweetened drinks (sales tax/ excise tax) | Consumption, purchasing, weight, | Apparently independent |  |
| 64 | Roy,  2015 (129) | Nutrition labelling / posters (energy, serving size, fat, content) | Food choices, purchases | Apparently independent |  |
| 65 | Sacco.  2017 (130) | Menu labelling (calorie labelling, nutrition facts, traffic lights) | Calories ordered | Apparently independent |  |
| 66 | Sarink,  2016 (131) | Menu labelling (calories) | Calories purchased | Apparently independent |  |
| 67 | Scapin,  2021 (132) | Sugar content labels | Food choices | Apparently independent |  |
| 68 | Seyedhamzeh, 2018 (133) | Physical activity equivalent labelling vs. calorie labelling | Calories ordered | Apparently independent |  |
| 69 | Shangguan, 2019 (134) | Menu labelling; Package labelling; Point-of-purchase labelling | Consumer behaviors, health outcomes | Declared researcher links to the food, weight loss industry | Mozaffarian: personal fees from Nutrition Impact, Pollock Communications, Indigo Agriculture, & America’s Test Kitchen; Micha reports funding from Unilever |
| 70 | Shaw,  2020 (135) | Removal of unhealthy foods at checkout/ eye level | Dietary intake, food sales data, body composition | Declared researcher links to the food industry | J.B. & C.A.V. have a nonfinancial research collaboration with a UK supermarket chain. J.B. has received grant research support from Danone Early Life Nutrition |
| 71 | Silden,  2018 (136) | Competitive food (a la carte, vending, school stores) policies | BMI, caloric intake | Apparently independent |  |
| 72 | Silva,  2021 (137) | Front-of-package warning label for foods high in sugar, sodium, saturated fat and calories | Purchasing | Declared study industry funding | This research was funded by ALAIAB, Alianza Latinoamericana de Asociaciones de la Industria de Alimentos y Bebidas. |
| 73 | Sinclair,  2014 (138) | Menu labelling (calories, nutritional information) | Selection,  consumption of calories | Apparently independent |  |
| 74 | Singh,  2017 (139) | Sugar-sweetened beverage/ junk food ban in schools; removal of low nutritional value snacks | BMI | Apparently independent |  |
| 75 | Skov,  2013 (140) | Choice architecture interventions (portion size; cutlery; table wear size; calorie labelling; variety of products on offer) | Health or food consumption-related | Apparently independent |  |
| 76 | Taillie,  2020 (141) | Front-of-Package Nutrient Warning Labels on Sugar-Sweetened Beverages & Ultra-Processed Foods (sugar, saturated fat, total fat, calories) | Intended consumption | Apparently independent |  |
| 77 | Teng,  2019 (142) | Consumer taxes on sugar-sweetened beverages | Consumption | Apparently independent |  |
| 78 | Thow,  2014 (143) | Taxes on fat, sugar, and salt/ unhealthy food | Consumption | Not enough information to rate |  |
| 79 | Tseng,  2018 (144) | Zoning regulation restricting opening/expanding of standalone fast-food restaurants; calorie labelling on menus | Weight, BMI | Declared researcher links to the weight loss industry | LC served on the scientific advisory board of Medifast during the conduct of the study. |
| 80 | Van der Horst, 2019 (145) | Serving Size Information on Food Labels | Consumption | Declared study industry funding | This research was funded by Société des Produits Nestlé |
| 81 | Van 't Riet,  2013 (146) | Product health information on shelf tags, posters, brochures, flyers; package labels | Purchasing behaviour | Apparently independent |  |
| 82 | Vargas-Alvarez, 2021 (147) | Glassware, plate size, portion control plates, differently sized tableware, cutlery, serving utensils | Intake, purchases, weight | Declared researcher previous links to the food industry | E.A.-R. has received a donation of portion control tools for research from Precise Portions NLS in the past. |
| 83 | von Philipsborn, 2019 (148) | Traffic-light labelling of sugar-sweetened beverages; menu-board calorie labelling of SSB; reduced availability of SSBs in schools; price increases on SSBs; healthier default beverages in children’s menus in chain restaurant; urban planning restrictions on new fast-food restaurants; restrictions on the number of stores selling SSBs; | Measures of intake, diet-related anthropometric measures | Declared researcher links to the food, weight loss industry | HH received honoraria for scientific advisory boards membership including Weight Watchers, Nestle and Danone |
| 84 | Von Philipsborn, 2020 (149) | Traffic-light labelling of sugar-sweetened beverages; menu-board calorie labelling of SSB; reduced availability of SSBs in schools; price increases on SSBs; healthier default beverages in children’s menus in chain restaurant; urban planning restrictions on new fast-food restaurants; restrictions on the number of stores selling SSBs; | Consumption, anthropometry | Declared researcher funding from the food industry | HH received honoraria for scientific advisory boards membership including Weight Watchers, Nestle and Danone |
| 85 | Wethington, 2020 (150) | Changes to competitive foods & beverages policies to meet established nutritional standards or guidelines (foods sold in competition with school meal programs, & include à la carte foods, vending machines, school stores & snack bars) | Consumption, weight changes | Not enough information to rate |  |
| 86 | Whatnall, 2020 (151) | Price alterations; modifying product placement within vending machines; traffic light labelling; posters next to vending machines | Purchase/sales, dietary behavior change, | Apparently independent |  |
| 87 | Wilson,  2016 (152) | Choice architecture interventions (visibility, accessibility- serving equipment, availability - choice of products); Calorie labels; traffic light labels on products | Consumption, selection, purchasing | Not enough information to rate |  |
| 88 | Wright,  2017 (153) | Taxes on retailers and manufacturers of unhealthy products; Consumer taxes targeting unhealthy foods | Consumption | Apparently independent |  |
| 89 | Wyse,  2021 (154) | Choice architecture strategies in online food ordering systems (applying labels in the online menus, product lists; changing choice defaults) | Purchases | Apparently independent |  |

1. Carter P, Bignardi G, Hollands GJ, Marteau TM. Information-based cues at point of choice to change selection and consumption of food, alcohol and tobacco products: a systematic review. BMC Public Health. 2018;18(1):418.

2. Gmel G, Holmes J, Studer J. Are alcohol outlet densities strongly associated with alcohol-related outcomes? A critical review of recent evidence. Drug Alcohol Rev. 2016;35(1):40-54.

3. Hassan LM, Shiu E. A systematic review of the efficacy of alcohol warning labels. Journal of Social Marketing. 2018;8(3):333-52.

4. Kingsland M, Wiggers JH, Vashum KP, Hodder RK, Wolfenden L. Interventions in sports settings to reduce risky alcohol consumption and alcohol-related harm: a systematic review. Syst Rev. 2016;5:12.

5. Kokole D, Anderson P, Jane-Llopis E. Nature and Potential Impact of Alcohol Health Warning Labels: A Scoping Review. Nutrients. 2021;13(9).

6. Li Q, Babor TF, Zeigler D, Xuan Z, Morisky D, Hovell MF, et al. Health promotion interventions and policies addressing excessive alcohol use: a systematic review of national and global evidence as a guide to health-care reform in China. Addiction. 2015;110 Suppl 1(0 1):68-78.

7. Nelson JP. Binge drinking and alcohol prices: a systematic review of age-related results from econometric studies, natural experiments and field studies. Health Econ Rev. 2015;5:6.

8. Nelson JP, McNall AD. What happens to drinking when alcohol policy changes? A review of five natural experiments for alcohol taxes, prices, and availability. Eur J Health Econ. 2017;18(4):417-34.

9. Nepal S, Kypri K, Pursey K, Attia J, Chikritzhs T, Miller P. Effectiveness of lockouts in reducing alcohol-related harm: Systematic review. Drug Alcohol Rev. 2018;37(4):527-36.

10. Robinson E, Humphreys G, Jones A. Alcohol, calories, and obesity: A rapid systematic review and meta-analysis of consumer knowledge, support, and behavioral effects of energy labeling on alcoholic drinks. Obes Rev. 2021;22(6):e13198.

11. Sanchez-Ramirez DC, Voaklander D. The impact of policies regulating alcohol trading hours and days on specific alcohol-related harms: a systematic review. Inj Prev. 2018;24(1):94-100.

12. Scholes-Balog KE, Heerde JA, Hemphill SA. Alcohol warning labels: unlikely to affect alcohol-related beliefs and behaviours in adolescents. Aust N Z J Public Health. 2012;36(6):524-9.

13. Siegfried N, Pienaar DC, Ataguba JE, Volmink J, Kredo T, Jere M, et al. Restricting or banning alcohol advertising to reduce alcohol consumption in adults and adolescents. Cochrane Database Syst Rev. 2014;2014(11):CD010704.

14. Sileo KM, Miller AP, Huynh TA, Kiene SM. A systematic review of interventions for reducing heavy episodic drinking in sub-Saharan African settings. PLoS One. 2020;15(12):e0242678.

15. Stockings E, Bartlem K, Hall A, Hodder R, Gilligan C, Wiggers J, et al. Whole-of-community interventions to reduce population-level harms arising from alcohol and other drug use: a systematic review and meta-analysis. Addiction. 2018;113(11):1984-2018.

16. Vashishtha R, Livingston M, Pennay A, Dietze P, MacLean S, Holmes J, et al. Why is adolescent drinking declining? A systematic review and narrative synthesis. Addiction Research & Theory. 2020;28(4):275-88.

17. Wilkinson C, Livingston M, Room R. Impacts of changes to trading hours of liquor licences on alcohol-related harm: a systematic review 2005-2015. Public Health Res Pract. 2016;26(4).

18. Wilson IM, Graham K, Taft A. Alcohol interventions, alcohol policy and intimate partner violence: a systematic review. BMC Public Health. 2014;14:881.

19. Bafunno D, Catino A, Lamorgese V, Pizzutilo P, Di Lauro A, Petrillo P, et al. Tobacco control in Europe: A review of campaign strategies for teenagers and adults. Crit Rev Oncol Hematol. 2019;138:139-47.

20. Bafunno D, Catino A, Lamorgese V, Del Bene G, Longo V, Montrone M, et al. Impact of tobacco control interventions on smoking initiation, cessation, and prevalence: a systematic review. J Thorac Dis. 2020;12(7):3844-56.

21. Bardus M, El Boukhari N, Nakkash R. Development and evaluation of smoke-free or tobacco-free policies in university settings: a systematic scoping review. Health Educ Res. 2020;35(4):306-51.

22. Bennett BL, Deiner M, Pokhrel P. College anti-smoking policies and student smoking behavior: a review of the literature. Tob Induc Dis. 2017;15:11.

23. Berman ML, Glasser AM. Nicotine Reduction in Cigarettes: Literature Review and Gap Analysis. Nicotine Tob Res. 2019;21(Suppl 1):S133-S44.

24. Bird Y, Kashaniamin L, Nwankwo C, Moraros J. Impact and Effectiveness of Legislative Smoking Bans and Anti-Tobacco Media Campaigns in Reducing Smoking among Women in the US: A Systematic Review and Meta-Analysis. Healthcare (Basel). 2020;8(1).

25. Brown T, Platt S, Amos A. Equity impact of population-level interventions and policies to reduce smoking in adults: a systematic review. Drug Alcohol Depend. 2014;138:7-16.

26. Brown T, Platt S, Amos A. Equity impact of interventions and policies to reduce smoking in youth: systematic review. Tob Control. 2014;23(e2):e98-105.

27. Cadham CJ, Sanchez-Romero LM, Fleischer NL, Mistry R, Hirschtick JL, Meza R, et al. The actual and anticipated effects of a menthol cigarette ban: a scoping review. BMC Public Health. 2020;20(1):1055.

28. Drovandi A, Teague PA, Glass B, Malau-Aduli B. A systematic review of the perceptions of adolescents on graphic health warnings and plain packaging of cigarettes. Syst Rev. 2019;8(1):25.

29. Forberger S, Khan Z, Ahmad F, Ahmed F, Frense J, Kampfmann T, et al. Scoping Review of Existing Evaluations of Smokeless Tobacco Control Policies: What Is Known About Countries Covered, Level of Jurisdictions, Target Groups Studied, and Instruments Evaluated? Nicotine Tob Res. 2022;24(9):1344-54.

30. Frazer K, Callinan JE, McHugh J, van Baarsel S, Clarke A, Doherty K, et al. Legislative smoking bans for reducing harms from secondhand smoke exposure, smoking prevalence and tobacco consumption. Cochrane Database Syst Rev. 2016;2(2):CD005992.

31. Frazer K, McHugh J, Callinan JE, Kelleher C. Impact of institutional smoking bans on reducing harms and secondhand smoke exposure. Cochrane Database Syst Rev. 2016;2016(5):CD011856.

32. Galanti MR, Coppo A, Jonsson E, Bremberg S, Faggiano F. Anti-tobacco policy in schools: upcoming preventive strategy or prevention myth? A review of 31 studies. Tob Control. 2014;23(4):295-301.

33. Garritsen HH, da Costa Senior YY, Rozema AD, Kunst AE, Kuipers MAG. Association Between Smoke-Free Legislation in Hospitality Venues and Smoking Behavior of Young People: A Systematic Review. Nicotine Tob Res. 2022;24(6):807-12.

34. Hollands GJ, Shemilt I, Marteau TM, Jebb SA, Lewis HB, Wei Y, et al. Portion, package or tableware size for changing selection and consumption of food, alcohol and tobacco. Cochrane Database Syst Rev. 2015;2015(9):CD011045.

35. Jawad M, Jawad S, Waziry RK, Ballout RA, Akl EA. Interventions for waterpipe tobacco smoking prevention and cessation: a systematic review. Sci Rep. 2016;6:25872.

36. Jetly K, Ismail A, Hassan N, Mohammed Nawi A. Perceived Influence of Plain Cigarette Packaging on Smoking Behavior: A Systematic Review. J Public Health Manag Pract. 2022;28(5):E757-E63.

37. Kienhuis AS, Talhout R. Options for waterpipe product regulation: A systematic review on product characteristics that affect attractiveness, addictiveness and toxicity of waterpipe use. Tob Induc Dis. 2020;18:69.

38. McKay AJ, Patel RK, Majeed A. Strategies for tobacco control in India: a systematic review. PLoS One. 2015;10(4):e0122610.

39. McNeill A, Gravely S, Hitchman SC, Bauld L, Hammond D, Hartmann-Boyce J. Tobacco packaging design for reducing tobacco use. Cochrane Database Syst Rev. 2017;4(4):CD011244.

40. Minichiello A, Lefkowitz AR, Firestone M, Smylie JK, Schwartz R. Effective strategies to reduce commercial tobacco use in Indigenous communities globally: A systematic review. BMC Public Health. 2016;16:21.

41. Nanninga S, Lehne G, Ratz T, Bolte G. Impact of Public Smoking Bans on Social Inequalities in Children's Exposure to Tobacco Smoke at Home: An Equity-Focused Systematic Review. Nicotine Tob Res. 2019;21(11):1462-72.

42. Nazar GP, Sharma N, Chugh A, Abdullah SM, Lina S, Mdege ND, et al. Impact of tobacco price and taxation on affordability and consumption of tobacco products in the South-East Asia Region: A systematic review. Tob Induc Dis. 2021;19:97.

43. Noar SM, Francis DB, Bridges C, Sontag JM, Ribisl KM, Brewer NT. The impact of strengthening cigarette pack warnings: Systematic review of longitudinal observational studies. Soc Sci Med. 2016;164:118-29.

44. Nuyts PAW, Kuijpers TG, Willemsen MC, Kunst AE. How can a ban on tobacco sales to minors be effective in changing smoking behaviour among youth? - A realist review. Prev Med. 2018;115:61-7.

45. Rado MK, Molenberg FJM, Westenberg LEH, Sheikh A, Millett C, Burdorf A, et al. Effect of smoke-free policies in outdoor areas and private places on children's tobacco smoke exposure and respiratory health: a systematic review and meta-analysis. Lancet Public Health. 2021;6(8):e566-e78.

46. Rashiden I, Ahmad Tajuddin N, Yee A, Zhen STE, Bin Amir Nordin AS. The efficacy of smoking ban policy at the workplace on secondhand smoking: systematic review and meta-analysis. Environ Sci Pollut Res Int. 2020;27(24):29856-66.

47. Robertson L, McGee R, Marsh L, Hoek J. A systematic review on the impact of point-of-sale tobacco promotion on smoking. Nicotine Tob Res. 2015;17(1):2-17.

48. Rogers T, Brown EM, Siegel-Reamer L, Rahman B, Feld AL, Patel M, et al. A Comprehensive Qualitative Review of Studies Evaluating the Impact of Local US Laws Restricting the Sale of Flavored and Menthol Tobacco Products. Nicotine Tob Res. 2022;24(4):433-43.

49. Schreuders M, Nuyts PAW, van den Putte B, Kunst AE. Understanding the impact of school tobacco policies on adolescent smoking behaviour: A realist review. Soc Sci Med. 2017;183:19-27.

50. Shemilt I, Hendry V, Marteau TM. What do we know about the effects of exposure to 'Low alcohol' and equivalent product labelling on the amounts of alcohol, food and tobacco people select and consume? A systematic review. BMC Public Health. 2017;17(1):29.

51. Smith CE, Hill SE, Amos A. Impact of population tobacco control interventions on socioeconomic inequalities in smoking: a systematic review and appraisal of future research directions. Tob Control. 2020;30(e2):e87-95.

52. Unal E, Metintas S. Effectiveness of anti-smoking interventions towards community: a meta-analysis study. Cent Eur J Public Health. 2021;29(2):134-42.

53. Wilson LM, Avila Tang E, Chander G, Hutton HE, Odelola OA, Elf JL, et al. Impact of tobacco control interventions on smoking initiation, cessation, and prevalence: a systematic review. J Environ Public Health. 2012;2012:961724.

54. Beckett M, Keen B, Angus DJ, Pickering D, Blaszczynski A. Responsible gambling staff training in land-based venues: a systematic review. International Gambling Studies. 2020;20(2):331-67.

55. Bjorseth B, Simensen JO, Bjornethun A, Griffiths MD, Erevik EK, Leino T, et al. The Effects of Responsible Gambling Pop-Up Messages on Gambling Behaviors and Cognitions: A Systematic Review and Meta-Analysis. Front Psychiatry. 2020;11:601800.

56. Drawson AS, Tanner J, Mushquash CJ, Mushquash AR, Mazmanian D. The Use of Protective Behavioural Strategies in Gambling: a Systematic Review. International Journal of Mental Health and Addiction. 2017;15(6):1302-19.

57. Forsström D, Spångberg J, Petterson A, Brolund A, Odeberg J. A systematic review of educational programs and consumer protection measures for gambling: an extension of previous reviews. Addiction Research & Theory. 2021;29(5):398-412.

58. Harris A, Griffiths MD. A Critical Review of the Harm-Minimisation Tools Available for Electronic Gambling. J Gambl Stud. 2017;33(1):187-221.

59. Ladouceur R, Shaffer P, Blaszczynski A, Shaffer HJ. Responsible gambling: a synthesis of the empirical evidence. Addiction Research & Theory. 2017;25(3):225-35.

60. Livingstone C, Rintoul A, Francis L. What is the evidence for harm minimisation measures in gambling venues? Evidence Base. 2014(2).

61. McAuliffe WHB, Edson TC, Louderback ER, LaRaja A, LaPlante DA. Responsible product design to mitigate excessive gambling: A scoping review and z-curve analysis of replicability. PLoS One. 2021;16(4):e0249926.

62. Meyer G, Kalke J, Hayer T. The impact of supply reductionon the prevalence of gamblingparticipation and disordered gambling behavior: A systematic review. SUCHT. 2018;64(5-6):283-93.

63. Rodda SN. A scoping review of hard systems and tools that restrict money and cash for gambling. J Behav Addict. 2021;10(3):587-600.

64. Tanner J, Drawson AS, Mushquash CJ, Mushquash AR, Mazmanian D. Harm reduction in gambling: a systematic review of industry strategies. Addiction Research & Theory. 2017;25(6):485-94.

65. Vasiliadis SD, Jackson AC, Christensen D, Francis K. Physical accessibility of gaming opportunity and its relationship to gaming involvement and problem gambling: A systematic review. Journal of Gambling Issues. 2013;28:1-46.

66. Whiteside M, Heyeres M, Maltzahn K, Griffin T, MacLean S. Intervening in Indigenous Gambling: A Systematic Review of the Literature. SAGE Open. 2020;10(3):2158244020947441.

67. Zoglauer M, Czernecka R, Bühringer G, Kotter R, Kräplin A. The Relationship Between Physical Availability of Gambling and Gambling Behaviour or Gambling Disorder: A Systematic Review. Journal of Gambling Issues. 2021;27.

68. Afshin A, Penalvo JL, Del Gobbo L, Silva J, Michaelson M, O'Flaherty M, et al. The prospective impact of food pricing on improving dietary consumption: A systematic review and meta-analysis. PLoS One. 2017;12(3):e0172277.

69. Alagiyawanna A, Townsend N, Mytton O, Scarborough P, Roberts N, Rayner M. Studying the consumption and health outcomes of fiscal interventions (taxes and subsidies) on food and beverages in countries of different income classifications; a systematic review. BMC Public Health. 2015;15:887.

70. An R, Shi Y, Shen J, Bullard T, Liu G, Yang Q, et al. Effect of front-of-package nutrition labeling on food purchases: a systematic review. Public Health. 2021;191:59-67.

71. An R, Liu J, Liu R, Barker AR, Figueroa RB, McBride TD. Impact of Sugar-Sweetened Beverage Warning Labels on Consumer Behaviors: A Systematic Review and Meta-Analysis. Am J Prev Med. 2021;60(1):115-26.

72. Anastasiou K, Miller M, Dickinson K. The relationship between food label use and dietary intake in adults: A systematic review. Appetite. 2019;138:280-91.

73. Atanasova P, Kusuma D, Pineda E, Frost G, Sassi F, Miraldo M. The impact of the consumer and neighbourhood food environment on dietary intake and obesity-related outcomes: A systematic review of causal impact studies. Soc Sci Med. 2022;299:114879.

74. Backholer K, Sarink D, Beauchamp A, Keating C, Loh V, Ball K, et al. The impact of a tax on sugar-sweetened beverages according to socio-economic position: a systematic review of the evidence. Public Health Nutr. 2016;19(17):3070-84.

75. Bleich SN, Economos CD, Spiker ML, Vercammen KA, VanEpps EM, Block JP, et al. A Systematic Review of Calorie Labeling and Modified Calorie Labeling Interventions: Impact on Consumer and Restaurant Behavior. Obesity (Silver Spring). 2017;25(12):2018-44.

76. Milani Bonab A, Kalantari N, Zargaraan A, Haghighian Roudsari A, Pourmoradian S. Can Food Reformulation Policy Reduce Calorie Intake and Tackle Childhood Obesity? Iran J Pediatr. 2020;30(6):e98985.

77. Boyland E, McGale L, Maden M, Hounsome J, Boland A, Jones A. Systematic review of the effect of policies to restrict the marketing of foods and non-alcoholic beverages to which children are exposed. Obes Rev. 2022;23(8):e13447.

78. Brown HM, Rollo ME, de Vlieger NM, Collins CE, Bucher T. Influence of the nutrition and health information presented on food labels on portion size consumed: a systematic review. Nutr Rev. 2018;76(9):655-77.

79. Bucher T, Collins C, Rollo ME, McCaffrey TA, De Vlieger N, Van der Bend D, et al. Nudging consumers towards healthier choices: a systematic review of positional influences on food choice. Br J Nutr. 2016;115(12):2252-63.

80. Bucher T, Murawski B, Duncanson K, Labbe D, Van der Horst K. The effect of the labelled serving size on consumption: A systematic review. Appetite. 2018;128:50-7.

81. Cabrera Escobar MA, Veerman JL, Tollman SM, Bertram MY, Hofman KJ. Evidence that a tax on sugar sweetened beverages reduces the obesity rate: a meta-analysis. BMC Public Health. 2013;13:1072.

82. Calancie L, Leeman J, Jilcott Pitts SB, Khan LK, Fleischhacker S, Evenson KR, et al. Nutrition-related policy and environmental strategies to prevent obesity in rural communities: a systematic review of the literature, 2002-2013. Prev Chronic Dis. 2015;12:E57.

83. Cameron AJ, Charlton E, Ngan WW, Sacks G. A Systematic Review of the Effectiveness of Supermarket-Based Interventions Involving Product, Promotion, or Place on the Healthiness of Consumer Purchases. Current Nutrition Reports. 2016;5(3):129-38.

84. Carins J, Pang B, Willmott T, Knox K, Storr R, Robertson D, et al. Creating supportive eating places: a systematic review of food service initiatives. Health Promot Int. 2021;36(5):1368-92.

85. Cecchini M, Warin L. Impact of food labelling systems on food choices and eating behaviours: a systematic review and meta-analysis of randomized studies. Obes Rev. 2016;17(3):201-10.

86. Chambers SA, Freeman R, Anderson AS, MacGillivray S. Reducing the volume, exposure and negative impacts of advertising for foods high in fat, sugar and salt to children: A systematic review of the evidence from statutory and self-regulatory actions and educational measures. Prev Med. 2015;75:32-43.

87. Chan J, McMahon E, Brimblecombe J. Point-of-sale nutrition information interventions in food retail stores to promote healthier food purchase and intake: A systematic review. Obes Rev. 2021;22(10):e13311.

88. Chu R, Tang T, Hetherington MM. The impact of food packaging on measured food intake: A systematic review of experimental, field and naturalistic studies. Appetite. 2021;166:105579.

89. Clarke N, Pechey E, Kosite D, Konig LM, Mantzari E, Blackwell AKM, et al. Impact of health warning labels on selection and consumption of food and alcohol products: systematic review with meta-analysis. Health Psychol Rev. 2021;15(3):430-53.

90. Crockett RA, King SE, Marteau TM, Prevost AT, Bignardi G, Roberts NW, et al. Nutritional labelling for healthier food or non-alcoholic drink purchasing and consumption. Cochrane Database Syst Rev. 2018;2(2):CD009315.

91. Croker H, Packer J, Russell SJ, Stansfield C, Viner RM. Front of pack nutritional labelling schemes: a systematic review and meta-analysis of recent evidence relating to objectively measured consumption and purchasing. J Hum Nutr Diet. 2020;33(4):518-37.

92. Daley AJ, McGee E, Bayliss S, Coombe A, Parretti HM. Effects of physical activity calorie equivalent food labelling to reduce food selection and consumption: systematic review and meta-analysis of randomised controlled studies. J Epidemiol Community Health. 2020;74(3):269-75.

93. Danielli S, Coffey T, Ashrafian H, Darzi A. Systematic review into city interventions to address obesity. EClinicalMedicine. 2021;32:100710.

94. Downs SM, Thow AM, Leeder SR. The effectiveness of policies for reducing dietary trans fat: a systematic review of the evidence. Bull World Health Organ. 2013;91(4):262-9H.

95. Fergus L, Seals K, Holston D. Nutrition Interventions in Low-Income Rural and Urban Retail Environments: A Systematic Review. J Acad Nutr Diet. 2021;121(6):1087-114.

96. Fernandes AC, Oliveira RC, Proenca RP, Curioni CC, Rodrigues VM, Fiates GM. Influence of menu labeling on food choices in real-life settings: a systematic review. Nutr Rev. 2016;74(8):534-48.

97. Gittelsohn J, Trude ACB, Kim H. Pricing Strategies to Encourage Availability, Purchase, and Consumption of Healthy Foods and Beverages: A Systematic Review. Prev Chronic Dis. 2017;14:E107.

98. Gittelsohn J, Rowan M, Gadhoke P. Interventions in small food stores to change the food environment, improve diet, and reduce risk of chronic disease. Prev Chronic Dis. 2012;9:E59.

99. Golding SE, Bondaronek P, Bunten AK, Porter L, Maynard V, Rennie D, et al. Interventions to change purchasing behaviour in supermarkets: a systematic review and intervention content analysis. Health Psychol Rev. 2022;16(2):305-45.

100. Grech A, Allman-Farinelli M. A systematic literature review of nutrition interventions in vending machines that encourage consumers to make healthier choices. Obes Rev. 2015;16(12):1030-41.

101. Gressier M, Swinburn B, Frost G, Segal AB, Sassi F. What is the impact of food reformulation on individuals' behaviour, nutrient intakes and health status? A systematic review of empirical evidence. Obes Rev. 2021;22(2):e13139.

102. Grummon AH, Hall MG. Sugary drink warnings: A meta-analysis of experimental studies. PLoS Med. 2020;17(5):e1003120.

103. Gupta A, Billich N, George NA, Blake MR, Huse O, Backholer K, et al. The effect of front-of-package labels or point-of-sale signage on consumer knowledge, attitudes and behavior regarding sugar-sweetened beverages: a systematic review. Nutr Rev. 2021;79(10):1165-81.

104. Hashem KM, He FJ, MacGregor GA. Effects of product reformulation on sugar intake and health-a systematic review and meta-analysis. Nutr Rev. 2019;77(3):181-96.

105. Hendry VL, Almiron-Roig E, Monsivais P, Jebb SA, Neelon SE, Griffin SJ, et al. Impact of regulatory interventions to reduce intake of artificial trans-fatty acids: a systematic review. Am J Public Health. 2015;105(3):e32-42.

106. Hersey JC, Wohlgenant KC, Arsenault JE, Kosa KM, Muth MK. Effects of front-of-package and shelf nutrition labeling systems on consumers. Nutr Rev. 2013;71(1):1-14.

107. Hillier-Brown FC, Summerbell CD, Moore HJ, Routen A, Lake AA, Adams J, et al. The impact of interventions to promote healthier ready-to-eat meals (to eat in, to take away or to be delivered) sold by specific food outlets open to the general public: a systematic review. Obes Rev. 2017;18(2):227-46.

108. Hollands GJ, Carter P, Anwer S, King SE, Jebb SA, Ogilvie D, et al. Altering the availability or proximity of food, alcohol, and tobacco products to change their selection and consumption. Cochrane Database Syst Rev. 2019;9(9):CD012573.

109. Karpyn A, McCallops K, Wolgast H, Glanz K. Improving Consumption and Purchases of Healthier Foods in Retail Environments: A Systematic Review. Int J Environ Res Public Health. 2020;17(20).

110. Lhachimi SK, Pega F, Heise TL, Fenton C, Gartlehner G, Griebler U, et al. Taxation of the fat content of foods for reducing their consumption and preventing obesity or other adverse health outcomes. Cochrane Database Syst Rev. 2020;9(9):Cd012415.

111. Littlewood JA, Lourenço S, Iversen CL, Hansen GL. Menu labelling is effective in reducing energy ordered and consumed: a systematic review and meta-analysis of recent studies. Public Health Nutr. 2016;19(12):2106-21.

112. Liu Q, Tam LY, Rangan A. The Effect of Downsizing Packages of Energy-Dense, Nutrient-Poor Snacks and Drinks on Consumption, Intentions, and Perceptions-A Scoping Review. Nutrients. 2021;14(1).

113. Long MW, Tobias DK, Cradock AL, Batchelder H, Gortmaker SL. Systematic review and meta-analysis of the impact of restaurant menu calorie labeling. Am J Public Health. 2015;105(5):e11-24.

114. Mah CL, Luongo G, Hasdell R, Taylor NGA, Lo BK. A Systematic Review of the Effect of Retail Food Environment Interventions on Diet and Health with a Focus on the Enabling Role of Public Policies. Curr Nutr Rep. 2019;8(4):411-28.

115. Mandracchia F, Tarro L, Llaurado E, Valls RM, Sola R. Interventions to Promote Healthy Meals in Full-Service Restaurants and Canteens: A Systematic Review and Meta-Analysis. Nutrients. 2021;13(4).

116. Maniadakis N, Kapaki V, Damianidi L, Kourlaba G. A systematic review of the effectiveness of taxes on nonalcoholic beverages and high-in-fat foods as a means to prevent obesity trends. Clinicoecon Outcomes Res. 2013;5:519-43.

117. Mayne SL, Auchincloss AH, Michael YL. Impact of policy and built environment changes on obesity-related outcomes: a systematic review of naturally occurring experiments. Obes Rev. 2015;16(5):362-75.

118. McGill R, Anwar E, Orton L, Bromley H, Lloyd-Williams F, O'Flaherty M, et al. Are interventions to promote healthy eating equally effective for all? Systematic review of socioeconomic inequalities in impact. BMC Public Health. 2015;15:457.

119. Metcalfe JJ, Ellison B, Hamdi N, Richardson R, Prescott MP. A systematic review of school meal nudge interventions to improve youth food behaviors. Int J Behav Nutr Phys Act. 2020;17(1):77.

120. Micha R, Karageorgou D, Bakogianni I, Trichia E, Whitsel LP, Story M, et al. Effectiveness of school food environment policies on children's dietary behaviors: A systematic review and meta-analysis. PLoS One. 2018;13(3):e0194555.

121. Molina M, Anderson LN, Guindon GE, Tarride JE. A review of implementation and evaluation of Pan American Health Organization's policies to prevent childhood obesity in Latin America. Obes Sci Pract. 2022;8(3):352-62.

122. Nakhimovsky SS, Feigl AB, Avila C, O'Sullivan G, Macgregor-Skinner E, Spranca M. Taxes on Sugar-Sweetened Beverages to Reduce Overweight and Obesity in Middle-Income Countries: A Systematic Review. PLoS One. 2016;11(9):e0163358.

123. Olstad DL, Teychenne M, Minaker LM, Taber DR, Raine KD, Nykiforuk CI, et al. Can policy ameliorate socioeconomic inequities in obesity and obesity-related behaviours? A systematic review of the impact of universal policies on adults and children. Obes Rev. 2016;17(12):1198-217.

124. Pereira AR, Oliveira A. Dietary Interventions to Prevent Childhood Obesity: A Literature Review. Nutrients. 2021;13(10).

125. Pfinder M, Heise TL, Hilton Boon M, Pega F, Fenton C, Griebler U, et al. Taxation of unprocessed sugar or sugar-added foods for reducing their consumption and preventing obesity or other adverse health outcomes. Cochrane Database Syst Rev. 2020;4(4):Cd012333.

126. Powell LM, Chriqui JF, Khan T, Wada R, Chaloupka FJ. Assessing the potential effectiveness of food and beverage taxes and subsidies for improving public health: a systematic review of prices, demand and body weight outcomes. Obes Rev. 2013;14(2):110-28.

127. Redondo M, Hernandez-Aguado I, Lumbreras B. The impact of the tax on sweetened beverages: a systematic review. Am J Clin Nutr. 2018;108(3):548-63.

128. Roberts KE, Ells LJ, McGowan VJ, Machaira T, Targett VC, Allen RE, et al. A rapid review examining purchasing changes resulting from fiscal measures targeted at high sugar foods and sugar-sweetened drinks. Nutr Diabetes. 2017;7(12):302.

129. Roy R, Kelly B, Rangan A, Allman-Farinelli M. Food Environment Interventions to Improve the Dietary Behavior of Young Adults in Tertiary Education Settings: A Systematic Literature Review. J Acad Nutr Diet. 2015;115(10):1647-81 e1.

130. Sacco J, Lillico HG, Chen E, Hobin E. The influence of menu labelling on food choices among children and adolescents: a systematic review of the literature. Perspect Public Health. 2017;137(3):173-81.

131. Sarink D, Peeters A, Freak-Poli R, Beauchamp A, Woods J, Ball K, et al. The impact of menu energy labelling across socioeconomic groups: A systematic review. Appetite. 2016;99:59-75.

132. Scapin T, Fernandes AC, Curioni CC, Pettigrew S, Neal B, Coyle DH, et al. Influence of sugar label formats on consumer understanding and amount of sugar in food choices: a systematic review and meta-analyses. Nutr Rev. 2021;79(7):788-801.

133. Seyedhamzeh S, Bagheri M, Keshtkar AA, Qorbani M, Viera AJ. Physical activity equivalent labeling vs. calorie labeling: a systematic review and meta-analysis. Int J Behav Nutr Phys Act. 2018;15(1):88.

134. Shangguan S, Afshin A, Shulkin M, Ma W, Marsden D, Smith J, et al. A Meta-Analysis of Food Labeling Effects on Consumer Diet Behaviors and Industry Practices. Am J Prev Med. 2019;56(2):300-14.

135. Shaw SC, Ntani G, Baird J, Vogel CA. A systematic review of the influences of food store product placement on dietary-related outcomes. Nutr Rev. 2020;78(12):1030-45.

136. Silden KE. Impact of competitive foods in public schools on child nutrition: effects on adolescent obesity in the United States an integrative systematic literature review. Glob Health Action. 2018;11(1):1477492.

137. Silva CPd, Bento AC, Guaraldo E. The impact of front-of-the-packaging nutrition labelling warnings on consumer habits: a scoping review exploring the case of the Chilean Food Law. British Food Journal. 2021;124(13):66-80.

138. Sinclair SE, Cooper M, Mansfield ED. The influence of menu labeling on calories selected or consumed: a systematic review and meta-analysis. J Acad Nutr Diet. 2014;114(9):1375-88 e15.

139. Singh A, Bassi S, Nazar GP, Saluja K, Park M, Kinra S, et al. Impact of school policies on non-communicable disease risk factors - a systematic review. BMC Public Health. 2017;17(1):292.

140. Skov LR, Lourenço S, Hansen GL, Mikkelsen BE, Schofield C. Choice architecture as a means to change eating behaviour in self-service settings: a systematic review. Obes Rev. 2013;14(3):187-96.

141. Taillie LS, Hall MG, Popkin BM, Ng SW, Murukutla N. Experimental Studies of Front-of-Package Nutrient Warning Labels on Sugar-Sweetened Beverages and Ultra-Processed Foods: A Scoping Review. Nutrients. 2020;12(2).

142. Teng AM, Jones AC, Mizdrak A, Signal L, Genc M, Wilson N. Impact of sugar-sweetened beverage taxes on purchases and dietary intake: Systematic review and meta-analysis. Obes Rev. 2019;20(9):1187-204.

143. Thow AM, Downs S, Jan S. A systematic review of the effectiveness of food taxes and subsidies to improve diets: understanding the recent evidence. Nutr Rev. 2014;72(9):551-65.

144. Tseng E, Zhang A, Shogbesan O, Gudzune KA, Wilson RF, Kharrazi H, et al. Effectiveness of Policies and Programs to Combat Adult Obesity: a Systematic Review. J Gen Intern Med. 2018;33(11):1990-2001.

145. Van der Horst K, Bucher T, Duncanson K, Murawski B, Labbe D. Consumer Understanding, Perception and Interpretation of Serving Size Information on Food Labels: A Scoping Review. Nutrients. 2019;11(9).

146. van 't Riet J. Sales effects of product health information at points of purchase: a systematic review. Public Health Nutr. 2013;16(3):418-29.

147. Vargas-Alvarez MA, Navas-Carretero S, Palla L, Martinez JA, Almiron-Roig E. Impact of Portion Control Tools on Portion Size Awareness, Choice and Intake: Systematic Review and Meta-Analysis. Nutrients. 2021;13(6).

148. von Philipsborn P, Stratil JM, Burns J, Busert LK, Pfadenhauer LM, Polus S, et al. Environmental interventions to reduce the consumption of sugar-sweetened beverages and their effects on health. Cochrane Database Syst Rev. 2019;6(6):Cd012292.

149. von Philipsborn P, Stratil JM, Burns J, Busert LK, Pfadenhauer LM, Polus S, et al. Environmental Interventions to Reduce the Consumption of Sugar-Sweetened Beverages: Abridged Cochrane Systematic Review. Obes Facts. 2020;13(4):397-417.

150. Wethington HR, Finnie RKC, Buchanan LR, Okasako-Schmucker DL, Mercer SL, Merlo C, et al. Healthier Food and Beverage Interventions in Schools: Four Community Guide Systematic Reviews. Am J Prev Med. 2020;59(1):e15-e26.

151. Whatnall MC, Patterson AJ, Hutchesson MJ. Effectiveness of Nutrition Interventions in Vending Machines to Encourage the Purchase and Consumption of Healthier Food and Drinks in the University Setting: A Systematic Review. Nutrients. 2020;12(3).

152. Wilson AL, Buckley E, Buckley JD, Bogomolova S. Nudging healthier food and beverage choices through salience and priming. Evidence from a systematic review. Food Quality and Preference. 2016;51:47-64.

153. Wright A, Smith KE, Hellowell M. Policy lessons from health taxes: a systematic review of empirical studies. BMC Public Health. 2017;17(1):583.

154. Wyse R, Jackson JK, Delaney T, Grady A, Stacey F, Wolfenden L, et al. The Effectiveness of Interventions Delivered Using Digital Food Environments to Encourage Healthy Food Choices: A Systematic Review and Meta-Analysis. Nutrients. 2021;13(7).
